# PrinTE: a forward simulation framework for studying the role of transposable elements in genome expansion and contraction

**DOI:** 10.1038/s41467-026-78884-0

**Published:** 2026-11-02

**Authors:** Christopher W. Benson, Ting-Hsuan Chen, Susan J. Thomson, Cecilia H. Deng, Shujun Ou

**Affiliations:** 1 Department of Molecular Genetics, Ohio State University, Columbus, OH USA; 2 The New Zealand Institute for Bioeconomy Science Limited, Lincoln, New Zealand; 3 The New Zealand Institute for Bioeconomy Science Limited, Auckland, New Zealand

**Keywords:** Evolutionary genetics, Evolutionary biology, Software

## Abstract

Genome expansion and contraction are driven by transposable element activity, but the underlying dynamics remain enigmatic due to a lack of historical records tracing these changes. Here, we present PrinTE, a forward-time simulator of whole-genome sequences with customizable transposon dynamics for identifying the evolutionary parameters most consistent with an observed genome. Across simulations spanning four orders of magnitude, PrinTE recovers transposon insertion and deletion rates with less than 6% error. We demonstrate PrinTE on *Arabidopsis* and the fungal subdivision Pucciniomycotina, including myrtle rust (*Austropuccinia psidii*), which expanded its genome size to 1,018 Mb through transposon amplifications. Our simulations show that recombination-based removal of LTR retrotransposons alone cannot prevent genome obesity, and that preventing their invasion in the first place is more effective. We hypothesize that *A. psidii* might have experienced a prolonged expansion phase followed by a potent and ongoing period of contraction.

## Abstract (web summary)

The authors present PrinTE, a freely available simulator that evolves genomes forward in time to reveal how transposable elements drive genome expansion and contraction, and use it to test hypotheses about genome size evolution in fungi and plants.

## Introduction

Transposable elements (TEs), or “jumping genes”, are dynamic DNA sequences that replicate and relocate within genomes. TEs constitute 46% of the human (*Homo sapiens*) genome^1^, 85% of the maize (*Zea mays*) genome^2^, and over 90% of the genome of the fungal pathogen *Austropuccinia psidii* that causes the myrtle rust disease^3^. The prevalence of TEs across the Tree of Life reflects their dual roles as both genomic parasites and drivers of evolutionary innovation. Beyond inflating genome sizes, TEs drive structural variation, modulate gene expression, and facilitate chromosomal rearrangements^4^. In both plant and mammalian genomes, bursts of TE activity correlate with speciation events and adaptive radiations, enabling rapid responses to environmental stressors like pathogens or climate shifts^5^. Because TE amplification is considered deleterious to host survival, it has been suggested that TEs and the host defense mechanisms are engaged in an evolutionary arms race. In this process, TE proliferation can trigger TE silencing through epigenetic mechanisms like RNA-directed DNA methylation (RdDM), maintenance methylation by DNA methyltransferases, and the PIWI-piRNA silencing pathway. Additionally, homologous recombination between identical TE sequences can actively remove TEs and downsize the genome.

Recent studies highlight TEs’ contribution to the collective genetic repertoire of a species. Core genomes, conserved across individuals, are often gene-rich and TE-poor^2^, while “variable” or “dispensable” regions are TE-dense and enriched for stress-response genes, pathogen resistance loci, and structural variants^6^. In *Brassica oleracea*, resistance gene analogs cluster in TE-rich variable regions^7^. Similarly, tandem clusters of leucine-rich repeat genes in maize are found in genomic regions rich with young long terminal repeat (LTR) retrotransposons (LTR-RTs)^8^, suggesting that TE-driven structural plasticity contributes to adaptive traits. Related lineages can become karyotypically disparate over generations due to differences in their ability to repress TEs^9^. The global impact of TEs on macro-chromosomal structure plays an important role in evolutionary fates by influencing meiotic stability and similarity between cytotypes^10^. These findings underscore TEs as dynamic regulators of genome evolution, influencing both microevolutionary adaptation and macroevolutionary diversification.

Genome size varies considerably across taxa. Mammalian and bird genomes appear more tightly constrained in genome size than those of both fungi and plants^11^. Numerous studies across the class Mammalia indicate this size constraint is not through a lack of TE radiation bursts^12^ but through mechanistic interplay with purifying selection, where bursts are followed by TE silencing^13^ and DNA loss^12^. Genome size variation in plants facilitates adaptation to specific environments, primarily carried out by whole genome duplication (WGD) and TE expansion^14^, especially LTR-RT expansion^15^. WGD is a common phenomenon in plants where the total number of chromosomes increases, leading to elevated genome size. By contrast, TE expansion occurs gradually as TEs are duplicated from one genomic location to another, leading genome size to increase by the length of the transposition. Extremes of genome size can be observed from the compact genome of *Arabidopsis thaliana* (~135 Mb) through to the giant genomes of the Chinese pine (*Pinus tabuliformis*) at ~25 Gb^16^, lily (*Lilium sargentiae*) at ~36 Gb^17^, and the New Caledonian fork fern (*Tmesipteris oblanceolata*) at ~160 Gb^18^. These are examples of unconstrained genome expansion, with high TE content within intergenic regions and introns. Conversely, there are numerous signs that genome size expansion in plants is subject to constraints and regulatory mechanisms. The *Arabidopsis* genus has undergone several WGDs, yet *Arabidopsis thaliana* remains compact partially through subsequent gene loss that purges redundant paralogs^19^. The dynamics of genome size change have been difficult to accurately evaluate primarily because of the lack of high-fidelity tools for simulation and modeling studies.

Advances in long-read sequencing and computational methods have empowered efforts in resolving TE dynamics^20^, yet reconstructing their evolutionary history remains challenging due to TEs often being fragmented, nested, and highly identical to each other. Simulations of TE evolution are thus indispensable for testing hypotheses about genome diversification, evaluating TE annotation tools, and modeling the interplay between TE activity and selection. By generating synthetic genomes with known TE architectures, including nested insertions, sequence divergence, and polymorphism, researchers can disentangle the stochastic and deterministic forces shaping genome evolution^21^.

Existing TE simulators vary widely in scope and range of biological scenarios that they mimic. Early models, such as those by Clough et al. (1996), introduced foundational concepts by simulating varying modes of mammalian transposition^22^. Modern tools address specific niches. SimulaTE^23^ generates complex TE landscapes through predefined TE architectures, limiting its utility for exploring stochastic evolutionary scenarios. Population-genetic frameworks like SLiM^24^ excel at modeling selection and demography but compress TEs into invariable units, precluding modeling of TE sequence mutagenesis, nested insertion, and the consequential genome size evolution. TESD^25^ incorporates spatial population structure and selection against deleterious insertions, yet its inaccessibility hinders further application. ReplicaTE^26^ engineers synthetic genomes by deleting and reinserting TEs into real assemblies but lacks dynamic iterations through evolutionary epochs. Meanwhile, pMEsim^27^ focuses on simulating polymorphic TE variants for pan-genomes, sacrificing long-term evolutionary dynamics, while simuG^28^ serves as a generic genome simulator that can simulate TE insertions. GARLIC^29^ specializes in generating realistic artificial genomic sequences by modeling intergenic sequences based on k-mer profiles, GC contents, and local repeat landscape, which serve as high-quality negative controls for evaluating annotation tools. TEgenomeSimulator^30^ is a single-timepoint simulator, useful for benchmarking and creating replicas of the TE landscapes of real genomes, but it does not perform forward simulation for complex evolutionary scenarios.

While existing simulation tools collectively address diverse needs, few allow flexibility in genome customization, TE sequence mutagenesis, and dynamic modulation of insertion/deletion rates across evolutionary epochs; none provide granular family-specific controls with iterative simulations for hypothesis testing; and most prioritize synthetic data generation over empirical inference of TE dynamic parameters.

Here, we present PrinTE, a forward-time simulator that evolves whole-genome sequences through millions of generations of TE insertion, deletion, and decay, and infers the TE dynamics most consistent with an observed genome. Tested on a genome with a known history, it recovers the true insertion and deletion rates to within 6% from a search spanning four orders of magnitude. In the fungal subdivision Pucciniomycotina, where genomes vary 70-fold in size, recombination removes LTR-RTs but leaves solo LTRs behind, and our simulations show that this partial removal alone cannot keep a genome small. The smallest genomes instead carry almost no LTR-RTs, suggesting that the most effective way to stay compact is to prevent their invasion in the first place. For the myrtle rust fungus *A. psidii*, PrinTE supports a two-phase history. Our simulations suggest that its genome grew roughly fourfold to more than 1 Gb over 113 million generations and is now shrinking as LTR-RT deletions outpace insertions tenfold. PrinTE offers a quantitative framework for exploring how TEs contribute to genome size expansion and contraction.

## Results

### Introduction of PrinTE

**Fig. 1:**
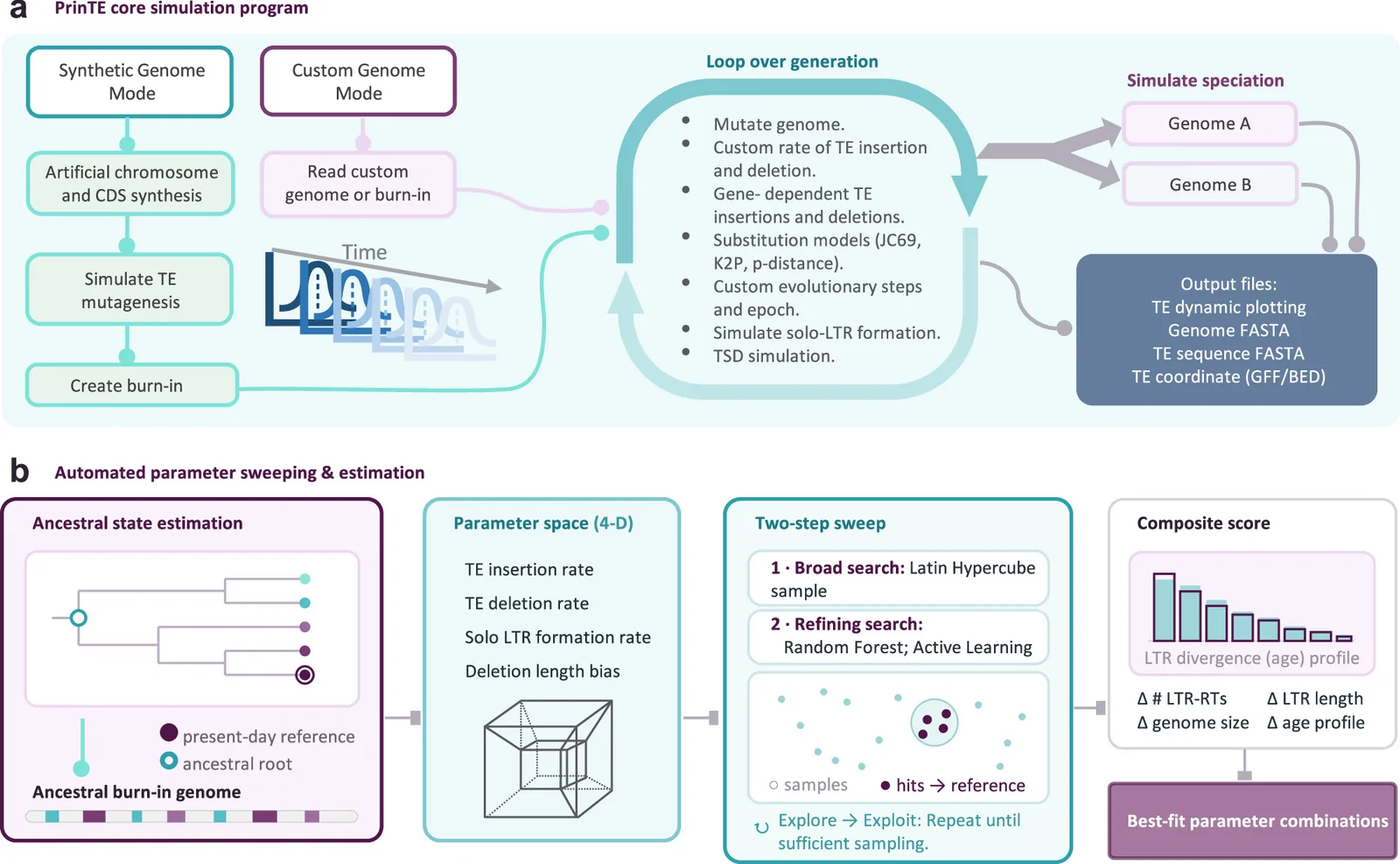
Programmatic components and functionality of PrinTE. **a** The core simulation program. A burn-in genome can be built using the Synthetic Genome Mode or read using the Custom Genome Mode, then evolved forward in user-defined steps that mutate the genome, insert and delete TEs, and form solo LTRs. The Custom Genome Mode allows a run to be split into two lineages to simulate speciation. **b** Automated parameter sweeping and estimation. An ancestral genome is reconstructed from a time-calibrated phylogeny and used as the burn-in for a two-stage sweep of the four-dimensional parameter space, and every simulation is scored against the present-day genome with the Composite Distance Metric to return the best-fitting parameter combinations. See the main text for detailed descriptions of functionality.

PrinTE is a transposon-focused tool that allows dynamic forward simulation of TE insertions, deletions, truncations, and sequence substitutions. PrinTE enables iterative forward simulations to explore complex genome evolutionary scenarios, including the effects of TE dynamics on genome size evolution, genomic divergence, the impact of selection on TE turnover, and speciation (Fig. 1). We primarily demonstrate PrinTE functionality using the fungal subdivision Pucciniomycotina with genome sizes varying by 70-fold, and additionally apply it to survey TE-driven genome size changes in the model plant *Arabidopsis thaliana*. Together, these simulations span fungal and plant kingdoms with wide TE compositions, demonstrating that PrinTE provides a flexible framework for studying TE dynamics across diverse species contexts.

### Forward simulations of TE insertions, deletions, and genome divergence

PrinTE is a forward-time genome simulation tool designed to model the dynamics of TEs within eukaryotic genomes across evolutionary timescales and was developed to incorporate many biologically relevant parameters (Fig. 1a). It uses a modular approach, beginning with either a synthetic genome (Synthetic Genome Mode) or a user-provided genome (Custom Genome Mode) that represents a present-day or theoretical ancestral genome. Users may generate these inputs entirely from scratch with PrinTE’s built-in parameterization tools (synthetic or custom genome), import prior PrinTE runs for continued simulation, or directly use TEgenomeSimulator^31^ outputs. PrinTE implements two methods for simulation: the variable-rate method and the fixed-rate method. The variable-rate method aims to incorporate consequences from some of the biological and selective forces that guide TE evolution. We established several rules in this method: (1) Host genome survival and TE proliferation are conflicting forces pitted against each other; (2) TE superfamilies compete with each other since they occupy the same limited genomic space; (3) The rate of TE insertion is positively correlated with the number of intact TEs in the previous iteration; and (4) TE deletions are guided by the number of TEs that are deleterious to the host’s survival. For this purpose, the simulated genome consists of three components, all of which are receptive to new TE insertions, namely existing TEs, genic space, and intergenic space. If a TE inserts into another TE or intergenic space, there is no cost to the host and no impact on the TE deletion rate. In contrast, TE insertions into genic space are costly to the host, resulting in elevated TE deletion rates in the next generation. Thus, the outcome of the variable-rate method is less predictable, which may lead to generations of genome size increase followed by subsequent generations of decrease. It is useful for those who wish to explore the regulation of TE activities through time and to understand the forces that affect TEs’ bursts and purges.

The fixed-rate method is designed to generate more controlled and predictable outputs. In this method, per-generation TE insertion and deletion rates are predefined by the user, allowing the modeling of known evolutionary trajectories and the generation of results that more closely match expectations (Supplementary Fig. 1). In practice, forward simulation can begin from a simulated genome that mirrors a present-day version and forecast a few million generations of TE evolution into the future. Alternatively, it can start from an ancestral genome and simulate it to the present day. This approach allows users to test hypotheses relating to TE bloat and purge and to superimpose TE evolutionary narratives onto the species of interest. A key assumption of the fixed-rate method is that TE insertion and deletion rates remain constant throughout the simulation period. We acknowledge that TE activity can fluctuate through time in response to environmental change, host regulatory evolution, demographic shifts, and episodic transposition bursts. The simplification of TE activity fluctuations in PrinTE provides a controlled framework for modeling the effective average rates over the simulated interval. Users interested in temporal rate heterogeneity can reduce the number of generations simulated per iteration and adjust insertion and deletion parameters between successive runs using PrinTE’s continuation functionality, thereby approximating TE dynamics variability across evolutionary time.

Independent of the rate model chosen, new elements are placed according to the transposition mode. Class I (copy-and-paste) elements such as LTR-RTs amplify replicatively, whereas Class II (cut-and-paste) DNA transposons excise before re-inserting. Because DNA transposons can gain copies when excision is coupled to DNA replication or repair, PrinTE treats the expected number of successful re-insertions per excision as a user-defined parameter (“--cutpaste_reinsertion”). Insertions may land within existing elements to generate the nested arrangements that pervade TE-rich genomes. Each insertion is flanked by a target site duplication (TSD) whose length matches its superfamily. PrinTE simulates deletions based on the common routes of TE loss. An intact LTR-RT may be removed partially, entirely, or collapse into a solo LTR. PrinTE defines any TE that is not intact or a solo LTR as fragmented. The TE deletion and solo LTR conversion procedure preferentially acts on longer elements, reflecting the length-biased decay driven by homologous recombination. Because PrinTE records the exact coordinates, insertion time, and ancestry of every element it places, each simulated genome is accompanied by a ground-truth annotation that records per-element TSD, nesting relationships, solo versus intact status, and LTR-LTR sequence divergence as an age proxy.

PrinTE can simulate a single generation, which introduces subtle changes to the genome. To allow for simulating millions of generations, PrinTE employs a stepwise approach in which it can simulate TE evolution of a number of generations in steps until it reaches the total number of generations specified by the user. To approximate TE mutation in multiple generations, PrinTE uses a normalized exponential decay distribution to guide DNA substitutions, mimicking the natural turnover of TE sequences that would occur during iterative steps.

In addition to simulating a single trajectory, PrinTE can automatically search for the evolutionary parameters that best model the genome of interest (Fig. 1b). For this, PrinTE reconstructs the genome’s ancestral state and searches for the TE conditions that best recreate the present-day genome. The user defines a parameter space spanning insertion rate, deletion rate, solo LTR formation rate, and deletion length bias, which PrinTE searches systematically using Latin hypercube sampling and active learning in broad and refining stages (the Grid Search Method; Methods). The refining stage trains a surrogate model on the results gathered from the broad searching stage to propose the most informative parameter combinations for subsequent tests. Each candidate simulation is automatically scored against the reference genome using a Composite Distance Metric that combines LTR-RT copy number, cumulative LTR-RT length, the LTR-RT divergence distribution, and genome size. The single best-fitting simulation and best 5% range are reported. This reproducible procedure requires no manual tuning and efficiently identifies the TE turnover rates most consistent with the genome of interest under PrinTE’s model. Finally, PrinTE reports a sensitivity analysis that ranks each parameter by how much of the variation in the composite score it explains, identifying which TE parameters most strongly influence the fit.

PrinTE represents a state-of-the-art simulation of TE dynamics in eukaryotic genomes, enabling the modeling of TE contributions to genome evolution and the exploration of past and future genome evolution trajectories.

### Performance and benchmarking

The 5′ and 3′ LTR regions of an intact LTR-RT are identical upon transposition. Under the molecular clock hypothesis, mutations to the LTRs reflect the amount of time since transposition^32,33^. PrinTE introduces point mutations to simulated genomes but does not track them. To test its mutation machinery, we developed a k-mer-based LTR-RT dating approach, called Kmer2LTR (https://github.com/cwb14/Kmer2LTR), which accurately detects mutations to the LTR region of highly divergent LTR-RTs, allowing us to evaluate PrinTE’s accuracy in modeling point mutations and its ability to forward-evolve a genome. PrinTE allows users to customize the transition/transversion ratio (Ti/Tv), which enables evaluating various nucleotide substitution models. We also integrate these substitution models into Kmer2LTR, allowing for benchmarking PrinTE’s mutational models and evaluating model mismatches. For comparisons, we used SLiM to simulate genome divergence based on a simple neutral model. Single-nucleotide polymorphisms (SNPs) were projected onto the SLiM-simulated genealogies to obtain whole-genome sequences that were comparable to PrinTE simulations. Divergence of SLiM-simulated genomes was estimated based on mutations detected using Minimap2 whole-genome alignments.

**Fig. 2:**
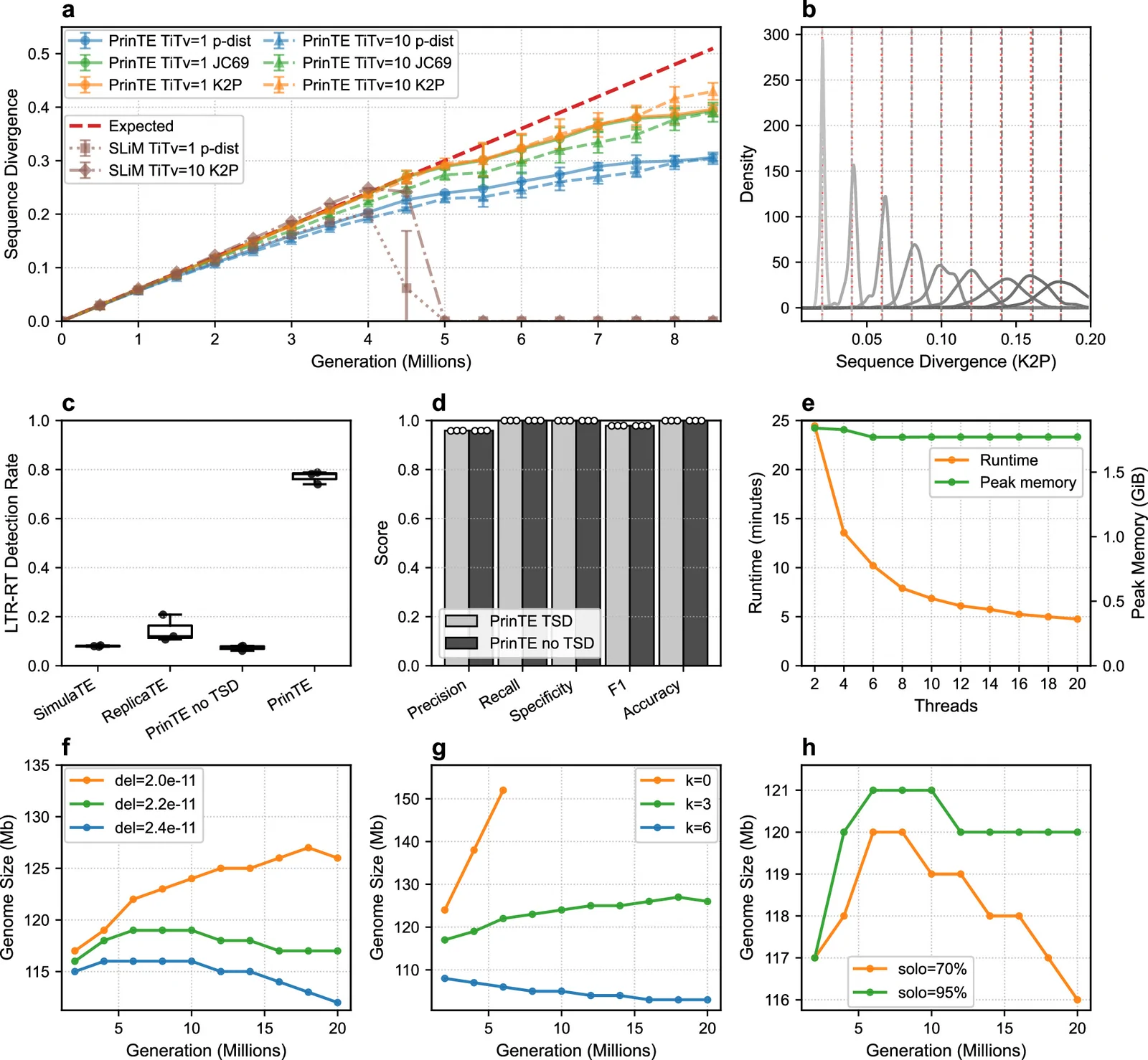
Benchmarking PrinTE. **a** Estimation of LTR retrotransposon divergence simulated by PrinTE with p-distance (p-dist), Jukes-Cantor 69 (JC69), and Kimura two-parameter (K2P) DNA substitution models and transition/transversion ratios (Ti/Tv) of 1 and 10. Single-nucleotide polymorphisms were projected onto SLiM-simulated genealogies and detected using Minimap2 whole-genome alignment. The red dashed line shows the true divergence; the SLiM/Minimap2 estimates fall to zero beyond five million generations, where the whole-genome alignment fails. Points show mean ± s.d. of *n* = 3 independent simulations; each PrinTE value is the divergence pooled over all intact LTR-RTs in one simulated genome. **b** Density distribution of LTR sequence divergence simulated under various divergence scenarios and estimated by Kmer2LTR. Vertical gray dashed lines show the cumulative sequence divergence of all LTR-RTs in the simulated genome. Vertical red dashed lines show the expected sequence divergence at each time point. **c** LTR-RT detection rates by LTR_retriever using three TE simulators, including PrinTE genomes with their TSDs removed as a control. TSD, target site duplication. Box plots: center, median; bounds, 25^th^-75th percentiles; whiskers, minimum-maximum; dots are *n* = 3 independent simulations. **d** Performance metrics of LTR-RT identification by RepeatMasker using the PrinTE library and the simulated genome as input, with and without TSDs. Bars show mean ± s.d. of *n* = 3 independent simulations; scores are per base pair. **e** PrinTE runtime and memory utilization using different numbers of CPU threads. Genome size across generations under varying deletion rates (**f**), deletion length bias parameter k (**g**), and solo LTR formation rate (**h**). Source data are provided as a Source Data file.

In all comparisons, the Kimura two-parameter (K2P) model outperformed p-distance estimates, regardless of the Ti/Tv ratio (Fig. 2a). The one-parameter Jukes-Cantor 69 (JC69) model performed as well as K2P when Ti/Tv=1, but underperformed when Ti/Tv=10 (Fig. 2a). PrinTE and SLiM/Minimap2 both accurately detect mutations up to 25% sequence divergence, but the latter declines sharply at divergences exceeding 25%. PrinTE’s Kmer2LTR approach benefits from pre-determined LTR boundaries, which allow it to force a global sequence alignment onto the otherwise highly divergent LTRs and accurately detect mutation percentages up to 30%. Beyond 30%, Kmer2LTR still detects divergence but slightly underestimates it (Fig. 2a). Simulations at higher generation counts lead to a wider range of sequence divergence in LTR-RTs, but the overall divergence estimate remains centered on the expected values even at the relatively high divergence range (Fig. 2b). These results demonstrate a robust LTR-RT divergence estimation by PrinTE’s Kmer2LTR method, especially when using the K2P model.

Next, we assessed the simulation fidelity of PrinTE and two existing TE simulators, SimulaTE and ReplicaTE. The success of structure-based TE annotation depends on the presence of known sequence structures and motifs. We reasoned that intact TEs in simulated genomes should be detectable using a structure-based annotation approach. However, existing TE simulation tools often fail to generate TSDs, a key signature of LTR-RT integration^32^. Our results show that LTR-RTs simulated by PrinTE have a markedly higher detection rate than those simulated by ReplicaTE and SimulaTE (77% vs 15% and 8%, respectively), suggesting that PrinTE better reflects the biological features that compose a TE (Fig. 2c). Removing the TSDs from PrinTE-simulated genomes lowered the detection rate to the level of the other simulators (Fig. 2c, “PrinTE no TSD”), showing that reproducing the TSD is what makes the simulated elements structurally recognizable. We used a PrinTE-derived TE library to annotate the simulated genomes and evaluated the result against the PrinTE-generated ground truth. Quality metrics were strong with and without TSDs (F1 = 0.978), indicating that the output TE library and corresponding genome were accurately simulated (Fig. 2d).

To evaluate the computational performance of PrinTE, we simulated one million generations of TE evolution in a 100 Mb genome using a step size of 100,000 generations. The initial genome was seeded with 6000 TEs, and each subsequent iterative step resulted in insertion and deletion of ~500 TEs. PrinTE demonstrated efficient memory usage with peak memory less than 2 GB. PrinTE runtimes scale well at low thread counts and begin to saturate with increased thread allocation (Fig. 2e). These performance metrics demonstrate that PrinTE is highly efficient, optimized for large-scale analysis, and capable of running on personal computers or servers.

Next, we profiled three deletion-related parameters in PrinTE to contextualize their impact on genome size, namely deletion rate, deletion length bias, and solo LTR formation rate (Fig. 2f–h). Deletion rate controls the number of TEs that are processed by PrinTE’s deletion engine. Expectedly, we observed decreasing genome size with increasing deletion rates (Fig. 2f). Deletion of TEs is mainly achieved by homologous recombination between identical TE sequences. In PrinTE, we allow users to set a deletion length bias that scales exponentially with TE length using the “--k” parameter, which can approximate the disproportionate impact of unequal recombination on purging longer TEs. Larger k biases deletion more strongly toward longer TEs and thus constrains genome size more tightly (Fig. 2g, Supplementary Fig. 2). When k is set to 0, TE deletion has no length bias, allowing genome size growth through the accumulation of long TEs (Fig. 2g, Supplementary Fig. 2).

Intra-element homologous recombination between LTR regions of individual LTR-RTs will partially remove the element and create solo LTRs as remnants. Given a fixed deletion rate, PrinTE can model the proportion of LTR-RT deletions that go through this process by implementing the “--solo_rate” parameter. Compared to full-element deletions, an increased solo LTR formation rate can increase the number of solo LTRs in the genome, resulting in a larger genome size (Fig. 2h, Supplementary Figs. 3, 4). By adjusting parameters of deletion length bias and solo LTR formation rate that interplay with TE insertion and deletion rates, we can create complex scenarios for modeling genome size evolution in different systems.

### Genome size evolution through TE expansion and contraction

**Fig. 3:**
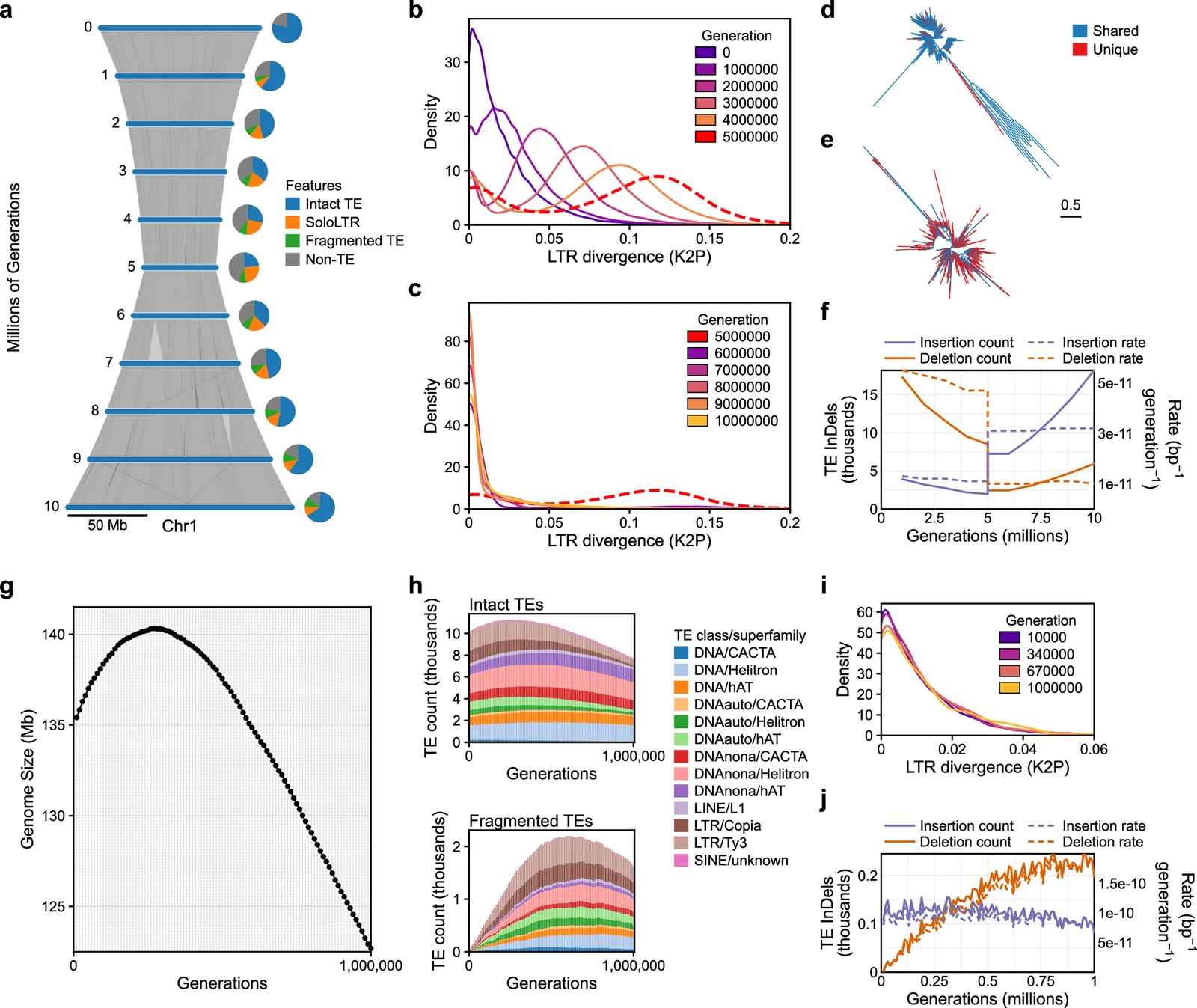
Modeling TEs’ contribution to genome size evolution using fixed (a-f) and variable (g-j) rate methods. **a**–**f** Ten million generations of LTR-RT evolution exploring their long-term impact on genome size using the fixed-rate method. For demonstration, we set a rate of TE deletion that exceeded insertion by fourfold in the first five million generations, then reversed the rates by setting the insertion rate to exceed the deletion rate by fourfold over the next five million generations. **a** A syntenic ribbon plot illustrates the change in chromosome size through time. Pie charts corresponding to each time point show the relative contribution of each feature to genome size. **b,c** Density distributions of LTR-RT divergence estimated using the K2P model during the first five million generations (**b**; genome size decrease) and the second five million generations (**c**; genome size increase). Five million generations is the transition between genome size decrease and increase, which is shown in both plots as dashed red lines. Phylogenetic trees show the evolutionary relationship of LTR-RTs from the *Tekay* family after five million generations of genome size decrease (**d**) or five million generations of genome size increase (**e**). Branches are colored by whether an element was inherited from the common ancestor (shared, blue) or inserted after the lineages separated (unique, red). **f** LTR-RT insertion and deletion counts (solid lines) and rates (dashed lines) across 10 million generations. **g–j** A simulated genome replica of *Arabidopsis thaliana* is forward-evolved for one million generations using PrinTE’s variable-rate method. **g** Genome size change. **h** Intact (top) and fragmented (bottom) TE counts per superfamily across one million generations. **i** The distribution of LTR-RT divergence estimated using the K2P model. **j** TE insertion and deletion counts (solid lines) and rates (dashed lines) illustrate the self-adjusting functionality of PrinTE’s variable-rate method. Source data are provided as a Source Data file.

LTR-RTs are the primary contributing TEs to genome size in many plant, fungal, and animal genomes^34^. To isolate the effect of TEs on genome size across temporal gradients, we focused on modeling LTR-RT evolution using PrinTE’s fixed-rate method to simulate predictable patterns of TE expansion and contraction. Here, we simulated two dynamic scenarios sequentially on the same genome. The first scenario used a TE deletion rate fourfold higher than that of insertions. We observed a 45% decrease in genome size after five million generations of forward simulation (Fig. 3a). The decrease was initially driven by a swift reduction in the number of intact TEs (Supplementary Fig. 5a) followed by accumulation of fragmented TEs and solo LTRs (Fig. 3a, Supplementary Fig. 5b).

Following the genome size decrease, we continued the simulation for an additional five million generations but reversed the TE insertion rate to fourfold higher than that of deletions. The elevated insertion rate caused genome bloating (Fig. 3a), powered by an increase in the numbers of both intact and fragmented TEs (Supplementary Fig. 5c, d). The sequence lengths of solo LTRs and of fragmented and intact LTR-RTs shifted in response to purging pressures from the host (Supplementary Fig. 6). Interestingly, because k > 0 leads to a higher probability of deleting longer LTR-RTs, the length of intact LTR-RTs was overall reduced in the genome size decrease phase and slowly increased in the genome size increase phase when purging pressure dropped (Supplementary Fig. 6).

During simulations of genome size decrease, when the rate of TE deletions exceeded that of insertion, we observed unbiased removal of LTR families through generations. Accumulation of DNA substitutions and reduced LTR-RT insertions led to elevated sequence divergence of the remaining pool of intact LTR-RTs and a shift in the divergence distribution across time (Fig. 3b). There were two distinct density peaks as the simulation proceeded, even though the rate of TE insertions and deletions remained constant (Fig. 3b). The smaller peak represents younger LTR-RTs that were inserted recently and have had less time to be purged, while the larger peak that shifts to the right and decreases in density through time was retained from the standing pool of LTR-RTs in the ancestral genome. In cases where the rate of TE insertions exceeds deletions, young LTR-RTs quickly proliferate in high abundance and dominate the density distribution of LTR-RTs in the simulated genome, masking the signal of remaining older LTR-RTs (Fig. 3c). We conclude that the divergence distribution of intact LTR-RTs reflects their insertion and deletion dynamics through time.

When species become reproductively isolated from each other, their TEs are also isolated and evolve independently. Genomes share TEs that derive from their common ancestor and also contain novel insertions that are unique to each. With PrinTE, we simulated a speciation event by evolving a genome for a period of time, and then diverting it into two isolated evolutionary paths with shifted TE dynamics to create divergent trajectories of genome expansion or contraction (Fig. 3d, e). In the contracting genome, there were relatively few unique TEs created after speciation (Fig. 3d; Supplementary Fig. 7). In contrast, the expanding genome had a higher abundance of young and unique TEs (Fig. 3e; Supplementary Fig. 7). In the fixed-rate method, the rate of TE insertion and deletion remains relatively stable, only deviating by the stochastic variability introduced by sampling a Poisson distribution, while the number of insertions and deletions introduced scales with the size of the genome (Fig. 3f).

Recognizing that the rates of TE insertion and deletion can be dynamic through time and may be modified by varying biological processes, we implemented the variable-rate method in PrinTE. This method can be used to explore specific molecular features that constrain TE evolution and its impact on genome size, including TE copy number, the proportion of intact versus fragmented elements, TE age, TEs’ proximity to genes, and chromatin state. Here, rather than setting fixed TE insertion and deletion rates, we start with an initial rate and allow the tool to dynamically determine subsequent insertion and deletion rates based on the composition of the most recently simulated genome (Fig. 3j). We simulated the genome size and TE evolution of present-day *Arabidopsis thaliana* to forecast TE dynamics over one million generations using the variable-rate method. The simulation was initialized with the observed genome size (~135 Mb), chromosome number (*n* = 5), TE composition (~21%), gene number, and nucleotide substitution rate (7 × 10^-9^ substitutions per bp per generation^35^). Under the variable-rate simulation, the genome had an initial phase of expansion followed by a prolonged period of genome size contraction (Fig. 3g). Expectedly, genome size increased through the accumulation of both intact and fragmented TEs and decreased due to the reduction of intact TEs (Fig. 3h). The divergence of intact LTR-RTs reflects the genome size expansion and contraction (Fig. 3i). These features allow us to simulate TE dynamics close to what is observed in eukaryotic genomes.

### Genome size evolution of rust fungi

We further studied the impact of TE dynamics on genome size evolution using rust fungal genomes, which span a wide range of genome sizes and TE compositions. The Pucciniomycotina is a subdivision of fungi that contains 10 classes and over 8,400 species^36^. We downloaded 58 Pucciniomycotina fungal genomes from NCBI and JGI MycoCosm^37^ to investigate genome expansion and contraction (Supplementary Data 1). We identified and removed alternative haplotypes from these genomes using Redundans^38^. After filtering for assembly contiguity (contig N50 > 100 kb; Methods), 47 genomes were retained (Supplementary Data 2). The resulting haploid genome size varies from 13.6 Mb (*Mixia osmundae*) to 1,018.1 Mb (*Austropuccinia psidii*), representing over 70-fold differences (Supplementary Data 2). We performed Benchmarking Universal Single-Copy Ortholog (BUSCO) evaluations using compleasm^39^ and the odb12 *Basidiomycota* database. The complete BUSCOs (single and duplicated) averaged 90.7% (SD = 5.4), and contig N50 averaged 846 kb (ranging from 111 kb to 3.8 Mb), suggesting high assembly quality of haploid genomes (Supplementary Data 2).

**Fig. 4:**
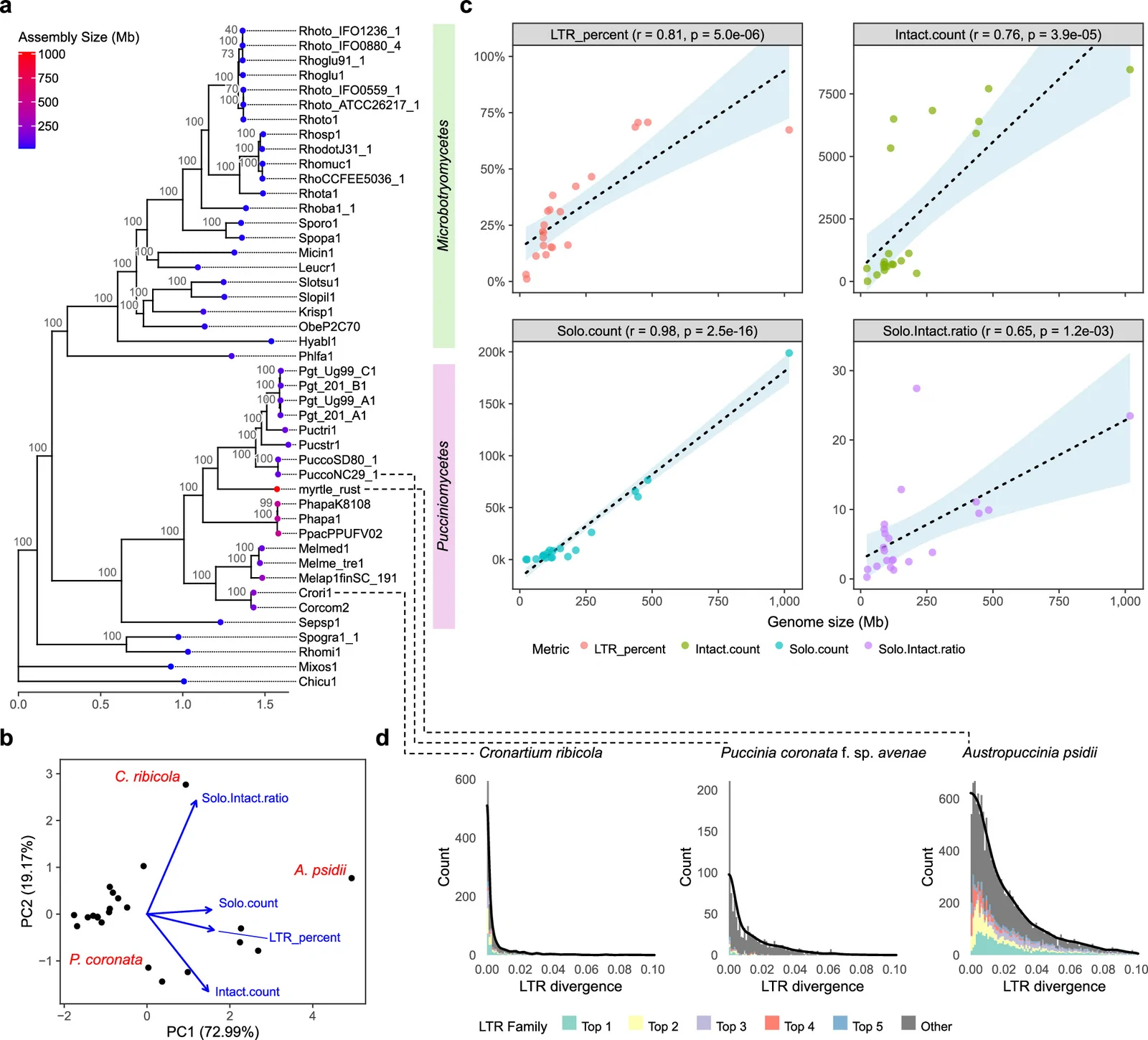
LTR retrotransposon dynamics shape genome size variation across Pucciniomycotina. **a** The phylogeny of Pucciniomycotina fungal genomes used in this study. Protein sequences of shared single-copy BUSCOs were used for tree inference with 1000 bootstrap replicates. Bootstrap support percentage is shown on each branch. The two classes, *Pucciniomycetes* and *Microbotryomycetes*, are labeled beside the phylogeny. The genome size of each species is indicated by colored dots. **b** Principal Component Analysis (PCA) on LTR metrics in fungal genomes. Each dot represents one genome. PCA loadings of each metric are shown. Genomes selected for further studies are labeled. **c** Pearson’s correlation (*r*) between genome size and LTR sequence content (LTR_percent), intact LTR-RT count (Intact.count), solo LTR count (Solo.count), and solo:intact ratio. Each dot represents one genome. Each panel title shows *r* and *P* from a two-sided *t* test (*n* = 22 genomes, d.f. = 20), unadjusted for multiple comparisons. Confidence intervals are provided in the Source Data file. Dotted lines show least-squares fits with shaded 95% confidence bands. **d** Divergence of intact LTR-RTs in *Cronartium ribicola* (white pine blister rust), *Puccinia coronata* f. sp. *avenae* (oat crown rust), and *Austropuccinia psidii* (myrtle rust) genomes. The top five most abundant LTR families in each genome are colored, and they are not shared between genomes. A density line is shown as the contour in each plot. The locations of these species on the phylogeny are indicated by dotted lines. Source data are provided as a Source Data file.

We identified the shared single-copy BUSCOs and used IQ-TREE^40^ to construct the phylogeny of Pucciniomycotina. The resulting phylogeny was dominated by two classes, *Microbotryomycetes* and *Pucciniomycetes* (Fig. 4a). *Microbotryomycetes* genomes are small, ranging from 17.6 Mb to 50.3 Mb. Correspondingly, on average, only 0.4% of the genome consisted of LTR sequences, with a range of 0–3.1% (Supplementary Data 2). This observation suggests that the best strategy for controlling genome size is to avoid the invasion of LTR-RTs. *Pucciniomycetes* have widely distributed genome sizes, ranging from 26.8 Mb (0.4% LTR) to 1,018.1 Mb (67.4% LTR) (Supplementary Data 2).

To further study the dynamics of LTR-RTs in these fungal genomes, we de novo identified intact LTR-RTs and solo LTRs, and calculated the whole-genome solo:intact ratio using LTR_retriever^32^. Using genomes with >1% LTR sequence content (*n* = 22), we performed Principal Component Analysis (PCA) on LTR metrics and genome size. The analysis revealed clustering among smaller genomes, whereas larger genomes appeared as outliers, including *Cronartium ribicola* (white pine blister rust), *Puccinia coronata* f. sp. *avenae* (oat crown rust), and *Austropuccinia psidii* (myrtle rust; Fig. 4b). PCA loadings suggested that solo LTR count, intact LTR-RT count, and their ratio all contributed to the outlier status of the large genomes (Fig. 4b). Including total LTR content, these LTR metrics showed strong positive correlations with genome size (Pearson’s *r* ≥ 0.65, *p* ≤ 0.002; Fig. 4c). We then performed a multivariate regression using the four variables in Fig. 4c and the haploid genome size, and found that the solo LTR count was the only significant variable (Multiple *R*^2^ = 0.98, *p* = 2.44 × 10^–10^). This observation supports LTR-RTs’ major role in impacting genome size upon successful invasion.

Not surprisingly, intact LTR-RTs, as the direct product of retrotransposition, were strongly correlated with genome size (*r* = 0.76; Fig. 4c). However, solo LTR sequences, which arise through partial removal of LTR-RTs^32^, showed an even stronger correlation and were almost linearly correlated with genome size (*r* = 0.98; Fig. 4c). We also observed a positive correlation between genome size and solo:intact ratio, a metric frequently used as a proxy to measure the intensity of LTR-RT purging through conversion to solo LTR^33^. This pattern appears inconsistent with the conventional interpretation that higher solo:intact ratios reflect more extensive LTR-RT removal and therefore should be associated with smaller genomes^33,41^. Because the solo:intact ratio depends on how completely solo LTRs and intact elements are annotated, and elevated ratios may also arise from differential retention of solo LTRs or from lineage-specific differences in TE turnover, we used the solo:intact ratio as a descriptive summary of LTR-RT composition rather than as a measure of purging pressure, and it is not among the statistics used to score PrinTE simulations. Improved methods for annotating solo LTRs and intact elements, together with explicit modeling of TE birth-death dynamics, will be important for disentangling these alternative explanations. Independently of this metric, intra-element recombination is an inherently partial mode of removal, because solo LTRs remain in the genome after recombination. In controlled simulations where the rate of solo LTR formation is set directly, raising it at a fixed deletion rate produced larger instead of smaller genomes (Fig. 2h, Supplementary Figs. 3, 4), indicating that recombination-mediated removal alone is not sufficient to constrain genome expansion.

We selected *C. ribicola*, *P. coronata* f. sp. *avenae*, and *A. psidii* as representatives of three distinct routes of genome size evolution. *C. ribicola* has a genome size of 211.6 Mb, with the LTR divergence distribution showing a quick turnover time for LTR-RT insertions (Fig. 4d; Supplementary Fig. 8), suggesting a quick-insertion, quick-removal mode. *P. coronata* f. sp. *avenae* has a genome size of 113.2 Mb, and the LTR divergence distribution showed a much slower turnover time for LTR-RT insertions (Fig. 4d; Supplementary Fig. 8), suggesting a slow insertion-slow removal mode of action. *A. psidii* has a large genome size of 1,018.1 Mb. However, the LTR divergence distribution of *A. psidii* shows peak LTR-RT insertion 1.4 million generations ago (Fig. 4d; Supplementary Fig. 8), suggesting a recent slowdown of insertion activity^8^ and an ongoing decrease in genome size. We used PrinTE to test these three LTR-RT dynamic hypotheses and to evaluate the parameters that may have shaped the evolution of these three species.

### Simulating digital replicas of rust fungal genomes

**Fig. 5:**
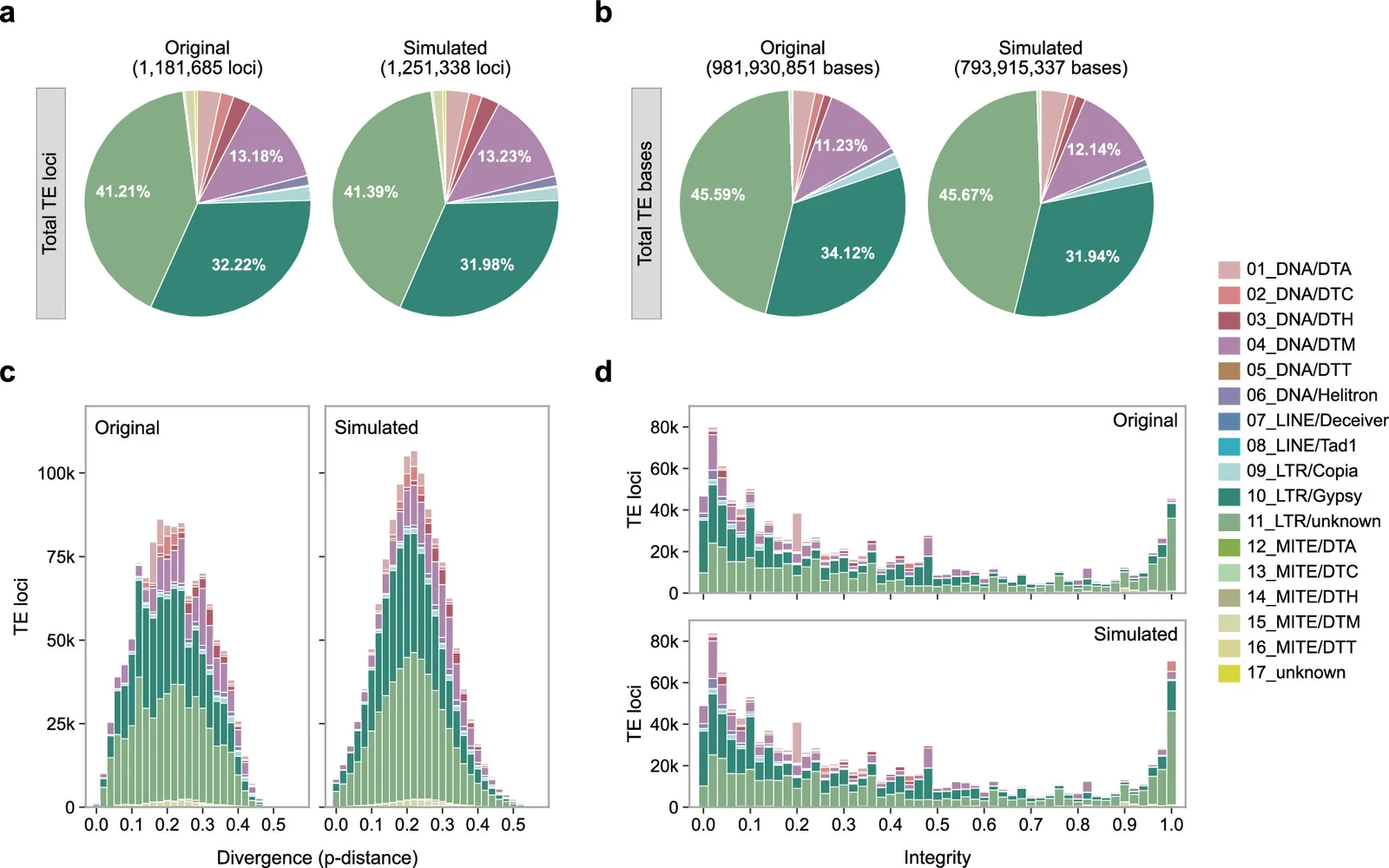
TE composition approximation for the *Austropuccinia psidii* genome. Superfamily composition of all annotated TE loci (**a**) and TE bases (**b**) in the original and simulated genomes. The total number of loci is given above each pie chart. **c** Distribution of TE sequence divergence relative to the TE library. **d** Distribution of TE sequence integrity, which is defined as the length of each copy relative to its consensus sequence. Source data are provided as a Source Data file.

Accurate simulation of TE dynamics requires a faithful starting representation of the real genome, which varies substantially across TE families and species because of their complex and diverse evolutionary histories. This variability makes it challenging to simulate genome-wide TE landscapes that can be used for modeling and hypothesis testing. To address this, we used TEgenomeSimulator^31^ with its TE Composition Approximation Mode to model the TE profiles of *C. ribicola*, *P. coronata* f. sp. *avenae*, and *A. psidii* genomes within the Pucciniomycotina subdivision (Fig. 5, Supplementary Figs. 9, 10). The sizes of these simulated genomes closely matched their actual genome sizes. Similarly, the TE proportion of the simulated genomes closely approximated the observed values in terms of TE loci and TE bases per superfamily (Fig. 5a, b, Supplementary Figs. 9a, b, 10a, b). TEgenomeSimulator estimated the sequence divergence and integrity distributions of each TE family in the real genomes for the simulation of digital replicas. The simulator uses normal distributions to smooth out small-scale fluctuations present in the real genome, producing a simulated divergence profile that broadly resembled the real data (Fig. 5c, Supplementary Figs. 9c, 10c).

TEs can break down over time due to nested insertions, recombination, small deletions, and other processes. The whole-genome distribution of TE integrity clearly reflects the turnover rate of TEs in the three selected fungal genomes (Fig. 5d, Supplementary Figs. 9d, 10d). The *C. ribicola* genome contains mostly high-integrity TEs, suggesting a high rate of TE turnover and active TE insertions (Supplementary Fig. 9d). In contrast, the *P. coronata* f. sp. *avenae* genome harbors highly fragmented TEs and very few intact TEs, suggesting a low activity profile (Supplementary Fig. 10d). The myrtle rust genome, *A. psidii*, presents a bimodal distribution in TE integrity, suggesting that both heavy bloating and purging may have been prominent drivers in the evolutionary history of the genome (Fig. 5d). The digital replicas of these genomes have distributions of TE integrity that are almost identical to those of the real genomes (Fig. 5d, Supplementary Figs. 9d, 10d). These simulations yield generalized distribution patterns that capture the major trends in TE sequence evolution of each genome, providing a robust foundation for PrinTE’s simulations of TE bloating or purging episodes, effectively serving as burn-in phases for modeling genome evolution.

### Assessing genome evolution with forward simulations of LTR activities

PrinTE effectively captures LTR-RT dynamics in the divergence distribution (Fig. 2a, b; Fig. 3b, c), which can subsequently be used to evaluate hypothesized LTR-RT dynamics. PrinTE supports forward simulation from past to present, starting from a computationally reconstructed ancestral genome and searching for the conditions that lead to the present-day genome. With the present-day genome serving as ground truth, the goodness of fit can be scored and maximized directly. The resulting best-fit parameters identify the TE insertion and deletion rates most consistent with each lineage’s observed genomic architecture, providing a quantitative hypothesis on TE activity levels over the course of fungal genome evolution.

**Fig. 6:**
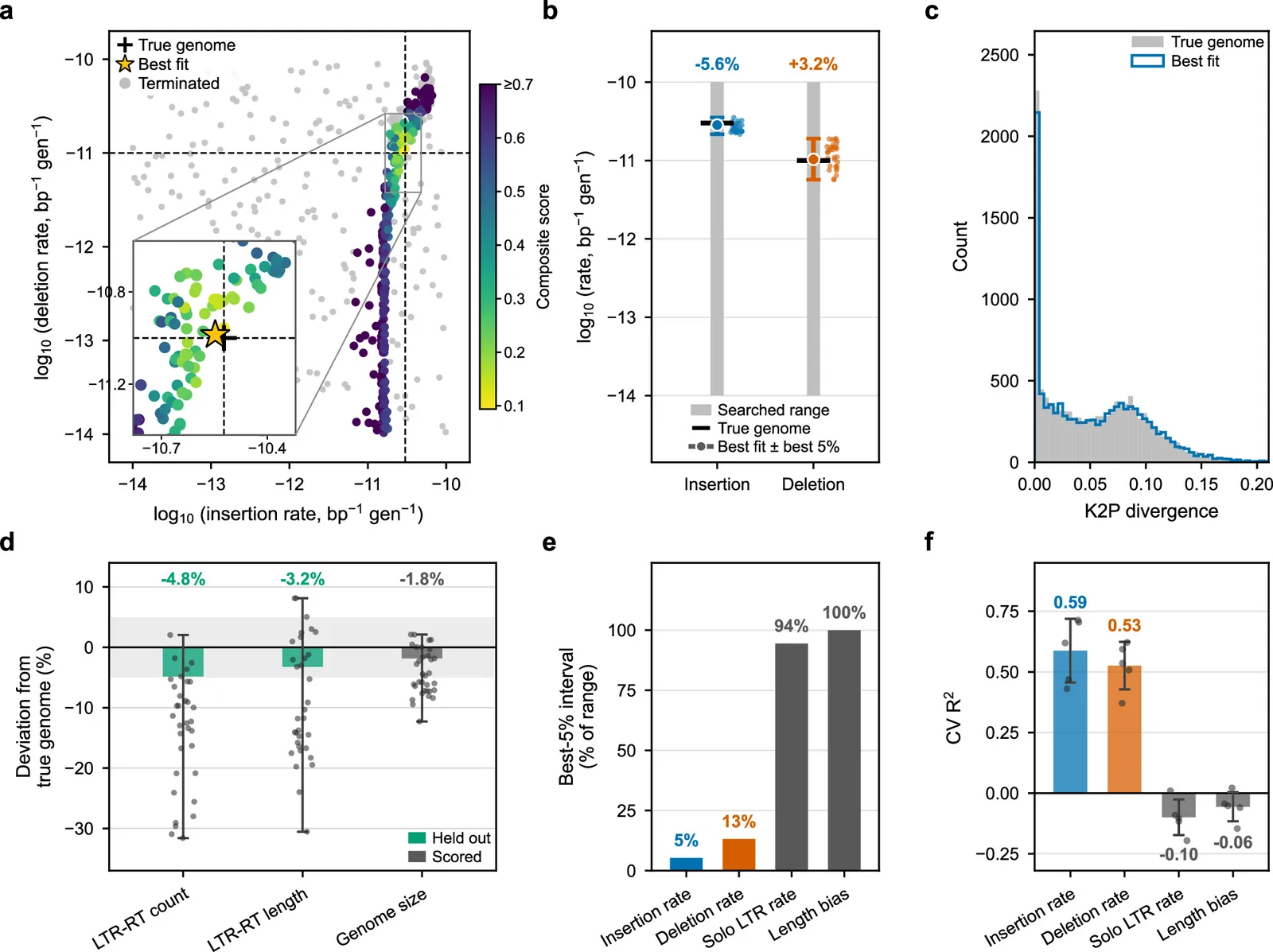
Parameter recovery test. A 100 Mb genome was forward-evolved with PrinTE for three million generations under known insertion and deletion rates (3.0×10^−11^ and 1.0×10^-11^ per bp per generation, respectively), solo LTR formation rate (95%), and deletion length bias (k = 10). The resulting genome was treated as an empirical reference (“true genome”) and passed to the Grid Search Method, which was given no knowledge of the parameters that produced it. **a** The 650 parameter combinations were evaluated and colored by composite score, where lower scores indicate a closer match to the truth. Gray points (“terminated”) are simulations that departed the genome size window before finishing and were terminated without scoring. Dashed lines and the crosshair mark the true rates, the gold star marks the best fit, and the inset magnifies the boxed region. **b** Recovered rates against the true rates. Gray bars show the range searched, the black tick marks the true genome’s rate, and the colored point shows the best-fit simulation. Dots show the best 5% of individual simulations (*n* = 33 of 650) and error bars show their minimum to maximum. Percentages show the deviation of the best fit from the true rate. **c** Divergence distribution of intact LTR-RTs in the true genome (gray) and in the best-fit simulation (blue), both annotated de novo. **d** Deviation of the best-fit genome from the true genome. “Held out” are features not used in composite score calculation and contain 11,423 vs 10,871 intact LTR-RTs, and 39.1 vs 37.9 Mb of cumulative LTR-RT length for the true and the best-fit simulation, respectively. Bars show the best-fit genome, error bars span the minimum to maximum across the best 5% of simulations (*n* = 33), and the shaded band marks ±5%. **e** Width of the best-5% interval for each parameter as a percentage of the range searched. Smaller values indicate a better-constrained parameter. **f** Cross-validated (CV) R^2^ measures how much of the variation in the composite score each parameter explains by itself. Bars show mean ± s.d. of the *n* = 5 cross-validation folds. Source data are provided as a Source Data file.

We next asked if we could recover a known LTR-RT evolutionary history from a simulated genome size evolution. We used PrinTE to forward-evolve a 100 Mb genome for three million generations in 500,000-generation steps under a known combination of insertion rate, deletion rate, solo LTR formation rate, and deletion length bias, which expanded the genome to 127.6 Mb. We then treated the resulting genome as an empirical reference (“true genome”), annotated its intact LTR-RTs de novo as we would for a real genome, and ran the Grid Search Method from the same generation-0 burn-in over insertion and deletion rates spanning four orders of magnitude. Across 650 parameter combinations, the best-fitting simulation identified an insertion rate of 2.83 × 10^–11^ per bp per generation (best-5% range: [2.2 × 10^–11^, 3.5 × 10^–11^]), against a true value of 3.0 × 10^–11^, and a deletion rate of 1.03 × 10^–11^ (best-5% range: [5.7 × 10^–12^, 1.9 × 10^–11^]), against a true value of 1.0 × 10^–11^. Both ranges contain the true values (Fig. 6a, b). Because the composite score used here weights only the LTR-RT divergence distribution and genome size, LTR-RT copy number and cumulative LTR-RT length were not used for fitting and therefore serve as held-out features. The best-fit simulation recovered the divergence distribution of the true genome and matched the two held-out features to within 4.8% and 3.2% errors, respectively, and the genome size to within 1.8% error (Fig. 6c, d). The solo LTR formation rate and the deletion length bias were not recovered, and the best 5% of simulations spanned nearly the entire searched range for each (Fig. 6e). This is reflected in the sensitivity analysis, in which insertion and deletion rate carry almost all the explanatory power of the fit (*R*^2^ = 0.59 and 0.53 on their own, respectively, and 0.94 for the four parameters together) while the solo LTR formation rate and the deletion length bias carry none (Fig. 6f). Our recoverability test shows that on a genome generated under a known history, PrinTE’s Grid Search Method accurately recovers the parameters and that the search independently identified the true insertion and deletion rates, showcasing PrinTE’s ability to accurately identify a quantitative hypothesis that recapitulate evolutionary histories (Fig. 6).

We next aimed to demonstrate the utility of PrinTE in modeling genome size evolution driven by TE bloat and purge on a real plant genome. We simulated a present-day *Arabidopsis thaliana* genome based on the reconstructed most recent common ancestor (MRCA) of the four *Arabidopsis* species analyzed, which had an inferred genome size of 168 Mb (Supplementary Fig. 11). We used the Grid Search Method to identify the LTR-RT conditions most consistent with *Arabidopsis* evolution under PrinTE’s model. Our analysis suggested that LTR-RT deletions exceeded insertions sixfold over the previous 5.1 million generations of evolution, with effective insertion and deletion rates of 1.0 × 10^–14^ and 6.0 × 10^–14^ per bp per generation, respectively (Supplementary Fig. 11).

**Fig. 7:**
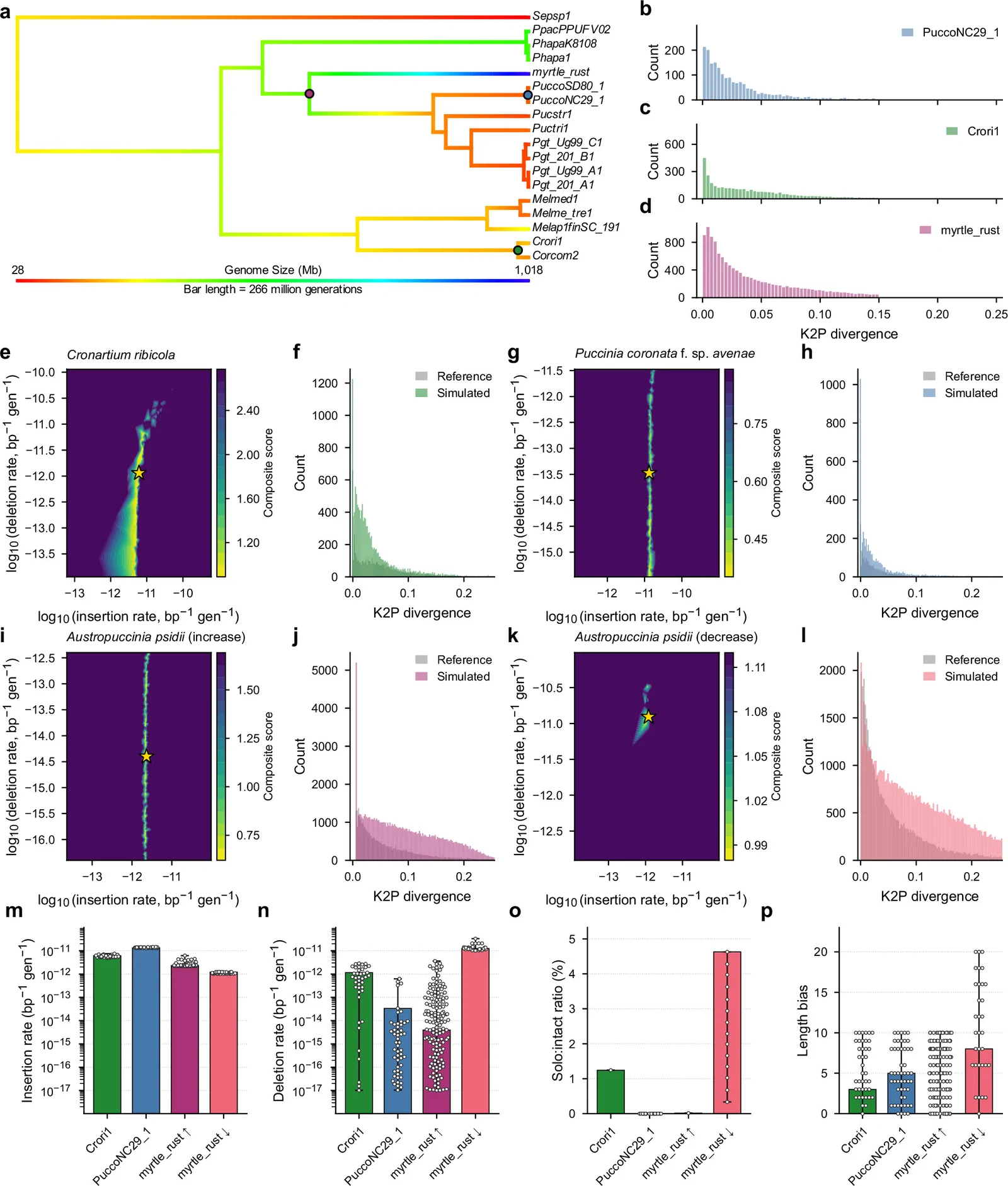
Inferring LTR-RT turnover dynamics and simulating genome size evolution across the *Pucciniomycetes*. **a** Time-scaled phylogeny of 18 rust fungi (*Pucciniomycetes*) with branches colored by genome size (28–1018 Mb) and the branch lengths scaled to 266 million generations of evolution. Filled circles highlight the ancestral simulation of the three focal genomes, colored to match **b**–**d.** The ancestrally reconstructed LTR-RT divergence distributions of (**b**) *Puccinia coronata* f. sp. *avenae* (PuccoNC29_1; blue), **c** *Cronartium ribicola* (Crori1; green), and **d** *Austropuccinia psidii* (myrtle_rust; magenta). **e**, **g**, **i**, **k** Composite-score landscapes from PrinTE parameter sweeps, projected onto the LTR-RT insertion- and deletion-rate plane (log_10_, bp^-1^ gen^-1^). Lower composite scores indicate closer agreement between the simulated and empirical genome, and the gold star marks the best-fitting parameter combination. **f**, **h**, **j**, **l** Sequence divergence of the best-fit simulated genome (colored) overlaid on the empirical reference distribution (gray). The reference is the present-day genome in every case except the *A. psidii* expansion phase (**j**), where it is the reconstructed LTR-RT divergence distribution at the start of the contraction phase. Panels are paired by genome with (**e**, **f**) *C. ribicola*, **g**, **h** *P. coronata f. sp. avenae*, **i**, **j** *A. psidii* during the expansion phase (genome size increase), and **k**, **l** *A. psidii* during the contraction phase (genome size decrease). **m–p** Best-fit PrinTE simulation parameters and summary statistics across the four scenarios (Crori1, PuccoNC29_1, myrtle_rust expansion (↑), and myrtle_rust contraction (↓)). **m** per-generation LTR-RT insertion rate, **n** per-generation deletion rate, **o** the solo:intact ratio of the simulated genome, which was not used as a fitting criterion, and **p** the deletion length bias parameter governing the probability of deletion based on TE length. Bars show the single best-fit value (the starred combination in **e, g**, **i**, **k**), and whiskers span the range (minimum to maximum) across the best 5% of simulations (*n* = 40, 52, 149, and 32 for Crori1, PuccoNC29_1, myrtle_rust ↑, and myrtle_rust ↓). Source data are provided as a Source Data file.

Despite their shared ancestry (Fig. 4a), the three selected fungal species (*C. ribicola*, *P. coronata* f. sp. *avenae*, and *A. psidii*) show immense variation in genome size and LTR-RT evolution. To test our hypothesis that *C. ribicola* has rapid but balanced LTR-RT insertion and deletion, we used PrinTE to reconstruct the genome size and LTR-RT divergence state of its MRCA from *C. comandrae* (Corcom2; Fig. 7a, c). Our inference suggests that *C. ribicola* diverged from the MRCA 5.4 million generations ago (Fig. 7a) with an estimated ancestral genome size of 180 Mb. We built a digital replica of the ancestor and forward-evolved it for 5.4 million generations to the present day using PrinTE. We simulated genomes with LTR-RT insertion and deletion rates spanning eight orders of magnitude to search for an LTR-RT divergence distribution that closely matches the observed distribution. In each simulation, we compared the simulated genome to the real genome using the Composite Distance Metric that evaluated the LTR-RT divergence distribution and genome size (Fig. 7e). Then, we used the best 5% of simulations to bound each parameter (these bounds are the intervals reported below and the whiskers in Fig. 7m–p).

After simulating 800 parameter combinations in *C. ribicola*, we found that an LTR-RT insertion rate of 6.1 × 10^–12^ per bp per generation (best-5% range: [4.9 × 10^–12^, 7.5 × 10^–12^]) and deletion rate of 1.1 × 10^–12^ per bp per generation (best-5% range: [1.0 × 10^–17^, 2.8 × 10^–12^]) produces a genome that closely matched the LTR divergence distribution of the *C. ribicola* genome, with the genome size increasing moderately through time (Fig. 7e, f, Supplementary Fig. 12a). However, the best 5% of simulations used a deletion rate that varied several orders of magnitude (Fig. 7n), suggesting the removal dynamics are a complex interplay between deletion rate, deletion length bias, and solo LTR formation rate (Supplementary Figs. 2–4). In the best-fit simulation, 35% of LTR-RT deletions produced a solo LTR. Simulation of the *C. ribicola* genome suggests that the species might have inserted one effective LTR-RT every 778 generations and deleted an LTR-RT roughly every 4134 generations during the last 5.4 million generations.

We repeated the same simulation process using the reconstructed ancestor of *P. coronata* f. sp. *avenae* 12NC29 (PuccoNC29_1) to test our hypothesis that the species has slow TE insertion and slow removal. We estimated that the PuccoNC29_1 strain diverged from its MRCA with the 12SD80 strain (PuccoSD80_1) 260,000 generations ago, with an ancestral genome size of 109 Mb (Fig. 7a). After simulating 1000 parameter combinations, we identified an LTR-RT insertion rate of 1.3 × 10^–11^ per bp per generation (best-5% range: [1.3 × 10^–11^, 1.5 × 10^–11^]) and a deletion rate of 3.4 × 10^–14^ per bp per generation (best-5% range: [1.0 × 10^–17^, 6 × 10^–13^]) that reproduced the LTR-RT divergence distribution of the real genome (Fig. 7b, g, h). In the best-fit simulation, 40% of LTR-RT deletions produced a solo LTR. With these rates, we see a gradual increase in average genome size, adding approximately 6.5 Mb per million generations (Supplementary Fig. 12b). These results suggest that, in contrast to *C. ribicola, P. coronata* f. sp. *avenae* appears to insert LTR-RTs with almost no compensating removal, with an average of about 700 generations between each effective LTR-RT insertion but only a single effective deletion across the entire 260,000-generation interval.

In these simulations, we observed a complex interplay between deletion rate, deletion length bias (“--k”), and the solo LTR formation rate (“--solo_rate”). A higher k value will lead to slower genome size increase due to the preference for deleting longer TEs (Supplementary Fig. 2). A higher solo LTR formation rate will lead to a higher solo:intact ratio and faster genome size increase, because solo LTRs are retained where the element would otherwise be removed entirely (Supplementary Figs. 3, 4). These interactions leave the deletion-related parameters only loosely constrained (Fig. 7e, g, i, Supplementary Figs. 3, 4), because a reduction in one can be compensated by the others, revealing the complexity of TE purging. Because of the interplay between deletion parameters, our hypothesis that *C. ribicola* purges LTR-RTs faster than *P. coronata* is only supported in a subset of our estimated parameter combinations (Fig. 7n).

Finally, we attempted to simulate the evolutionary trajectory of myrtle rust (*A. psidii*). Based on the genome size and LTR-RT divergence distribution (Fig. 4a, d), we expected that TE insertions had exceeded deletions for a long period of time, leading to genome size increase, followed by a shorter period of rapid LTR-RT deletions. Using a substitution rate of 2.0 × 10^–9^ per bp per generation^42^, we estimated that the contraction phase of *A. psidii* has lasted for approximately 1.4 million generations. We used PrinTE to create an initial burn-in genome of 244 Mb that mimicked the genome size and TE landscape of the reconstructed ancestral genome of *A. psidii* (Fig. 7d). Because *Austropuccinia* is a monotypic genus, the nearest available reference point is its MRCA with *Puccinia*, 114 million generations ago (Fig. 7a). The simulation was broken down into the LTR-RT expansion phase of 113 million generations followed by a contraction phase of 1.4 million generations, estimated based on the LTR-RT divergence distribution (Fig. 4d). For the expansion phase simulation, we aimed to obtain a genome size that was marginally larger than the real genome so the subsequent contraction phase could reduce it to the present-day genome size (Fig. 4d). Through evaluation of 2976 parameter combinations, we found that an insertion rate of 2.3 × 10^–12^ per bp per generation (best-5% range: [2.2 × 10^–12^, 2.6 × 10^–12^]) and a deletion rate of 4 × 10^–15^ per bp per generation (best-5% range: [1.0 × 10^–17^, 3.6 × 10^–12^]) could optimally model the ancestral LTR-RT divergence distribution at the end of the 113-million-generation expansion phase (Fig. 7i, j). At peak LTR-RT invasion, the simulated genome was 1082 Mb in size (Supplementary Fig. 12c).

To simulate the contraction phase, we took the output of the best-fit simulation from the expansion phase, then forward-simulated an additional 1.4 million generations. Systematic evaluation of 273 parameter combinations indicated that an insertion rate of 1.2 × 10^–12^ per bp per generation (best-5% range: [1.0 × 10^–12^, 1.2 × 10^–12^]) and a deletion rate of 1.2 × 10^–11^ per bp per generation (best-5% range: [1.0 × 10^–11^, 2.3 × 10^–11^]) produced a genome size that is near that of *A. psidii* (Supplementary Fig. 12d) and an LTR-RT divergence distribution that closely matches the real genome (Fig. 7l). In the best-fit *A. psidii* simulation, 80% of LTR-RT deletions produced a solo LTR.

To estimate myrtle rust TE evolution over the next one million generations, we used the empirically inferred TE evolutionary parameters to forward-evolve a digital replica of the present-day myrtle rust genome that was created using TEgenomeSimulator (Fig. 5). Our results suggest that the genome might contract from 1,018 Mb to 997 Mb, representing a 21 Mb reduction in size. In our simulation, genome downsizing is driven by a 10.5× preference in deletions over insertions, leading to 11,922 effective deletions and only 1,137 effective TE insertions over the simulated timeframe (Supplementary Fig. 13). The forward simulation represents a plausible trajectory given the empirically fit TE dynamics.

Simulation of *A. psidii* supports the two-phase hypothesis for the present-day genome size. The first phase was a long period of genome size increase in which LTR-RT insertions exceeded deletions by 582-fold, and the second was a short period where deletions exceeded insertions by roughly 10-fold. Compared to the expansion phase, the contraction phase has a ~ 50% lower insertion rate and >3000 times higher deletion rate (Fig. 7m, n). Our results support a recent contraction model for genome size evolution in *A. psidii* (Fig. 7i, k). It is important to note that the estimated parameters are effective insertion and deletion rates averaged from a long period of evolutionary time, which may not capture short episodic periods of TE bloat and purge, and that they are best read as the rates most compatible with the present-day genome under PrinTE’s model rather than as direct measurements of the historical rates.

Across all three lineages, the two opposing forces of LTR-RT insertion and deletion were sufficient to reproduce the LTR-RT divergence distribution of the present-day genome from its reconstructed ancestor (Fig. 7f, h, j, l), and the sensitivity analysis confirms that these two rates carry the fit in every case (Supplementary Fig. 14). Because each simulation was anchored to an independently inferred ancestral state and ranked against the real genome by a fixed Composite Distance Metric, the resulting quantitative hypothesis of LTR-RT dynamics is reproducible and free of manual tuning. Our results suggest that the amplification and degradation of LTR-RTs can vary widely in the *Pucciniomycetes* class within the fungal kingdom. Future studies may focus on the LTR-RT variability at narrower phylogenetic levels.

## Discussion

The TE composition profile of a genome serves as an evolutionary archive, preserving signatures of TE dynamics and host genome responses that collectively shape genome evolution. Deciphering these patterns from empirical data is challenging. Whether to test evolutionary hypotheses or to predict future trajectories, simulation is often necessary. To address this, we built PrinTE, a flexible, forward simulation framework for transposon evolution. PrinTE substantially expands the capacity for simulating complex TE dynamics and testing evolutionary hypotheses.

### PrinTE allows forward simulation of TE dynamics

Transposable elements are a major genomic component and subject to all evolutionary forces, including purifying selection, drift, and mutation. The ability to trace how TEs respond to these forces will greatly facilitate the study of TE dynamics. Forward simulation is a key approach that allows the artificial evolution of TEs and other genomic features, which provides opportunities to sample the intermediate states of the evolutionary process.

SLiM is a powerful forward simulation program that can simulate the majority of evolutionary scenarios^24^. However, its operation on single-nucleotide variants limits its application to TEs with varying lengths and abundant nesting. SLiM provides a workaround by first compressing the whole TE locus into an invariable presence and absence unit and performing simulations on these units. Sequence content is then filled back to replace the units to generate genomic sequences. This approach makes it difficult to model the variation accumulated through generations on the simulated TE sequences, including the deletion and formation of solo LTRs, which are critical for genome evolution studies. In contrast, PrinTE operates on whole-genome sequences, including invariant sites that allow intuitive modeling of complex scenarios and interpretation of results. In particular, PrinTE models the insertion and deletion rates of TEs and generates solo LTRs, which empowers the study of genome size evolution driven by TE bloat and purge.

The input files of PrinTE are highly flexible (Fig. 1). It has a built-in engine to simulate highly customizable starting genomes, in which users can specify genome size, TE content, TE superfamily abundance, and TE divergence in their simulations. PrinTE also takes simulated genomes from TEgenomeSimulator, allowing forward simulation from diverse backgrounds of genomes. Most importantly, PrinTE can take its own output as input (e.g., Fig. 3a-f), allowing for the simulation of varying TE dynamics and study of the host-TE arms race. This feature also enables the simulation of genomic divergence and speciation driven by differences in TE activities.

### TEs contribute to genome size expansion

The contribution of TE content to genome size expansion has been confirmed across many taxa and in our data (Fig. 4c). Over time, genomic data have been generated ever faster and at higher quality, and yet, as noted by Osmanski et al.^43^, the role of TEs in genome architecture and evolution is often an afterthought. In-depth analyses that yield a meaningful evolutionary perspective remain largely restricted to domain specialists.

To put forward mechanistic hypotheses, researchers have developed specialized computational methods to detect signals of TE radiation and impacts on speciation and genome size. For example, Ricci et al.^13^ developed a measure of TE Density of Insertion and Relative Rate of Speciation to model TE radiation bursts and silencing. Kapusta et al.^12^ estimated TE-driven DNA gains and losses and proposed the “accordion” model of TE radiation followed by DNA loss for tight genome size maintenance in mammals and birds. Osmanski et al.^43^ applied a Redundancy Analysis to study TE composition across mammalian taxa generated through the Zoonomia genomes^44^, and found evidence of multiple recent TE expansions driving larger genome sizes and widespread TE-silencing events across the mammalian tree. These authors hypothesize that variations in TE defense mechanisms may have played a role in whether specific lineages were overcome by a TE burst at a given evolutionary time.

However, we know little about how compact genomes are maintained despite evidence of TE radiation bursts, or about how large genomes are regulated. In the Chinese pine, TEs account for 69.4% of the 25.4 Gb genome, and LTR-RTs make up 86.5% of those TEs^16^. The Chinese pine genome showed no sign of recent whole-genome duplication, but 91.2% of its genes were undergoing discrete duplication or family expansion, strongly associated with TEs. Our results revealed two different genome dynamics in the Pucciniomycotina subdivision of fungi, including the *Microbotryomycetes* class that harbors compact genome sizes (18-50 Mb) and the *Pucciniomycetes* class with drastic differences in genome size (27-1,018 Mb) (Fig. 4a; Supplementary Data 2). The *Microbotryomycetes* class is almost devoid of LTR sequences (Supplementary Data 2), suggesting that the most effective way of maintaining compact genome sizes is to avoid the invasion of LTR-RTs. Genomes in the *Pucciniomycetes* class have varying richness of LTR-RTs (Supplementary Data 2), and the PCA reveals several types of LTR dynamics (Fig. 4b) that are likely major drivers of genome size variation.

The myrtle rust fungus expanded its genome size from a reconstructed ancestral size of 244 Mb to the current 1,018 Mb, experiencing a ~ 4× increase. Our simulation studies indicate a recent shift toward genome downsizing, with LTR-RT deletions being >3000 times faster than in the expansion phase (Fig. 7n). Using PrinTE’s Grid Search Method, we were able to simulate a genome that mimicked the TE landscape of the ancestral genome of myrtle rust, and further used PrinTE to simulate two phases of evolution, including a long expansion period and a short contraction period. This model fits well with the observed composition of the myrtle rust genome, making the two-phase scenario an effective quantitative hypothesis for its genome size history.

Because a fit is scored against present-day genome summaries, the parameters PrinTE returns are best read as the TE insertion, deletion, and solo LTR formation rates most compatible with an observed genome under PrinTE’s model. Different histories can leave similar present-day signatures, so a good fit is a scenario sufficient to produce the observed genome, but not the only scenario that could have produced it. Within that limit, the parameters that carry the inference are recoverable. In our recovery test using a genome generated under a known history, the search returned both the insertion and the deletion rate to within 6% of their true values from a space spanning four orders of magnitude. The solo LTR formation rate and the deletion length bias, which the sensitivity analysis ranks as weakly informative, were not recovered (Fig. 6). Where independent evidence exists, it agrees with the direction of our inferences. For *Arabidopsis thaliana*, our search inferred that LTR-RT deletions exceeded insertions sixfold over the last 5.1 million generations (Supplementary Fig. 11), consistent with comparative genomic evidence that the *A. thaliana* lineage has actively shed DNA relative to its close relatives^19,41^. The LTR-RT half-life assumed by PrinTE (~500,000 generations) is also consistent with the rapid LTR-RT turnover inferred from structural analyses of LTR-RTs in other eukaryotic genomes^32,33^, and the long expansion followed by rapid contraction that we infer for *A. psidii* mirrors the accordion-like alternation of DNA gain and loss reconstructed independently in birds and mammals^12^. Direct experimental measurements of transposition, such as mutation-accumulation lines, pedigree data, or the population-level insertion polymorphisms assembled for a small number of model species^8,27^, do not yet exist for the Pucciniomycotina, and would provide the external test of the rates reported here.

Finally, we used TEgenomeSimulator to smooth out small-scale TE activities, and in combination with PrinTE-estimated dynamics, we simulated the mobilization and deletion behavior of TEs to predict the dominant expansion or contraction events. We recognize the importance of accounting for more complex patterns in sequence divergence distributions, which may go beyond simple normal distributions. The combined use of PrinTE and TEgenomeSimulator greatly enhanced the simulation capacity, enabling richer and more flexible hypothesis testing across both evolutionary and mechanistic contexts. It is important to note that PrinTE is a single-genome, panmictic approximation designed to explore how TE parameters may shape genome composition. It does not model population-level forces such as drift, effective population size, mating system, admixture, polyploidy, or migration. Users may combine PrinTE with population genetic tools such as SLiM to facilitate population-level analyses.

PrinTE also models sequence-level decay rather than functional evolution. Elements accumulate substitutions and are truncated, nested, and fragmented, so an initially intact element can lose its coding capacity, but its mode of mobilization is fixed by its family assignment and does not switch during a simulation. The initial balance of autonomous and non-autonomous elements is set by the user through the TE library and superfamily ratios rather than emerging from the simulation. Across successive runs however, users can approximate this transition with PrinTE’s continuation functionality where a simulation can be paused, its TE library and superfamily ratios revised to introduce or expand non-autonomous derivatives, and the run continued from the same genome, so that a shift in library composition and transposition dynamics is imposed. Modeling the transition explicitly is still difficult since the boundary between autonomous and non-autonomous is not binary, with many members of a family retaining only a subset of coding domains, and the mapping from a given coding-sequence mutation to a change in mobilization behavior remains poorly characterized. Future development would benefit from incorporating features such as customizable divergence distributions, more functional genomic context, including repeat-induced point mutation in fungi^45^, epigenetic regulation mechanisms, and additional insertion-site preferences; and better support for quickly evolving TE features such as solo LTRs, which are currently difficult to detect sensitively.

In conclusion, PrinTE is a versatile tool that allows flexible forward simulation of whole-genome sequences with fine-grained user control of genome size, TE content, superfamily abundance, TE divergence profile, TE insertion, TE deletion, TE mutation, solo LTR formation, genome divergence, and speciation. We demonstrated the use of PrinTE in modeling the genome size evolution of Pucciniomycotina genomes. In particular, we inferred TE dynamic parameters consistent with the genome size history of myrtle rust (*Austropuccinia psidii*) and other large fungal genomes. Our work turns TE-driven genome size evolution into a traceable and explorable simulation problem, and provides a framework for testing quantitative, data-driven hypotheses.

## Methods

### Implementation of PrinTE

#### Burn-in

PrinTE simulates the forward evolution of genomes. The starting point for simulation is a burn-in genome in FASTA format and a corresponding PrinTE-formatted BED file. These will serve as the reference (generation 0) for which no forward evolution has yet taken place. The burn-in FASTA and BED can be generated de novo by PrinTE, or previous PrinTE outputs can be fed back into PrinTE for additional forward simulations. PrinTE de novo burn-in allows many customizations, including a wide range of genome size, chromosome number, percentages of the genome that are genic and TE sequences, customization of TE divergence, and the ratio of each TE superfamily in the genome. Each TE insertion creates a TSD with the length determined by the TE superfamily, which was adopted from the TEgenomeSimulator codebase. In some instances, it may be beneficial to apply baseline mutation to TEs in the burn-in such that they do not exactly match elements in the TE library. This is particularly relevant for self-dating elements, like LTR-RTs, where the default TE library only contains very young LTR-RTs, with exactly identical LTRs. For baseline mutations in the burn-in genome, we apply a normalized exponential decay distribution to skew mutations heavily toward lower mutation values:

1 $$f(M)=\frac{k}{{M}_{\max }\left(1-{e}^{-k}\right)}{e}^{-k\frac{M}{{M}_{\max }}}$$

The probability density function is aimed at flexibly and accurately modeling TE divergence distributions as they are observed in real data, where aggressive TE purging leads to higher probability of observing young TEs (Fig. 4d). The parameters *M*_*max*_ (maximum mutation percentage; “--TE_mut_Mmax”) and *k* (decay constant; “--TE_mut_k”) are customizable, allowing users to adjust the shape of the distribution to reflect a wide range of evolutionary and biological scenarios and to match the observed TE half-life of any species (see Supplementary Fig. 1a,b). The half-point of sequence divergence among the simulated TEs is:

2 $${M}_{1/2}=\frac{{M}_{\max }{{\mathrm{ln}}}\left(2\right)}{k}$$

#### Two simulation modules

Forward simulation begins once the burn-in genome is established. Users can specify the total number of generations (“--generation_end”) and a smaller number of generations for each iteration (“--step”). There are three sequential modules for forward simulation: (1) genome mutation, (2) TE insertion, and (3) TE deletion. The pipeline will iterate over these processes for each step until the total number of generations is simulated. PrinTE can iterate through modules using constant and fixed TE insertion and deletion rates, or it can iterate using self-adjusting variable TE insertion and deletion rates. The fixed and variable methods begin identically, starting with the mutation of the genome. The expected number of DNA mutations per sequence (λm) is calculated as:

3 $$\lambda m={DNA}\,{mutation}\,{rate}\times {genome}\,{size}\times {generations}$$

Because TE insertions can occur at any generation of a defined evolutionary window (*i.e*., a simulation step), they could accumulate DNA mutations less than λm. To capture this dynamic, we used a normalized exponential decay function similar to the decay function used for the burn-in genome to weight TE mutations toward lower values. We anchored the mutation distribution here to approximate the complicated evolutionary dynamics of TE activities. The actual number of mutations for each newly simulated TE is sampled from the probability density function, $f(m{TE})$, to help weight their observed mutations toward lower frequencies:

4 $$f\left({m}_{{{{\rm{TE}}}}}\right)=\frac{1.4\times {10}^{-6}\cdot {e}^{-1.4\times {10}^{-6}\cdot {m}_{{{{\rm{TE}}}}}}}{1-{e}^{-1.4\times {10}^{-6}\cdot 3,000,000}},0\le {m}_{{{{\rm{TE}}}}}\le 3,000,000$$

The half-life of simulated TE insertions is t_1/2_ = ln(2)/k, where k = 1.4 × 10^–6^, thus t_1/2_ ≈ 495,000 generations, capturing the observation of TE half-life reported in multiple eukaryotic organisms^32^.

### Simulation with fixed rates

#### TE insertion

After DNA mutations, TEs are inserted into the genome at random locations. They may insert into intergenic regions, into other TEs, resulting in nested TE insertions, or into genes, resulting in fragmented genes. Donor TEs for insertion are randomly selected from the TE library based on user-defined abundance ratios per superfamily. Information about nesting and fragmentation is recorded in the BED file. Families designated as cut-and-paste in the TE ratios table are excluded from replicative amplification and instead mobilize by excision. Each excision of an intact element creates a relocation debt for that family, which is repaid at the next simulation step by re-inserting elements of the same family. The expected number of re-insertions per excision is set by “--cutpaste_reinsertion” (default 1.0). Values below 1.0 model unsuccessful re-insertion and net copy-number loss, and values above 1.0 model replication- and repair-coupled amplification. For an excision of 10 elements, a value of 0.7 means 7 out of 10 excisions will be reinserted to the genome, causing a net loss of 3 elements; a value of 1.7 means 10/10 excisions will be reinserted and 7/10 have the original copy recovered, causing a net gain of 7 elements. Copies that are already fragmented, including those interrupted by a nested insertion, are lost without re-insertion. In the fixed-rate method, the number of TEs to be inserted (${\lambda }_{i-{fix}}$) is calculated as:

5 $${\lambda }_{i-{fix}}={TE}\,{insertion}\,{rate}\times {genome}\,{size}\times {generations}$$

#### TE deletion and solo LTR

A nested insertion will divide the acceptor TE into two fragments. For example, if TE1 inserts into TE2, it will generate three sequences for further operations (e.g., deletion), including two resulting fragments of TE2 and the complete sequence of TE1. This differs from simple insertions into intergenic regions, where each insertion results in only one sequence for further operations. During the deletion process, full-length TEs and TE fragments are all eligible for deletion. In nested insertions, if an acceptor TE’s fragment is selected for deletion, only the fragment is deleted with other fragments of the TE remaining in the genome. The number of TE deletions (${\lambda }_{d-{fix}}$) is calculated as follows:

6 $${\lambda }_{d-{fix}}={TE}\,{deletion}\,{rate}\times {genome}\,{size}\times {generations}$$

Intra-element homologous recombination is a prevalent process for TE deletions and is facilitated by sequence similarity between nearby homologous sequences^33^. Longer sequences have a higher probability of initiating homologous recombination because they contain more similar base pairs to initiate pairing; consequently, longer TEs are more likely to be deleted than shorter TEs. We used an exponential decay function to model an exponentially increasing probability of TE deletion as TE length increases:

7 $$f(L)={e}^{-k\cdot \left(1-\frac{L}{{L}_{95}}\right)}$$

Here, *L*_*95*_ represents the TE length at the 95^th^ percentile of the length distribution in the genome, so long sequences will be ignored. *L* represents the length of a TE (whether intact or fragmented), and k is a user-defined parameter (“--k”) that controls the rate at which deletion probability increases with sequence length. Higher k biases deletions toward longer TE features by assigning higher deletion weights to longer sequences (Supplementary Fig. 1d). Except for LTR-RTs, TE deletions remove the entire TE feature. If a nested TE is selected for deletion, the adjacent TE fragments it disrupts are reconstituted into an intact TE. LTR-RTs are an exception to full-length deletions because they can undergo recombination with themselves owing to the sequence homology between their long terminal repeats, resulting in the formation of a solo LTR. PrinTE supports deletions that lead to solo LTR formation. If an LTR-RT is selected for deletion, it will be converted to a solo LTR at a user-specified solo LTR formation rate, which is set by default to 95% (“--solo_rate”).

### Simulation with variable rates

In addition to the fixed-rate TE insertion/deletion method outlined above, in which the number of insertions and deletions per generation is constant and modeled by user-selected rates, PrinTE also offers a variable method that self-adjusts the number of insertions and deletions per generation based on genomic landscapes. This aims to replicate a subset of the biological forces that affect TE insertions and deletions. The three core processes, (1) genome mutation, (2) TE insertion, and (3) TE deletion, are shared between methods, and the genome mutation step (described above) is identical in both.

#### TE insertions

In real genomes, the rate of TE transposition is influenced by the number of intact TEs, since only intact TEs are capable of initiating new insertions. Genomes with a high number of intact TEs tend to experience more transpositions per generation than those with few. Fragmented TEs and solo LTRs are no longer viable for transposition. In the variable method, the number of TE insertions (${\lambda }_{i-{{\mathrm{var}}}}$) is modeled as follows:

8 $${\lambda }_{i-{{\mathrm{var}}}}={TE}\,{insertion}\,{rate}\times {number}\,{of}\,{intact}\,{TEs}\times {generations}$$

This simulates insertions via transposition, but some TEs will be acquired horizontally and drift into the genome. We specify that the frequency of horizontal TE acquisition is influenced by TE prevalence within the species: species with TE-rich genomes are more likely to acquire additional TEs horizontally, while species with TE-poor genomes are less likely to do so. For this calculation, the TE composition of the genome is surveyed before simulations and used as a proxy for TE abundance in the species. Horizontally acquired TEs (${\lambda }_{i-{hor}}$) are then simulated accordingly:

9 $${\lambda }_{i-{hor}}={TE}\,{horizontal}\,{rate}\times {TE}\,{composition}\,{of}\,{the}\,{species}\times {generations}$$

#### Insertion bias

The distribution of TEs across chromosomes is uneven and can be influenced by chromatin states, with euchromatin generally more accessible for TE insertion. In the variable method, we model this by treating genes and their surrounding regions as euchromatin, and the gene-poor genomic backbone as heterochromatin. We can modulate insertion location probability by chromatin type using a bias factor (“--chromatin_bias_insert”). We assign insertion probability weights to euchromatic and heterochromatic sequences as follows:

10 $${Euchromatin\; weight}={length}\times {bias\; factor;Heterochromatin\; weight}={length}$$

It is important to note that PrinTE does not use genes as functional units, but rather as representative segments of the genome to which cellular and selective attributes can be assigned. This enables the modeling of global genome features. Unlike the fixed-rate method, where TEs are inserted at random in proportions defined by the user, the variable-rate method inserts TEs in proportions that reflect the current genomic composition.

#### Fitness-dependent TE deletions

TEs can trigger extensive and rapid proliferation, resulting in a sudden increase in their genomic abundance. If left unchecked, this proliferation can destabilize the genome and impose a selective disadvantage on the host organism. Under the variable-rate method, the number of TE deletions is dynamically adjusted according to the extent of gene disruption caused by TE insertions. In this context, genes are modeled as sequences subject to purifying selection, whereby insertions of TEs into these regions are deleterious and thus reduce the observed number of TE insertions. PrinTE also supports designating the range of upstream sequence of the gene as promoter sequence such that the nearby gene is considered functionally disrupted if a TE inserts into its upstream promoter sequence (“--promoter-boundary”). The number of TE deletions (${\lambda }_{d-{{\mathrm{var}}}}$) is then determined by the following expression:

11 $${\lambda }_{(d-{{\mathrm{var}}})}={TE\; deletion\; rate}\times \sum ({disrupted\; gene\; weights})\times {generations}$$

In actual genomes, not all genes that confer a selective advantage exert the same influence on fitness. While most TE insertions into genes are deleterious, some have more severe effects. To capture this variability, we model the distribution of TE selection pressures across genes using a log-normal distribution (Supplementary Fig. 1c):

12 $$f(x;\sigma )=\frac{1}{x\sigma \sqrt{2\pi }}{e}^{-\frac{{({{\mathrm{ln}}}x-{\sigma }^{2})}^{2}}{2{\sigma }^{2}}},x > 0$$

In this framework, each disrupted gene contributes a positive value to the total disrupted gene weight. The mode of the distribution is fixed at 1 to gain better control of the spread. Because of the long tail of the distribution, a small subset of genes exerts a disproportionately large influence on fitness. Consequently, TE insertions in these regions lead to a substantial increase in deletion events. The parameter σ, which is set via the command line (“--sigma”), determines the spread of the distribution. Larger values of σ produce longer tails and increase the range of selective effects among genes. In reality, TE insertions in some genomic regions can be lethal. PrinTE does not simulate lethality explicitly. Instead, we assume that simulating many generations using a stepwise approach effectively filters out lethal insertions. Over time, such deleterious types are eliminated from the population and do not persist in the simulated genomic landscape.

#### TE deletion bias

Once the number of deletions is calculated, the specific TEs to delete are determined by the same exponential decay function (f(L)) used in the fixed-rate method, multiplied by additional genomic attributes that might affect the probability of TE deletion. These genomic attributes include the selection coefficient parameter (“--sel_coeff”) and the chromatin bias parameter (“--chromatin_bias_delete”). The selection coefficient parameter is used to assign additional deletion weight toward the TEs that disrupt genes. Similarly, we can assign additional weight toward deletions in simulated euchromatin regions. Longer TEs and TEs nearer to or within impacted genes are more likely to be deleted based on user-specified thresholds.

#### LTR-RT dating

PrinTE implements an accurate approach called Kmer2LTR (Fig. 2a, b) to estimate sequence divergence between the LTR regions of LTR-RTs. Optionally, Kmer2LTR uses known LTR region boundaries as input to initiate the LTR-RT dating process. For empirical data where the location of LTRs is unknown, we developed a k-mer approach to estimate LTR region boundaries. The k-mer approach first uses Jellyfish^46^ to identify and count k-mers of 8-12 bp. K-mers occurring exactly twice in the LTR-RT are mapped back to the LTR-RT. The resulting k-mer pairs are sorted numerically by position. Pairs that disrupt the monotonic order or deviate by more than two standard deviations from the mean distribution of map position are removed. The map coordinates of the remaining k-mer pairs are used to establish the putative LTR region. A 65 bp extension is applied to each of the putative k-mer-determined LTR regions to ensure that the LTRs are fully captured. MAFFT^47^ is used to align LTR regions within each LTR-RT, and trimAl^47,48^ is used to prune overhanging extensions and to remove small gaps. This approach accurately identifies the boundaries of LTR regions from young to very old LTR-RTs. The gap-free and extension-cleaned LTRs are then fed to the wavefront alignment (WFA) algorithm^47–49^ for global sequence alignment with a two-piece gap-affine scoring model to estimate sequence divergence. With these implementations, Kmer2LTR only requires a FASTA file of LTR-RTs and identifies the LTR regions de novo. Kmer2LTR may drop highly diverged and ancient LTR-RTs with LTR divergences ≥35%. In practice, this is infrequently an issue because LTR-RT detection methods rarely detect such highly mutated LTR-RTs, and those that are detected may be enriched for false positives. This pipeline is freely available on GitHub (https://github.com/cwb14/Kmer2LTR).

### Searching for TE dynamic parameters using PrinTE

To identify optimal TE dynamics in a given genome, we implemented a systematic parameter sweep approach using PrinTE. This Grid Search Method involved iteratively testing combinations of key parameters to approximate empirical TE behavior and genome size observed in the genome of interest.

#### Parameter space exploration

The Grid Search Method iterated over four parameters that control forward TE evolution in PrinTE: (1) the per-generation TE insertion rate, logarithmically sampled, (2) the per-generation TE deletion rate, logarithmically sampled, (3) the solo LTR formation rate, numerically sampled, and (4) the deletion length bias parameter that controls the length preference for deleting TEs, numerically sampled. Each combination of these four parameters is used to run a PrinTE forward simulation starting from a fixed burn-in genome mirroring the inferred ancestral state. Because both the ancestral state (inferred by ancestral state reconstruction) and the present-day reference are independently quantifiable, sweeping many such past-to-present simulations across the four-dimensional parameter space provides the statistical power to identify TE dynamics consistent with the observed LTR-RT divergence distribution. To effectively manage simulations, we implemented two parameters to control for simulated genome size. The simulated genome size is set to ±10% of the present-day reference using the maximum (“--mxgs”) and minimum (“--mngs”) genome size parameters, which together define the genome size window. The genome size of each simulation step is checked, and those that fall outside of this expected genome size are logged and terminated early to save computation.

The parameter sweep involves multiple rounds of search. The first round of the search draws parameter combinations from the four-dimensional space by Latin hypercube sampling^50^, an approach that produces well-distributed coverage of all four parameters simultaneously even with relatively few samples. Insertion and deletion rates are sampled log-uniformly across user-specified bounds spanning several orders of magnitude per bp per generation. The solo LTR formation rate is sampled from integer values between user-specified lower and upper bounds in user-defined steps, and the deletion length bias parameter is sampled from integers across its allowed range. Each combination is launched as an independent PrinTE simulation in parallel, with support for SLURM-style parallel computing and local use with the available CPU threads partitioned across concurrent simulations.

Because the four-dimensional space is often too large for comprehensive gridding, after the first round (“--samples” parameter, default 200), the search shifts from blind sampling to a guided strategy driven by a surrogate model. After each round, completed results from every simulation are assembled into a training table relating the four input parameters (log-transformed for the insertion and deletion rate parameters) to the resulting genome size at the end of the simulation. A Random Forest regression model is fit to this table and used to propose the next round of candidates from a large pool of random draws. Candidates are scored by how close the model predicts their final genome size will be to the midpoint of the genome size window, and by how uncertain the model is about that prediction. A weighting term, α, on the uncertainty allows the search to favor exploration of poorly understood regions of parameter space early on and is decayed by a constant factor each round so that later rounds increasingly favor exploitation of the most promising regions.

To prevent the model from prematurely collapsing onto a narrow region, a user-specified fraction of each round’s candidates (“--explore-frac” parameter, default 0.1) is drawn at random rather than from the top of the score list. To make full use of every simulation, runs that did not reach the terminal generation but produced a reliable projection of their final genome size are also included in training, weighted by the coefficient of determination (R^2^) of that projection to minimize the contribution of poorly projected runs. Simulations whose final genome size falls inside the genome size window are recorded as hits, and the loop runs until a user-specified number of hits (“--target-hits” parameter, default 200) has been accumulated, with a hard cap on the maximum number of rounds (“--max-rounds” parameter, default 100). All intermediate states are written to disk so that the search is fully resumable from interruptions. For each completed simulation, intact LTR-RTs are annotated de novo from the final FASTA file using a custom LTR-RT annotation pipeline.

To rank the parameter combinations by how closely each simulated genome matches the empirical reference, we compute a Composite Distance Metric per simulation. This metric combines four normalized, dimensionless components: (1) the relative difference in the number of LTR-RTs between the simulated and reference genomes, (2) the relative difference in their cumulative LTR-RT length, (3) a root-mean-square of the bin-wise density difference between their LTR divergence distributions, and (4) the relative difference from the reference genome size. The LTR-RT divergence distributional distance is computed as the root-mean-square of bin-wise density differences across 50 equal-width bins spanning the joint p-distance range of the two genomes. The four components are combined as a weighted sum, with user-specified weights (“--alphas” parameter, default 0, 0, 10, 1) that allow flexible prioritization of which features of the empirical TE landscape should dominate the ranking. Parameter combinations achieving the lowest composite scores are taken as the closest simulated approximations to the empirical TE dynamics of the reference genome.

#### Parameter recovery test

To test whether the Grid Search Method recovers known parameters, we generated a test genome with PrinTE under a known parameter combination and then attempted to recover those parameters without reference to them. A 100 Mb burn-in genome (four chromosomes, 25% of the genome are genic sequence, 21% of the genome are intact TEs, and an LTR-RT-only TE library) was forward-evolved for three million generations with a step size of 500,000 generations using “-F 3e-11,1e-11” together with PrinTE’s default solo LTR formation rate (“--solo_rate 95”) and default deletion length bias (“--k 10”), which expanded the genome from 100.0 Mb to 127.6 Mb. The resulting genome was then treated as an empirical reference. Its intact LTR-RTs were annotated de novo as if it were a real genome, and the Grid Search Method was run from the generation-0 burn-in over the same four parameters searched for the rust fungal genomes (“--ins-start 1e-10 --ins-end 1e-14 --del-start 1e-10 --del-end 1e-14 --sr-start 5 --sr-end 95 --sr-step 5 --k-start 0 --k-end 10 --k-step 1”), a substitution rate of 1.3 × 10^-8^ per bp per generation, a genome size window of ±5% of the reference (“--mxgs 133943605 --mngs 121187073”), and the default weights (“--alphas 0 0 10 1”). Four rounds of search evaluated 650 parameter combinations, of which 325 produced a scored terminal genome. The rest left the genome size window and were terminated early. Recovered parameters and genome summaries were compared with those of the true genome, with the reported interval taken from the best 5% of simulations (*n* = 33), as in the empirical analyses.

#### High-throughput simulation framework

PrinTE simulations were executed on an HPC cluster using array jobs (SLURM workload manager), enabling parallel processing of parameter sets. Users can set the total number of generations (“-ge”) with sampling steps (“-st”) for all simulations during parameter sweeps.

The species-specific substitution rate (“--mutation_rate”) is also critical to the simulation outcome. In cases where an accurate substitution rate is not available for the species, users can try rates in different magnitudes (e.g., 1 × 10^–8^, 1 × 10^–9^, or 1 × 10^–10^ per bp per generation) in the simulation, then compare the LTR divergence of simulated genomes to the real genome. Higher substitution rates will introduce more mutations to TEs in a given timeframe, thus creating gentler slopes of LTR divergence. Conversely, lower substitution rates will create steeper slopes of LTR divergence.

#### Empirical estimation of LTR divergence and solo:intact ratio

Outputs were processed through a standardized pipeline to assess TE dynamics, including genome size change, LTR divergence distributions, and solo:intact ratio. Intact LTR-RTs were extracted from each simulation using the “extract_intact_LTR.py” script; their divergences were estimated with the K2P model and a mutation rate of 2.0 × 10^–9^ per bp per generation using the “seq_divergence.py” script, and divergences were plotted using the “ltr_dens.py” script, all available in PrinTE.

Although the BED file output of PrinTE contains the annotation of all intact and solo LTR sequences, we performed a de novo scan of intact LTR-RTs using LTR_retriever (v3.0.1)^32^ on the simulated genomes, so that these results are comparable to those obtained from the real genome. The resulting LTR library was used to annotate whole-genome LTR sequences with RepeatMasker (v4.1.7-p1)^51^. Solo LTRs were then identified by the “solo_intact_ratio_wrap.sh” script available in the LTR_retriever package, which also calculated the overall solo:intact ratio of each genome. For the same reason, LTR divergence distributions of simulated genomes were also plotted using intact LTR-RTs identified by LTR_retriever. The “plot_divergence.R” script available in PrinTE was used to plot the density of LTR divergence in simulated and real genomes. Parameter sets were ranked by the Composite Distance Metric described above. With the weights used here (“--alphas 0 0 10 1”), the ranking is governed by the genome-wide LTR divergence distribution and, secondarily, by genome size. Solo:intact ratios were computed for description and comparison but were not used as a fitting criterion because this metric is sensitive to the completeness of solo LTR annotation. This approach identified optimal parameters where simulated TE dynamics showed convergence with multiple empirical metrics while maintaining computational feasibility.

### Benchmarking and performance testing

For benchmarking purposes, we disabled the probability density function that controls the number of LTR-RT mutations in the burn-in ($f(m{TE})$) so that LTR-RTs had mutation rates mirroring those of the genome. This allowed us to compare with other simulators that do not model such phenomena. We used PrinTE with parameters “-P 10 -itn 150 -cn 1 -sz 100 Mb -ge 8500000 -st 500,000 -t 20 -k 0 -kt -m 3e–8 -sr 0 -tmx 0 --TE_lib ./PrinTE/athrep.ltr.full.gz”. We used low rates of TE insertion and deletion (“-F 3.0e-200,5e-200”) to effectively disable transpositions and purging so that the genome and its LTR-RTs are allowed to mutate with only DNA substitutions. Three replicates with “-TiTv 10” and “-TiTv 1” allowed us to compare the performance of PrinTE’s models of evolution. For comparison, we used SLiM to evolve a single 100 Mb diploid lineage (N = 1). SLiM does not implement an iterative stepwise approach and instead models each generation. To reduce runtime, we used SLiM to model ancestral trees with no mutations (MU = 0), then projected DNA substitutions onto the mutation-free SLiM genealogies at half-million generation steps from 0 to 7.5 million generations using a mutation rate of 3×10^-8^ per bp per generation and Ti/Tv ratios of 1 or 10. Mutations in SLiM-simulated genomes were detected by whole-genome alignments using Minimap2 with flags “-cx asm20 --cs=long --secondary=no”. The PAF alignment files were provided to “paftools.js” from the Minimap2 package with the “stat” command to calculate p-distance from the reported number of substitutions and alignment length. Similarly, K2P divergence was calculated using the cs tag in the PAF file by classifying substitutions as either transitions (purine to purine or pyrimidine to pyrimidine) or transversions (purine ↔ pyrimidine).

LTR-RT redetection was compared among SimulaTE, ReplicaTE, and PrinTE simulations. SimulaTE requires a chassis genome as input. PrinTE was used to build the chassis using “--burnin_only --cds_percent 0 --intact_TE_percent 0 --chr_number 1 --size 1 Gb”. The “define-landscape_random-insertions-freq-range.py” from SimulaTE was run using “--N 3 --insert-count 2000 --min-distance 1000 --min-freq 0.1 --max-freq 0.9”. The resulting pgd file was provided to “build-population-genome.py” from SimulaTE to create the final TE-infused genome. ReplicaTE was used to insert Ty1 and Ty3 elements from *Drosophila melanogaster* chromosome 2 using the GenBank file from the package. PrinTE was run with parameters “--burnin_only --cds_percent 0 --intact_TE_num 1000 --chr_number 1 --size 500 Mb -s 21 --threads 200”. Three replicates were used for each tool. LTR-RT redetection was conducted using LTR_FINDER_parallel and LTR_HARVEST_parallel with parameters “-w 2 -C -D 15000 -d 100 -L 7000 -l 60 -itp 20 -M 0.85 -S 3” and “-minlenltr 60 -maxlenltr 7000 -mintsd 4 -maxtsd 8 -mindistltr 300 -maxdistltr 20000 -similar 85 -vic 10 -seed 20 -seqids yes -xdrop 6”, respectively. The resulting LTR-RT candidates were provided to LTR_retriever with “-motif [TCCA TGCT TACA TACT TGGA TATA TGTA TGCA TGAA AGTT AGCT]” to identify LTR-RTs.

PrinTE runtime and peak memory were assessed on an HPC server with 256 CPU threads of AMD EPYC 7763 at 2.45 GHz and 1024 GB DIMM DDR4 memory at 3200 MHz. Metrics were collected from the report generated using “/usr/bin/time -v”. PrinTE was run using “-cn 20 -sz 100 Mb -P 20” to simulate a 100 Mb genome with 20 chromosomes in which 20% of the genome is genes. “-itn 6000” was used to simulate 6,000 initial TE insertions. “-ge 1000000 -st 100000” simulated 1,000,000 generations of TE evolution with step sizes of 100,000 generations (10 iterations total). Insertion and deletion rates, “-F 3.0e-11,5e-11”, were used to insert and delete ~500 TEs per iteration.

### Syntenic ribbon plot and LTR-RT phylogeny

To simulate genomes presented in Fig. 3a, 19,621 *Arabidopsis thaliana* coding sequences (CDS) were inserted into the simulated genome using PrinTE with “--cds_num 19621” to guide collinear anchoring and enable syntenic analysis. The burn-in genome consisted of 80% intact LTR-RTs (“-itp 80”). The genome contraction phase was run using “-ge 5,000,000 -st 1,000,000 -F 1e–11,4e–11 -dg -k 3”. The genome size increase phase was run using identical parameters except “-F 4e–11,1e–11 --continue” and the contraction phase outputs as the input. The simulated genomes were processed pairwise using “jcvi.compara.catalog ortholog --cscore = .99” in JCVI (v1.5.4)^52^. Syntenic anchors were visualized using “matplotlib.pyplot” in Matplotlib (v3.10.3) to create the ribbon plot (Fig. 3a). The LTR-RT phylogeny (Fig. 3d, e) was created using only elements in the *Tekay* family, which were classified as such by TEsorter (v1.4.7)^53^. *Tekay* LTR-RT headers were tagged with “_shared” and “_uniq” to facilitate post-PrinTE parsing. Shared LTR-RTs were inserted using PrinTE with parameters “-itp 80 -cn 1 -sz 10 Mb -tmx 10 --TE_lib Tekay_shared.fa -tk 15”. The outputs (“burnin.fa” and “burnin.bed”) were duplicated and allowed to continue simulation using either genome expansion parameters (“--generation_end 5,000,000 --step 500,000 -F 2e-10,1e-10 --TE_lib Tekay_uniq.fa”) or genome contraction parameters (“--generation_end 5000000 --step 500,000 -F 2e-11,1e-11 --TE_lib Tekay_uniq.fa”). Intact LTR-RTs were extracted using “extract_intact_LTR.py” and classified using TEsorter with “-db rexdb-plant -dp2”. Reverse transcriptase domains identified from *Tekay* LTR-RTs were aligned using MAFFT (v7.525)^54^ and provided to VeryFastTree (v4.0.5)^55^ for phylogenetic analysis. The resulting newick trees were plotted using ggtree (v3.1.5)^56^ with branches colored by their ancestral relationship. The *Arabidopsis thaliana* simulation used to generate Fig. 3g-j was created using PrinTE with parameters “-cn 5 -sz 135 Mb -tmx 5 -m 7e-9 -P 25 -itp 21 -br 1e–7 -ir 1.1e–6 -dr 4e–6 -cbi 1.1 -cbd 1.0 -cb 500 --k 2 -ge 1000000 -st 10000 -c TAIR10.cds.fa”.

### Pucciniomycotina genomes and annotations

The myrtle rust (*Austropuccinia psidii*) genome was downloaded from NCBI with accession ID ERS3953144^3^. Genomes of the Pucciniomycotina fungal subdivision were obtained from the JGI Fungal Genomics Resource MycoCosm (https://mycocosm.jgi.doe.gov/) (Supplementary Data 1). Rust fungal genomes with contig N50 values higher than 100 kb were used, for a total of 47 genomes (Supplementary Data 2).

Benchmarking Universal Single-Copy Ortholog (BUSCO) evaluations were performed on each genome by compleasm (v0.2.7)^39^ with the odb12 database and the *Basidiomycota* lineage that has 2409 universal single-copy orthologs. For genomes with duplicated BUSCOs higher than 5%, the Redundans (v2020.01.28)^38^ program was used to identify alternative haplotypes. Haplotype alignments with an identity higher than 90% and coverage of more than 80% were removed using “--identity 0.9 --overlap 0.8” parameters. compleasm was again used to evaluate the purged genomes with the same parameters. The resulting duplicated BUSCOs were considered biological duplications (Supplementary Data 2). The haploid genomes (de-duplicated) were used in the following analyses.

Genomes with single-copy BUSCOs higher than 50% were used to construct the phylogeny. Melame1 (BUSCO S:1134,D:1147,F:33,I:0,M:95) and MecolCla_1 (BUSCO S:826,D:1452,F:34,I:0,M:97) were not included in this analysis owing to the high proportion of duplicated BUSCOs. The protein sequences of single-copy BUSCOs present in the remaining 45 genomes were extracted and further aligned using MAFFT (v7.525)^54^ with a focus on local alignments using the “--maxiterate 1000 --localpair” parameters. The phylogeny was constructed using the aligned BUSCOs and IQ-TREE (v2.4.0)^40^. Each BUSCO alignment was treated as an individual partition^57^, and ModelFinder^58^ was used to identify the best model for each partition using the “-m MFP” parameter in IQ-TREE. The Shimodaira-Hasegawa-like approximate likelihood ratio test (SH-aLRT)^59^ and bootstrap tests were performed 1,000 times each with parameters “-bb 1000 -alrt 1000” in IQ-TREE. The bootstrap consensus tree was plotted by ggtree (v3.1.5)^60^ in R.

Transposable elements in each fungal genome were annotated using EDTA (v2.2.2)^61^ following the instructions of Benson et al. ^62^. The “--force 1” parameter was used to allow analysis of genomes with missing types of TEs (e.g., LTR-RTs). The “--anno 1” parameter was used to obtain whole-genome TE annotations. The “--cds” parameter was used to provide coding sequences to EDTA to improve TE annotations. Primary coding sequences of each genome were obtained from JGI MycoCosm and Tobias et al. ^3^. Solo and intact LTR-RTs were identified from whole-genome TE annotations using LTR_retriever (v3.0.1)^32^. The solo:intact ratio was calculated on the whole-genome level, which is the number of whole-genome solo LTRs divided by the number of whole-genome intact LTR-RTs. “inf” values were given to genomes without intact LTR-RTs. The divergence of intact LTR-RTs was estimated by LTR_retriever (v3.0.1)^32^ using the one-parameter Jukes-Cantor 69 model^63^.

### Simulating digital replicas of rust fungal genomes

Three fungal genomes were chosen for simulations, including *Puccinia coronata* f. sp. *avenae* (PuccoNC29_1), *Cronartium ribicola* (Crori1), and *A. psidii* (myrtle rust). TE libraries used for TE removal and simulating the fungal genomes were obtained via de novo TE annotation using EDTA (as described above). The TE Composition Approximation Mode of TEgenomeSimulator (v1.0.0) was used for simulations. The resulting digital replicas of the rust fungal genomes were further used to simulate LTR dynamics using PrinTE (see sections below). PrinTE operates at the genome-assembly level and is designed to model transposable element dynamics on haploid genome representations, which are appropriate for capturing species-level evolutionary change over long timescales. In the rust fungal analyses, we explicitly removed residual alternative haplotypes prior to TE annotation and simulation to ensure that each genome represented a single haploid assembly.

### Forward simulation of rust fungal and *Arabidopsis* genomes

#### Simulating LTR-RT dynamics in rust fungal genomes

To place the phylogeny on an absolute time scale (Fig. 7a), the maximum-likelihood tree (Fig. 4a) was pruned with nw_prune from the Newick Utilities (v1.6)^64^ to retain the 18 taxa spanning the clades that contain the three focal species (*C. ribicola*, *A. psidii*, and *P. coronata*). Substitution branch lengths were then converted to absolute time with PATHd8^65^, which estimates divergence times by mean-path-length smoothing and does not assume a global molecular clock. Four internal nodes were assigned fixed ages as secondary calibration points, which were taken from the time-calibrated *Basidiomycota* phylogeny of He et al.^66^. Ancestral states including genome size and LTR divergence distribution were then reconstructed on the time-scaled tree using PrinTE utilities, “ancestral_reconstruction_gs.R” and “ancestral_reconstruction_ltr_age.R”, which used fastAnc in phytools (v2.4.4)^67^ and projected onto the chronogram with contMap. Specifically, for each taxon, genome size was log_10_-transformed and treated as a continuous character, and the maximum-likelihood ancestral values with 95% confidence intervals were estimated at every internal node. Trees were handled by ape (v5.8.1)^68^ in R (v4.4.1).

#### Cronartium ribicola

The ancestral genome size at the MRCA of *C. ribicola* was estimated to be 180.27 Mb (95% CI: [127.18, 255.52] Mb), which is intermediate to the estimated sizes of extant species in this clade. The ancestral LTR-RT content of *C. ribicola* was estimated at 29.98 Mb, corresponding to 16.63% of the ancestral genome. PrinTE’s Synthetic Genome Mode was used to simulate this ancestral state as the burn-in, then the Grid Search Method was used to evolve a population of synthetic genomes to approximate the modern *C. ribicola* genome using a step size of 54,000 generations (“--st 54000”). The parameter search space encompassed insertion rates from 1 × 10^–9^ to 1 × 10^–17^ per bp per generation (“--ins-start 1e-9 --ins-end 1e-17”), deletion rates from 1 × 10^–9^ to 1 × 10^–17^ per bp per generation (“--del-start 1e–9 --del-end 1e-17”), solo LTR formation rate of 5% to 95% (“--sr-start 5 --sr-end 95”), and deletion length bias exponents of 0–10 (“--k-start 0 --k-end 10”). Using PrinTE’s dynamic Grid Search Method, we evaluated 800 parameter combinations spanning this search space. This yielded a total of 80,000 individual genome simulations, with each run simulating 1/100th of the total generations (54,000) to its MRCA. The best-fit parameter set was identified quantitatively by minimizing a Composite Distance Metric across all 80,000 simulations using “--alphas 0 0 10 1” to assign the highest weight to the LTR divergence distribution and, secondly, the genome size. The optimal parameters to mimic the *C. ribicola* genome were identified as insertion rate 6.08 × 10^–12^ per bp per generation, deletion rate 1.14 × 10^–12^ per bp per generation, solo LTR formation rate of 35%, and deletion length bias of 3. The genome size trajectory during the best-fit simulation rose linearly from 183.56 Mb at generation 0 to 211.85 Mb at generation 5,400,000.

#### Puccinia coronata

***avenae****.* Using PrinTE’s ancestral reconstruction, the *P. coronata* MRCA was estimated to have a genome size of 109.5 ± 8.0 Mb (95% CI: [101.4, 118.3] Mb) with LTR-RT content comprising 14.1% of the ancestral genome (15.5 Mb of 109.5 Mb). PrinTE’s Synthetic Genome Mode was used to simulate this ancestral state as the burn-in, then the Grid Search Method was used to evolve a population of synthetic genomes to approximate the present-day *P. coronata* genome with a step size of 13,000 generations (“--st 13000”). The four primary parameters varied were insertion rate (ranging from 5 × 10^–12^ to 1 × 10^–17^ per bp per generation; “--ins-start 5e-12 --ins-end 1e-17”), deletion rate (ranging from 1 × 10^–10^ to 1 × 10^–17^; “--del-start 1e-10 --del-end 1e-17”), solo LTR formation rate (ranging 5–95%; “--sr-start 5 --sr-end 95”), and deletion length bias (ranging 0–10; “--k-start 0 --k-end 10”). We evaluated each parameter set by simulating evolution over 260,000 generations with default weights (“--alphas 0 0 10 1”), representing the divergence time between the two extant *Puccinia* strains. For each parameter combination, we scored the resulting simulated genome against the reference present-day *P. coronata* strain NC29_1 using the Composite Distance Metric. The best-fit parameter set was identified as: insertion rate = 1.325 × 10^–11^, deletion rate = 3.35 × 10^–14^, with solo LTR formation rate = 40% and deletion length bias = 5. At these parameters, the simulated genome was 111.2 Mb in size, closely matching the 113.2 Mb reference strain NC29_1.

#### Austropuccinia psidii

The MRCA of *A. psidii* and *Puccinia* is estimated to be 114 million generations old. Different from *C. ribicola* and *P. coronata*, *A. psidii* has the maximal TE insertion roughly 1.4 million generations ago, followed by a recent decline in young elements (Fig. 4d), which requires a two-phase simulation. To reconstruct the ancestral state, we first removed intact LTR-RTs younger than 1.4 million generations, then subtracted 1.4 million generations from the age of the remaining elements, which produced the ancestral LTR divergence distribution at 1.4 million generations ago. We then used this reconstructed divergence distribution to further reconstruct the ancestral state at MRCA, which was 113 million generations before the activity decline stage. PrinTE’s ancestral reconstruction yielded the ancestral haploid genome size of approximately 244 Mb (95% CI: [169.3, 404.1] Mb) at MRCA, substantially smaller than the present-day *A. psidii* genome (1,018.1 Mb).

For the expansion phase, we supplied the ancestral *A. psidii* state at MRCA as burn-in to run the Grid Search Method over insertion rate (ranging from 1 × 10^–12^ to 1 × 10^–17^ per bp per generation; “--ins-start 1e-12 --ins-end 1e-17”), deletion rate (ranging from 1 × 10^–10^ to 1 × 10^–17^; “--del-start 1e-10 --del-end 1e-17”), solo LTR formation rate (ranging 5-95%; “--sr-start 5 --sr-end 95”), and deletion length bias (ranging 0–10; “--k-start 0 --k-end 10”). We evaluated each parameter combination by simulating evolution over 113 million generations using a step size of 1.13 million generations (“--st 1130000”), representing the phylogenetically inferred divergence time between the *A. psidii* MRCA and the ancestral state before the contraction phase. Because the empirical LTR-RT divergence distribution of *A. psidii* shows a pronounced decline in recently inserted elements, we deliberately allowed the expansion phase to produce a genome larger than the present-day size, creating the opportunity to model a subsequent contraction phase. From the peak genome size achieved at generation 113 million, we launched a second Grid Search Method run to identify the rates governing the most recent contraction phase from around 1.4 million generations ago. We searched over the same parameter space (insertion rate, deletion rate, solo LTR formation rate, and deletion length bias), and each parameter combination was evolved forward from the expansion-phase endpoint and scored against the present-day *A. psidii* reference genome using a step size of 100,000 generations (“--st 100000”).

For the expansion phase, the best-fit parameter set was identified as insertion rate = 2.3 × 10^–12^, deletion rate = 4 × 10^–15^, with solo LTR formation rate = 85% and deletion length bias = 0. This parameter combination expanded the ancestral genome from 244 Mb to 1,082 Mb. For the contraction phase, the best-fit parameter set was identified as insertion rate = 1.2 × 10^–12^, deletion rate = 1.2 × 10^–11^, solo LTR formation rate = 80%, and deletion length bias = 8. This phase contracted the genome from its peak (1082 Mb) to 977 Mb.

#### Simulating LTR-RT dynamics in Arabidopsis genomes

*Arabidopsis* and *Camelina hispida* genomes were downloaded from NCBI (GCA_023657505.1, GCA_000001735.2, GCA_905216605.1, GCA_964271285.1, and GCA_026151145.1). Genomes were processed using BUSCO to identify shared single-copy orthologs and IQ-TREE to infer the maximum likelihood phylogeny, similar to what is described in the rust fungal analysis. PATHd8 was used to convert the genetic distance branch lengths to a time-scaled ultrametric distance tree with time-constraining calibrations from Koch et al.^69^. Ancestral state reconstruction of the genome size and LTR-RT states was determined using PrinTE utilities. PrinTE was used to create a digital replica of the ancestral genome, and the Grid Search Method was used to train the surrogate model to search for optimal parameters using “--ins-start 6e-12 --ins-end 1e-14 --del-start 1.5e-11 --del-end 1e-14 --sr-start 5 --sr-end 95 --sr-step 5 --k-start 0 --k-end 16”. PrinTE’s Composite Distance Metric identified parameters insertion rate = 1.0 × 10^–14^, deletion rate = 6.0 × 10^–14^, solo LTR formation rate = 15%, and deletion length bias (k) = 12 as producing a simulated result that is most similar to the present-day *A. thaliana* genome.

### Reporting summary

Further information on research design is available in the Nature Portfolio Reporting Summary linked to this article.

## Supplementary information

Supplementary Information

Description of Additional Supplementary Files

Supplementary Data 1

Supplementary Data 2

Reporting Summary

Transparent Peer Review file

## Source data

Source Data

## Data Availability

Manuscript figures, and code to re-generate them, are available at https://github.com/cwb14/PrinTE, under the GPL-3.0-or-later license. Source data are provided with this paper.

## Code availability

PrinTE v1.0.3, used for all analyses in this study, is available at https://github.com/cwb14/PrinTE and archived at https://doi.org/10.5281/zenodo.23044640^70^ under the GPL-3.0-or-later license. Kmer2LTR is available at https://github.com/cwb14/Kmer2LTR and archived at https://doi.org/10.5281/zenodo.22946719^71^ under the same license.
