# Supplementary material for: PrinTE: a forward simulation framework for studying the role of transposable elements in genome expansion and contraction
Source: Nat Commun. 2026 Nov 2;17:11126. doi: 10.1038/s41467-026-78884-0 (PMC13728204; doi:10.1038/s41467-026-78884-0)
Supplement: Supplementary file 1 [file 41467_2026_78884_MOESM1_ESM.pdf]

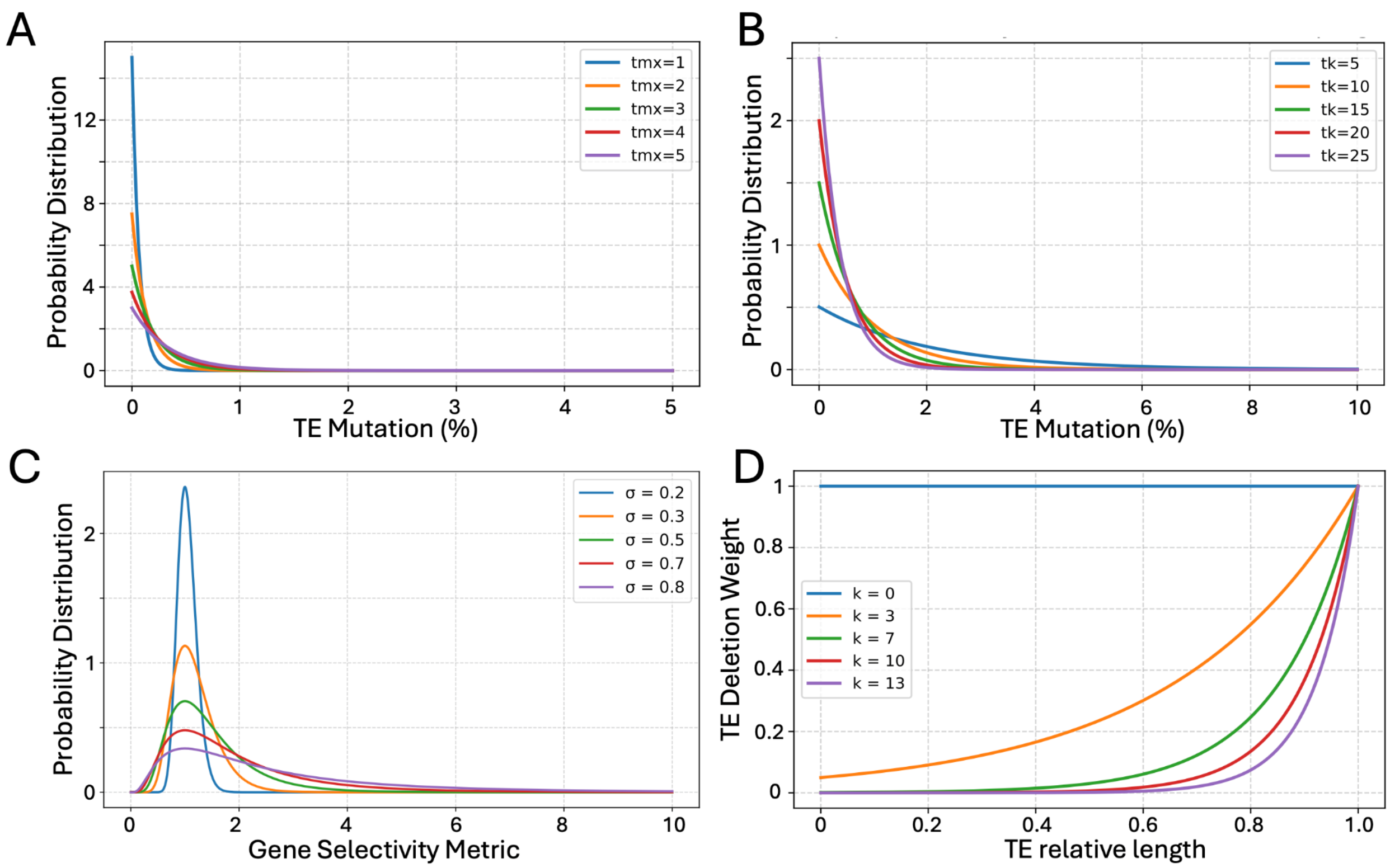

Supplementary Figure 1. PrinTE evolutionary modeling based on customizable probability distributions. The distribution of mutation probabilities for TEs inserted into the burn-in (generation zero) genome is modulated by an exponential decay function, where maximum mutation of TEs ('`--tmx`'; **A**) and TE mutation bias ('`--tk`'; **B**) are used to customize the distribution. (**C**) Gene selectivity is scored using a log-normal distribution where σ controls the distribution of selective scores. The higher the Gene Selectivity Metric value, the higher the selection coefficient of TE insertions into genes. A narrower density distribution indicates that most gene mutations by TEs are nearly neutral or only slightly deleterious. (**D**) The element length determines a TE's probability of deletion, and the strength of this length bias is set by the deletion length bias parameter ("`--k`"). A higher k will impose a higher weight on deleting long TEs. Setting k=0 means that all TEs have an equal probability of being deleted.

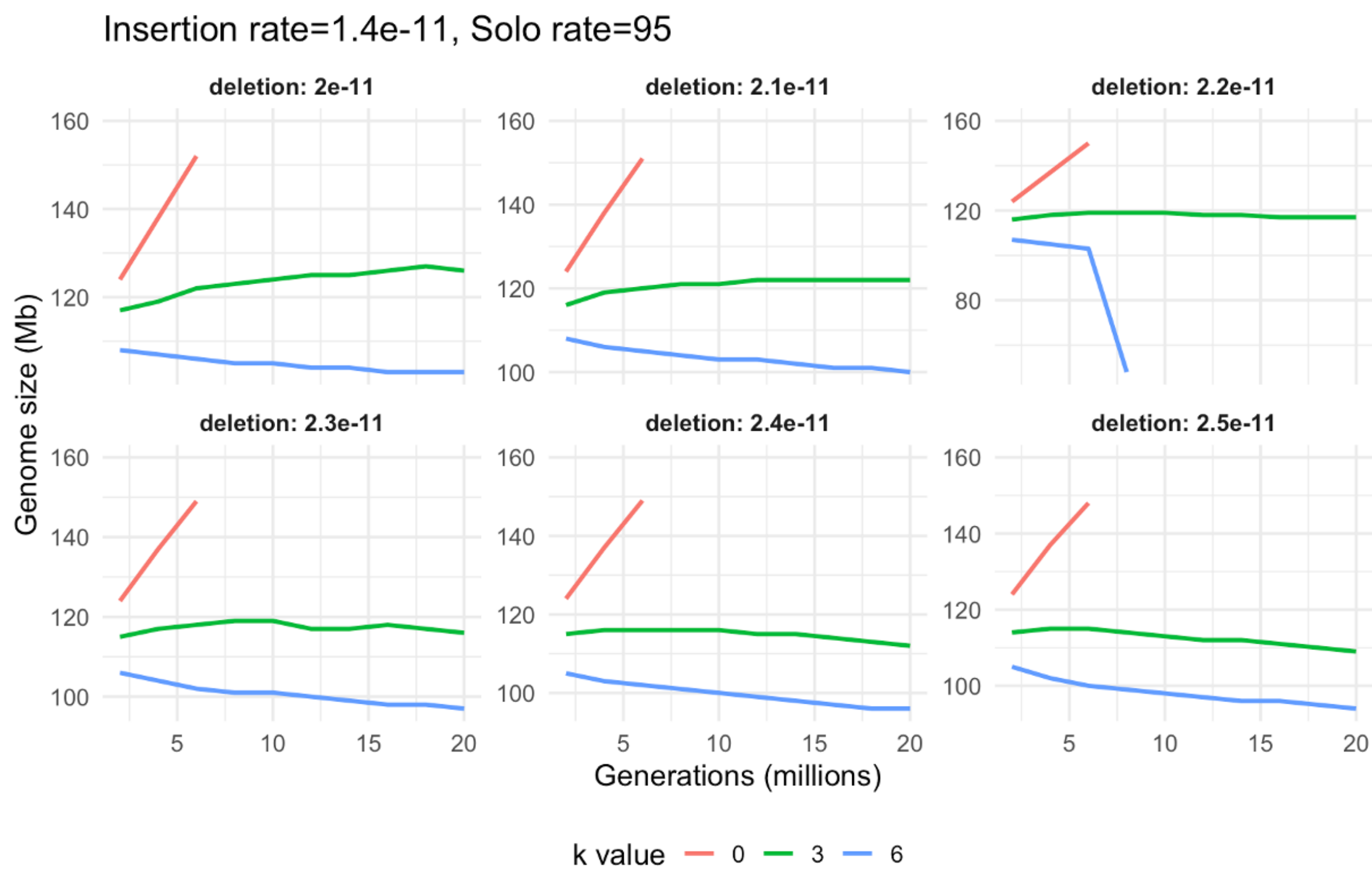

Supplementary Figure 2. Variation of genome size simulated using PrinTE under fixed insertion rate (1.4×10^{-11} per bp per generation), varying deletion rates (2×10^{-11} - 2.5×10^{-11} per bp per generation), varying deletion length bias (k=0, 3, or 6), and a fixed solo LTR formation rate (95%).

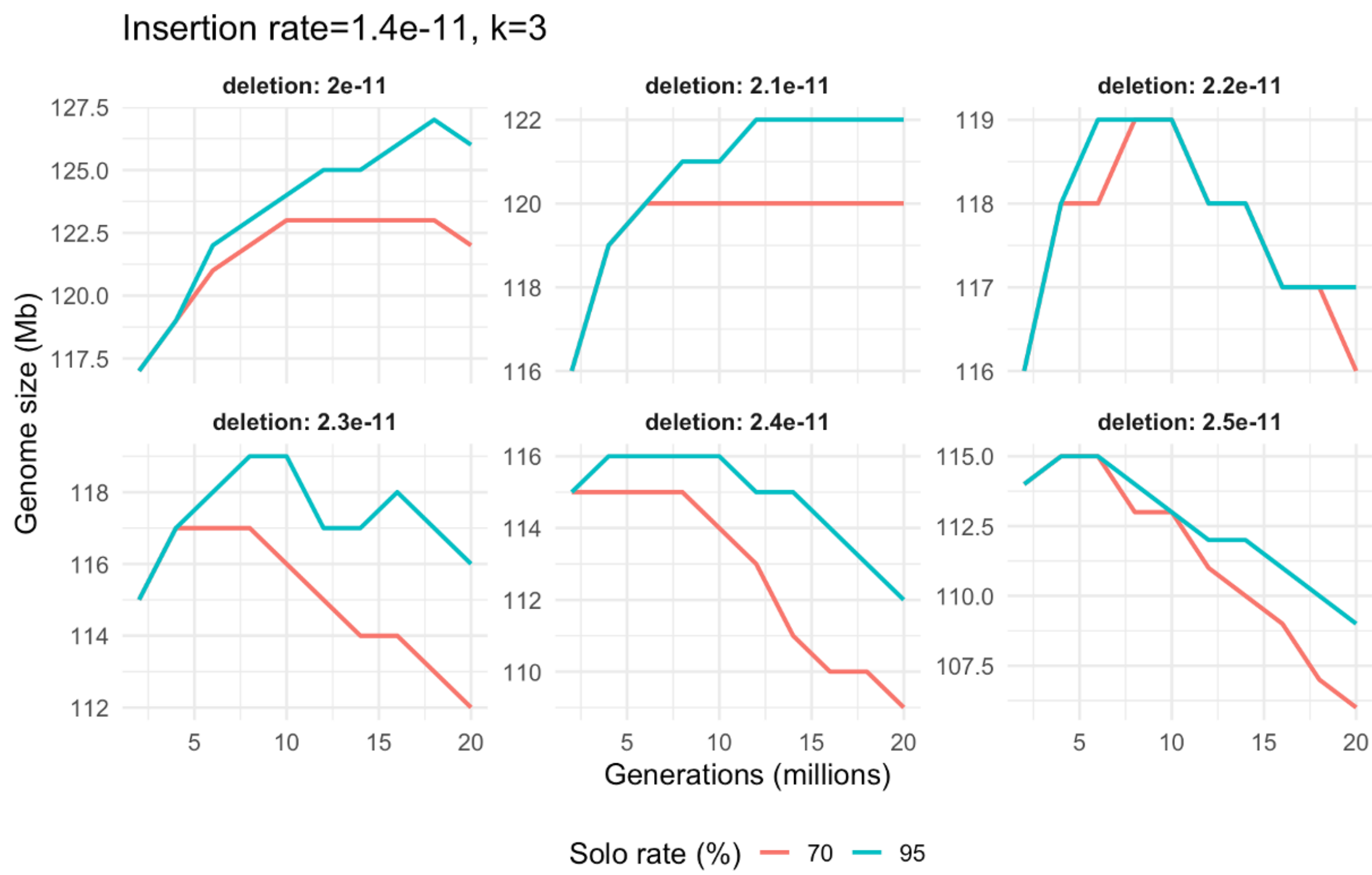

Supplementary Figure 3. Variation of genome size simulated using PrinTE under fixed insertion rate (1.4×10^{-11} per bp per generation), varying deletion rates (2×10^{-11} - 2.5×10^{-11} per bp per generation), fixed deletion length bias (k=3), and varying solo LTR formation rates (70% and 95%).

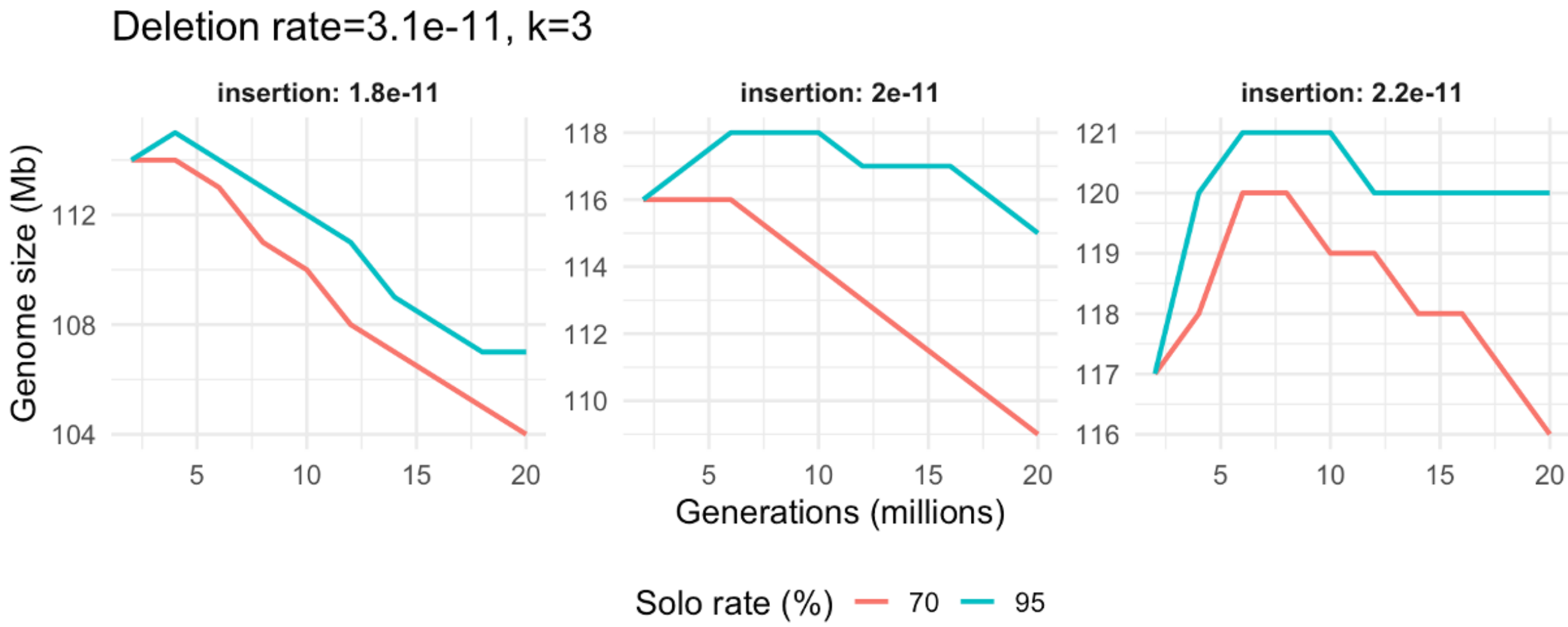

Supplementary Figure 4. Variation of genome size simulated using PrinTE under varying insertion rates (1.8×10^{-11}-2.2×10^{-11} per bp per generation), a fixed deletion rate (3.1×10^{-11} per bp per generation), fixed deletion length bias (k=3), and varying solo LTR formation rates (70% and 95%).

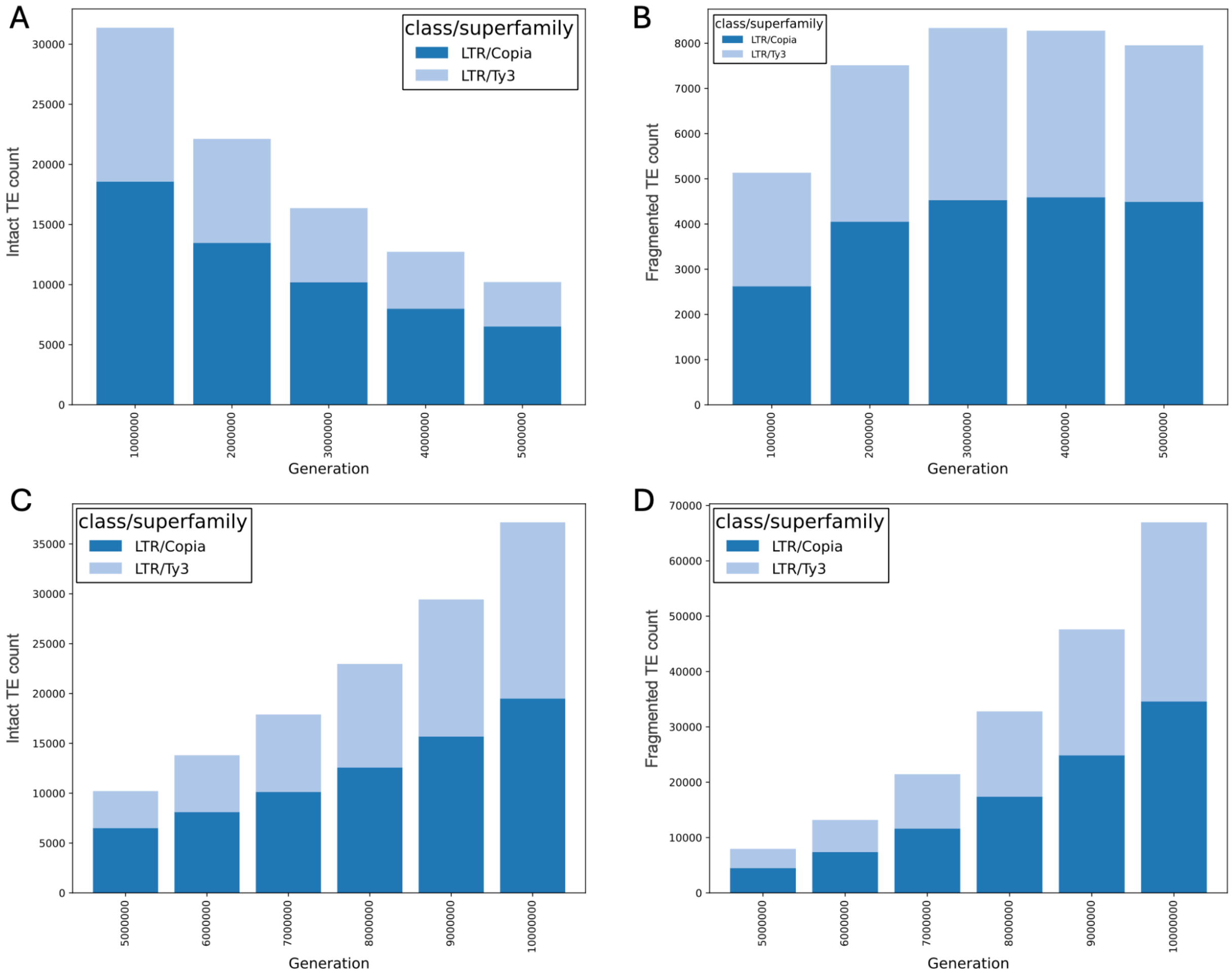

Supplementary Figure 5. TE counts across generations in PrinTE simulations. Intact (**A**) and fragmented (**B**) TE counts in a simulated genome experiencing a genome size decrease. Intact (**C**) and fragmented (**D**) TE counts in a simulated genome experiencing a genome size increase. The data are the same as those used in **Fig. 3**. (**A**) and (**B**) correspond to the first five million generations, and (**C**) and (**D**) correspond to the second five million generations.

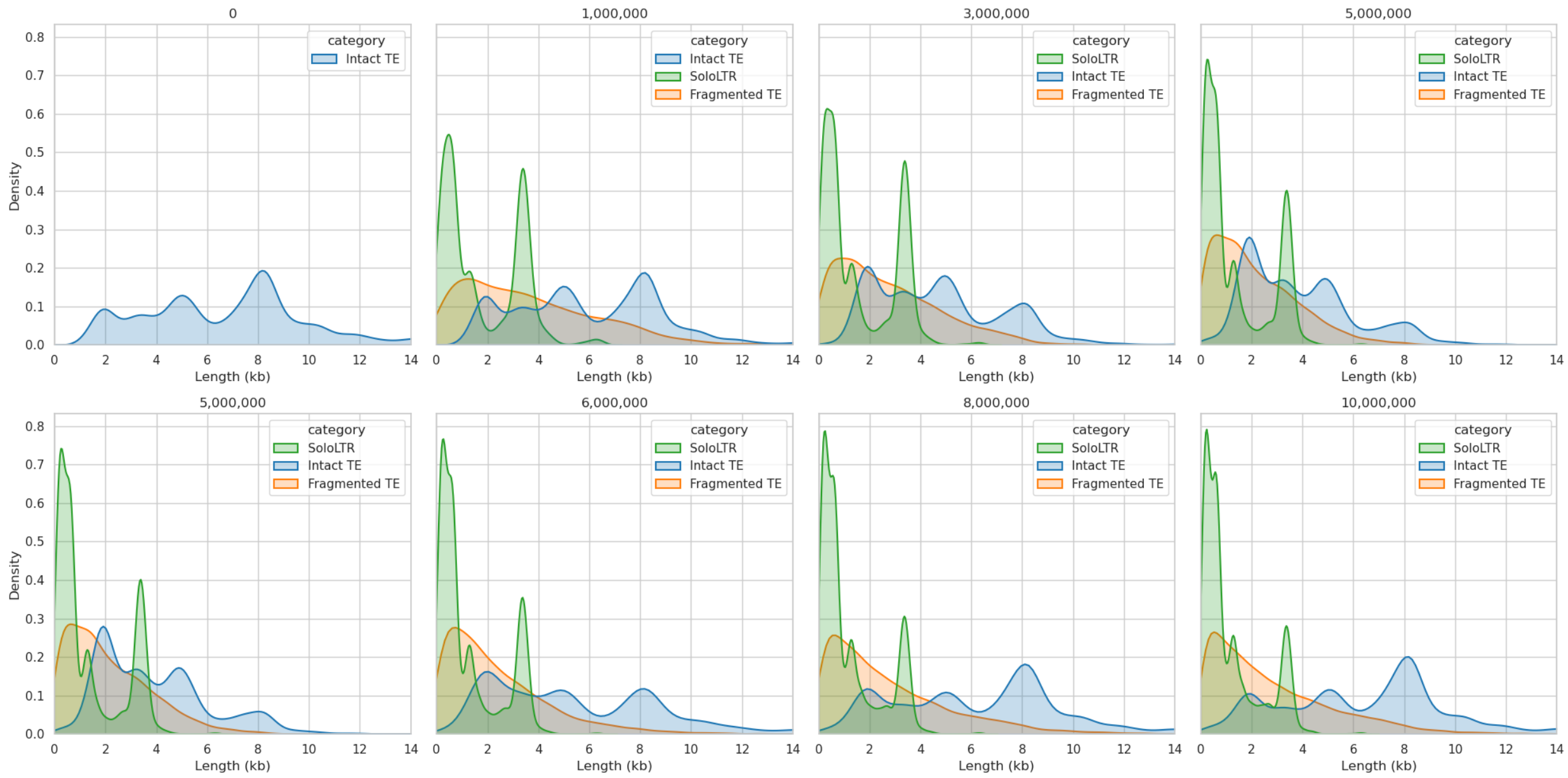

Supplementary Figure 6. The density of PrinTE feature lengths for intact and fragmented LTR-RTs and solo LTRs. The top row shows a period of genome contraction, characterized by progressive buildup of solo LTRs and fragmented LTR-RTs (0 to 5 million generations are shown). The bottom row shows feature density during a subsequent period of genome expansion. The abundances of solo LTRs and fragmented LTR-RTs are maintained, but the proportion of longer and intact LTR-RTs increases through time.

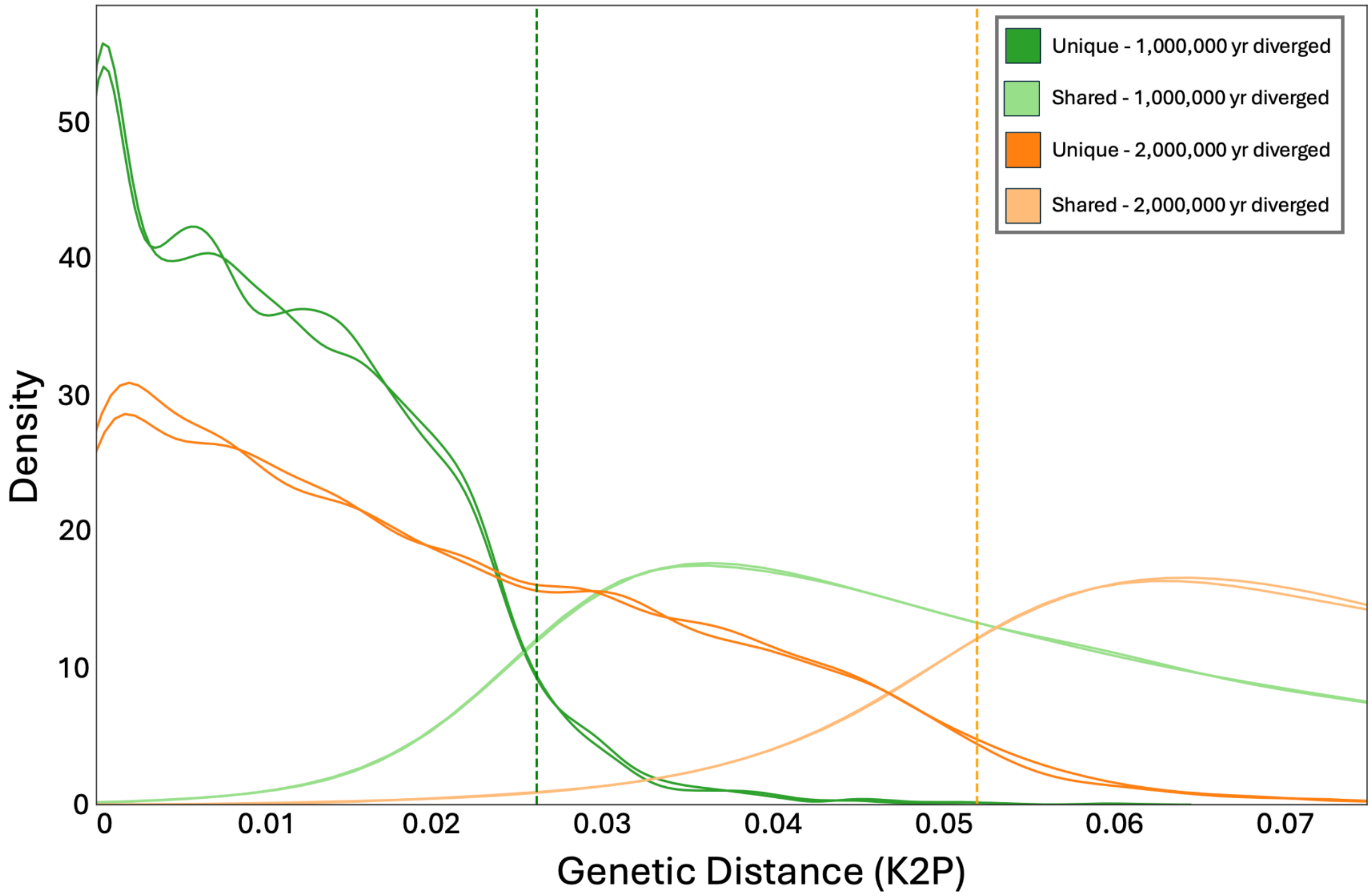

Supplementary Figure 7. PrinTE-simulated LTR-RT divergence between two lineages after one million (green) and two million (orange) generations following reproductive isolation. Shared LTR-RTs are retained in both lineages and derived from existing TEs of the most recent common ancestor (MRCA). Unique LTR-RTs are only present in one of the lineages and were transposed after lineage divergence. The vertical dashed line shows the expected LTR divergence of the MRCA at each time point.

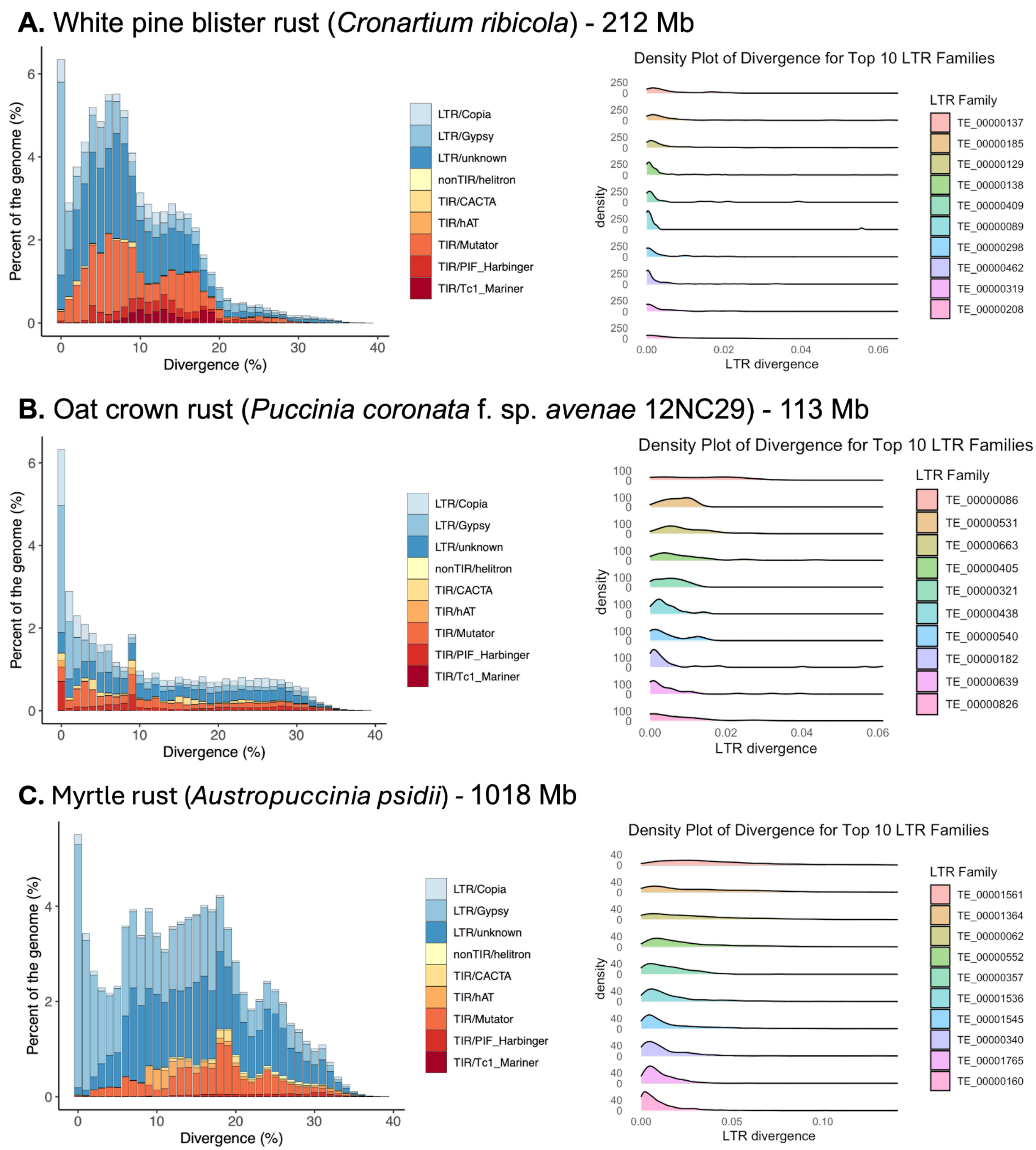

Supplementary Figure 8. Transposable element divergence in *Cronartium ribicola* (**A**, white pine blister rust), *Puccinia coronata* f. sp. *avenae* (**B**, oat crown rust), and *Austropuccinia psidii* (**C**, myrtle rust) genomes. The LTR divergence distribution of the top 10 most abundant intact LTR-RT families in each genome is shown, and they are not shared between genomes.

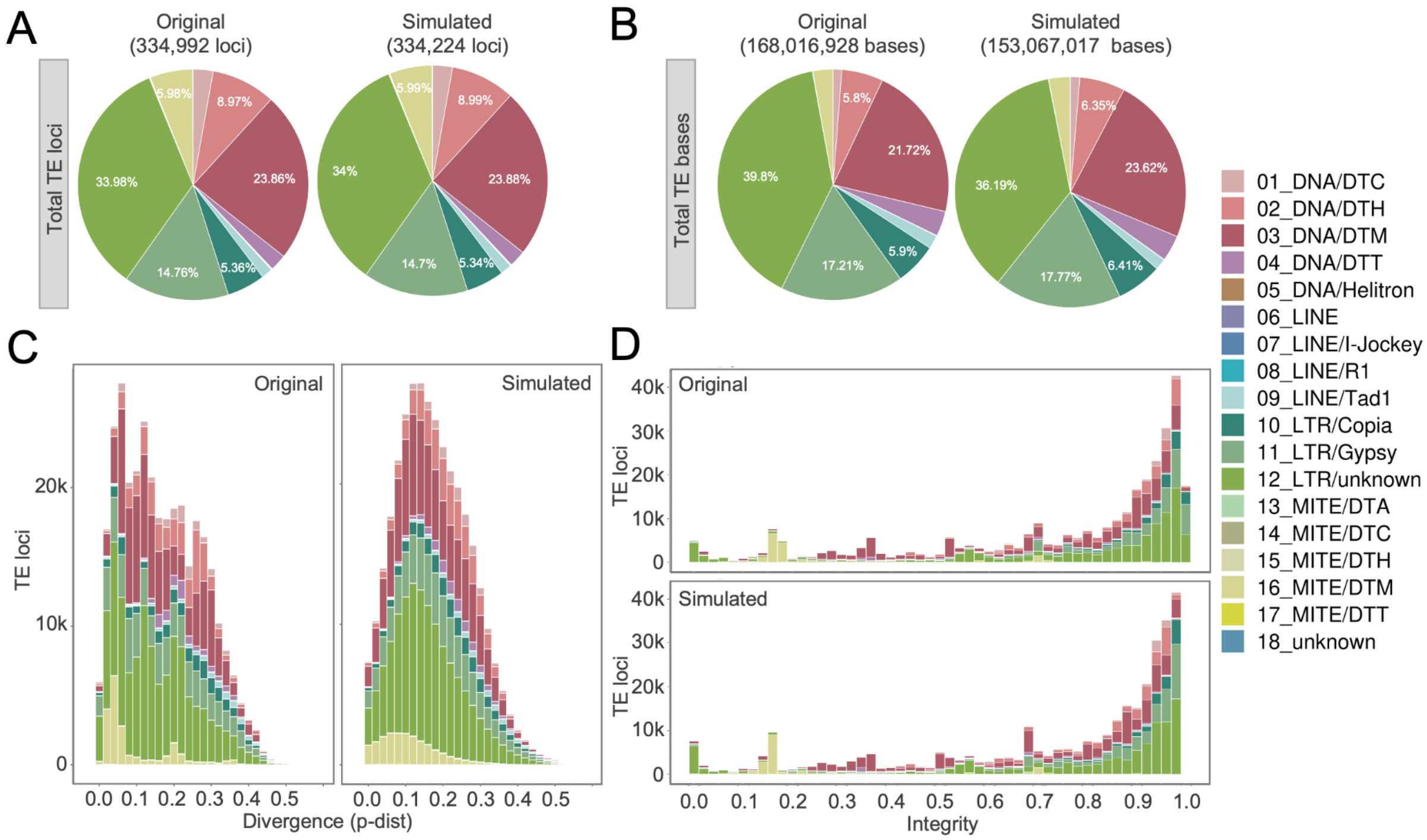

Supplementary Figure 9. TE Composition Approximation for the *Cronartium ribicola* genome. (**A**) Comparison of total TE loci between the original and simulated genomes. (**B**) Comparison of total TE bases between the original and simulated genomes. (**C**) TE sequence divergence distribution colored by TE superfamily. (**D**) The distribution of TE integrity, which is the length ratio relative to the consensus sequence, is colored by TE superfamily as in (**C**).

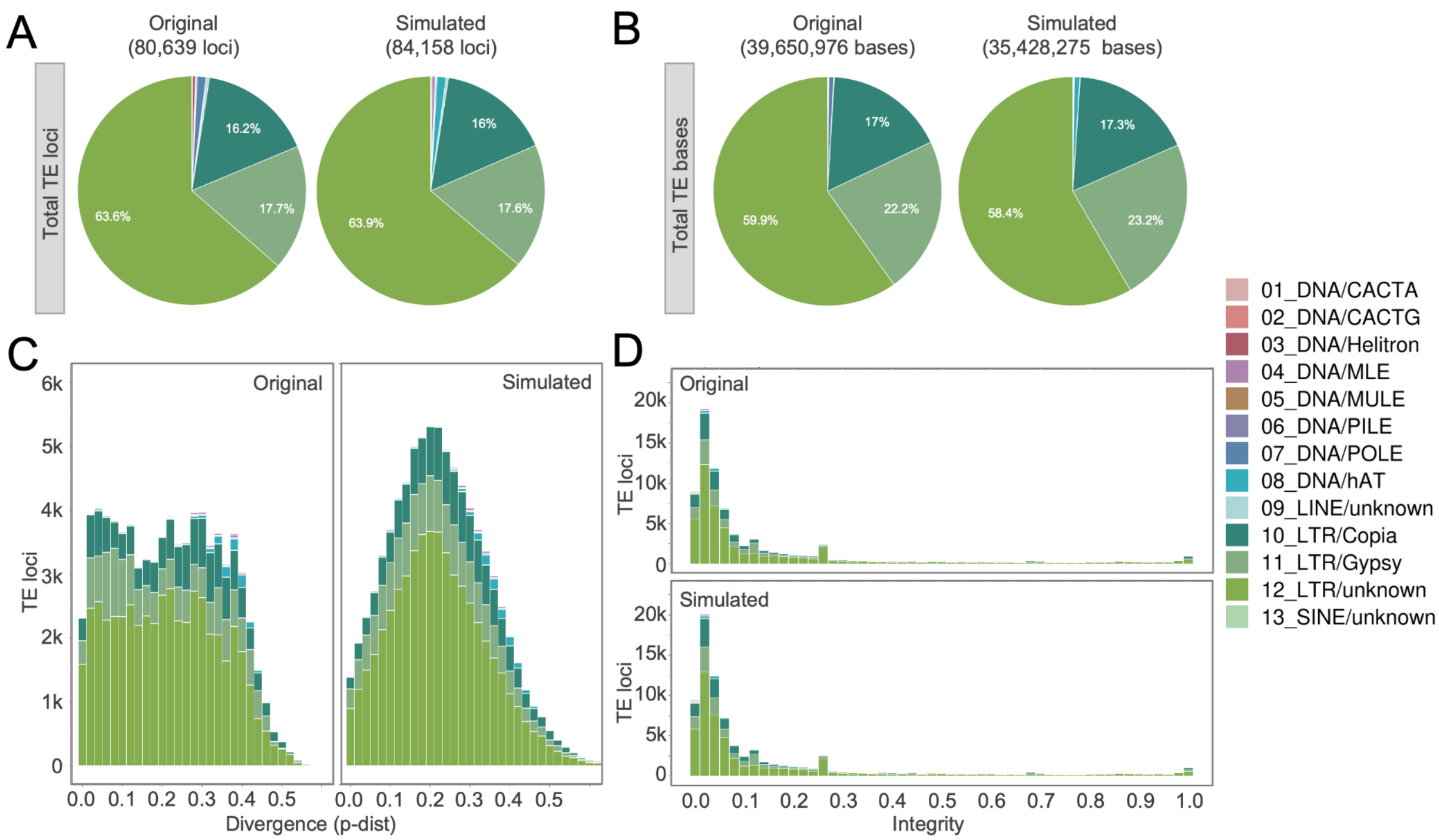

Supplementary Figure 10. TE Composition Approximation for the *Puccinia coronata* f. sp. *avenae* genome. (**A**) Comparison of total TE loci between the original and simulated genomes. (**B**) Comparison of total TE bases between the original and simulated genomes. (**C**) TE sequence divergence distribution colored by TE superfamily. (**D**) The distribution of TE integrity, which is the length ratio relative to the consensus sequence, is colored by TE superfamily as in (**C**).

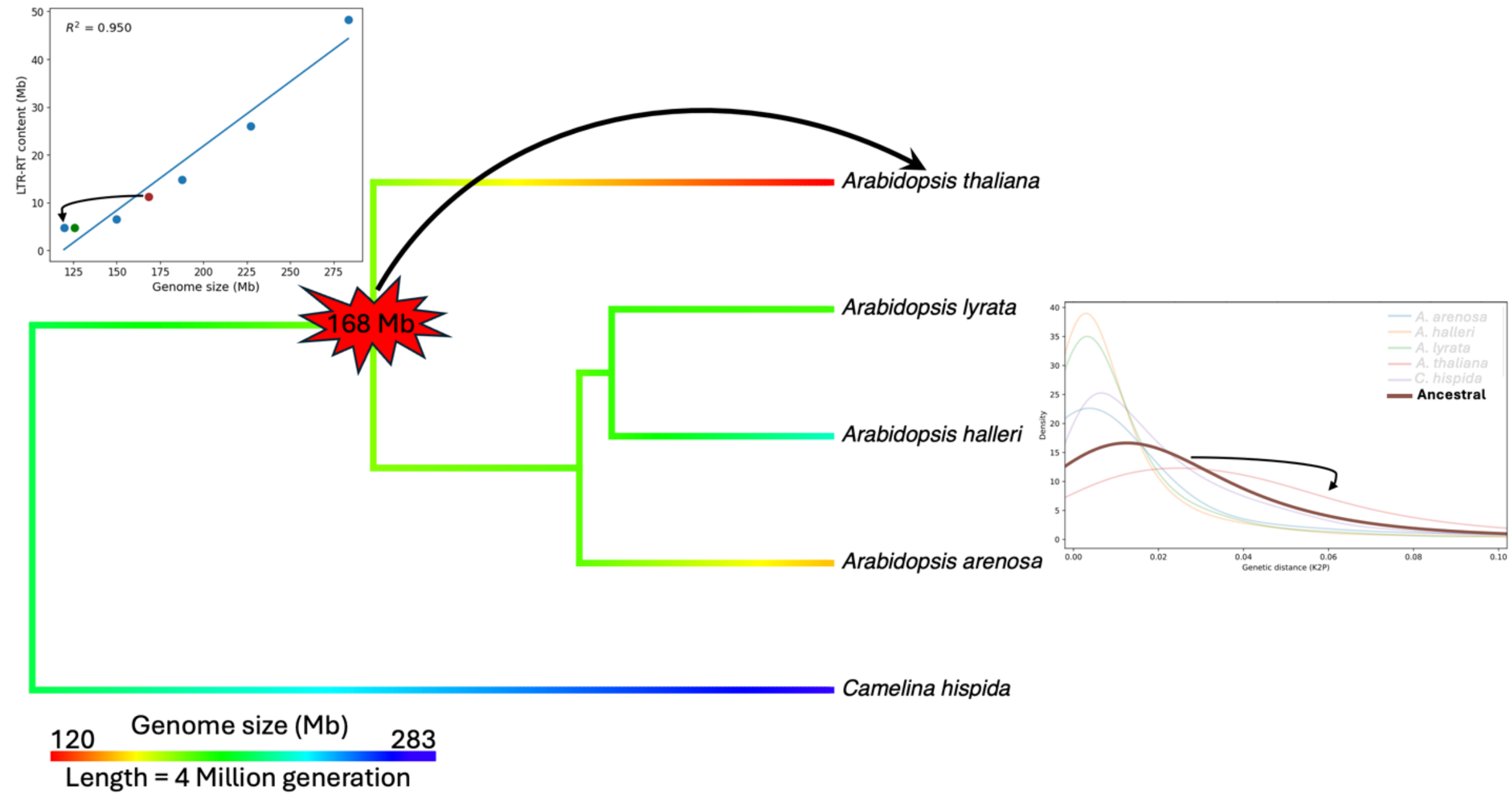

Supplementary Figure 11. Forward simulation from the ancestrally reconstructed MRCA of *Arabidopsis thaliana* to identify the LTR-RT conditions that best recreate *A. thaliana*. The regression shows a strong relationship between genome size and LTR-RT content (R^2=0.95), indicating that LTR-RTs play a role in genome size in *A. thaliana.* In the inset panel, the red point shows the ancestrally reconstructed common ancestor. The green point highlights the composition of the top-performing simulation and the arrow points to *A. thaliana*. The time-scaled phylogeny shows four *Arabidopsis* genomes and *Camelina hispida* as an outgroup and is colored by genome size. The density plot shows the LTR divergence distribution of the five present-day genomes and also the ancestrally reconstructed *A. thaliana* common ancestor. Black arrows point to the present-day *A. thaliana* genome, and highlight the desired simulation direction, from the MRCA to *A. thaliana*.

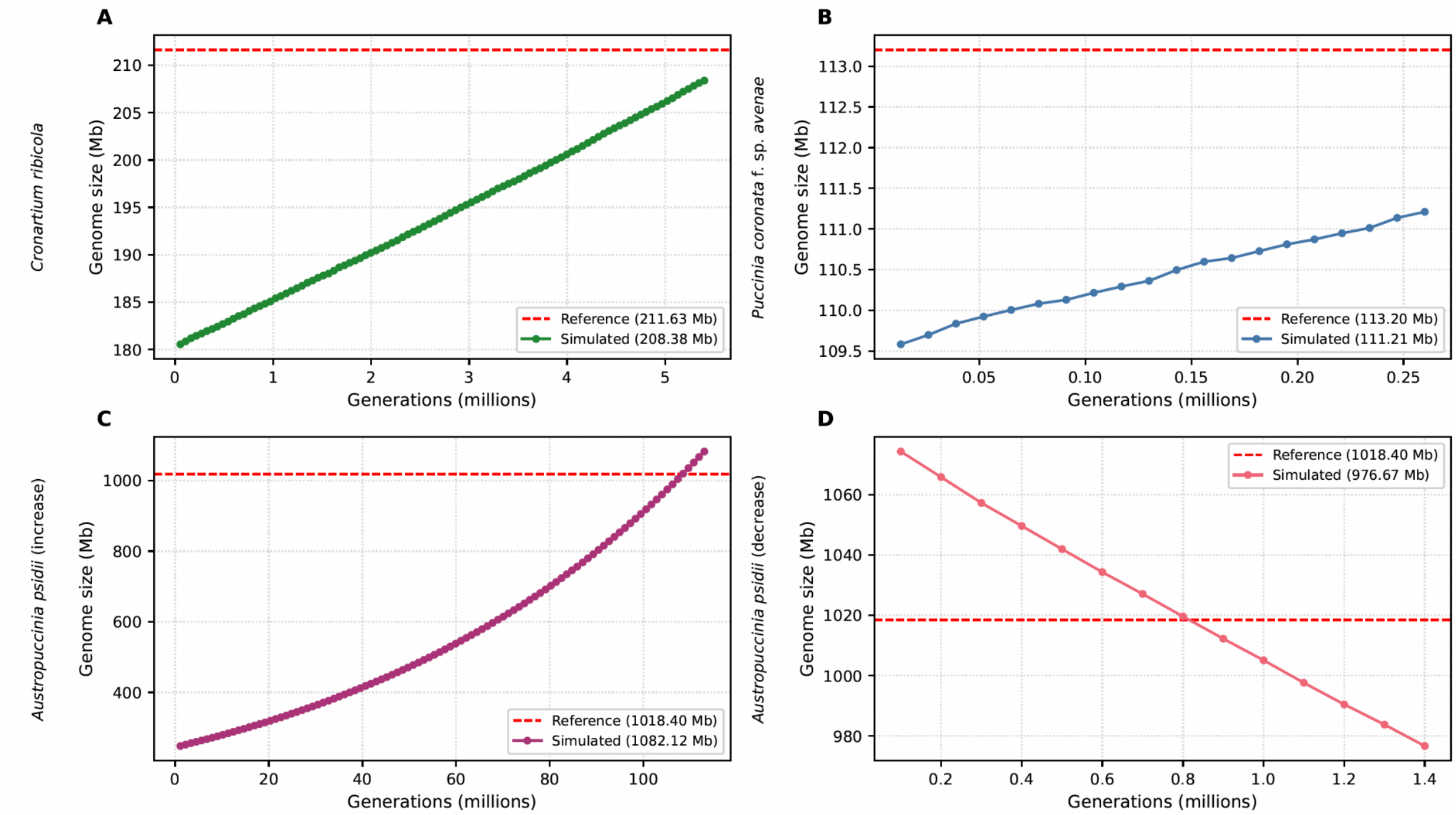

Supplementary Figure 12. Genome size change driven by LTR-RT insertion-deletion dynamics of the top-performing PrinTE simulation for *Cronartium ribicola* (A)*, P. coronata* f. sp. *avenae* (B), and *Austropuccinia psidii* (C, D). The *A. psidii* simulation is partitioned into an expansion phase, in which genome size increases **(C),** and a contraction phase, in which genome size decreases**(D)**.

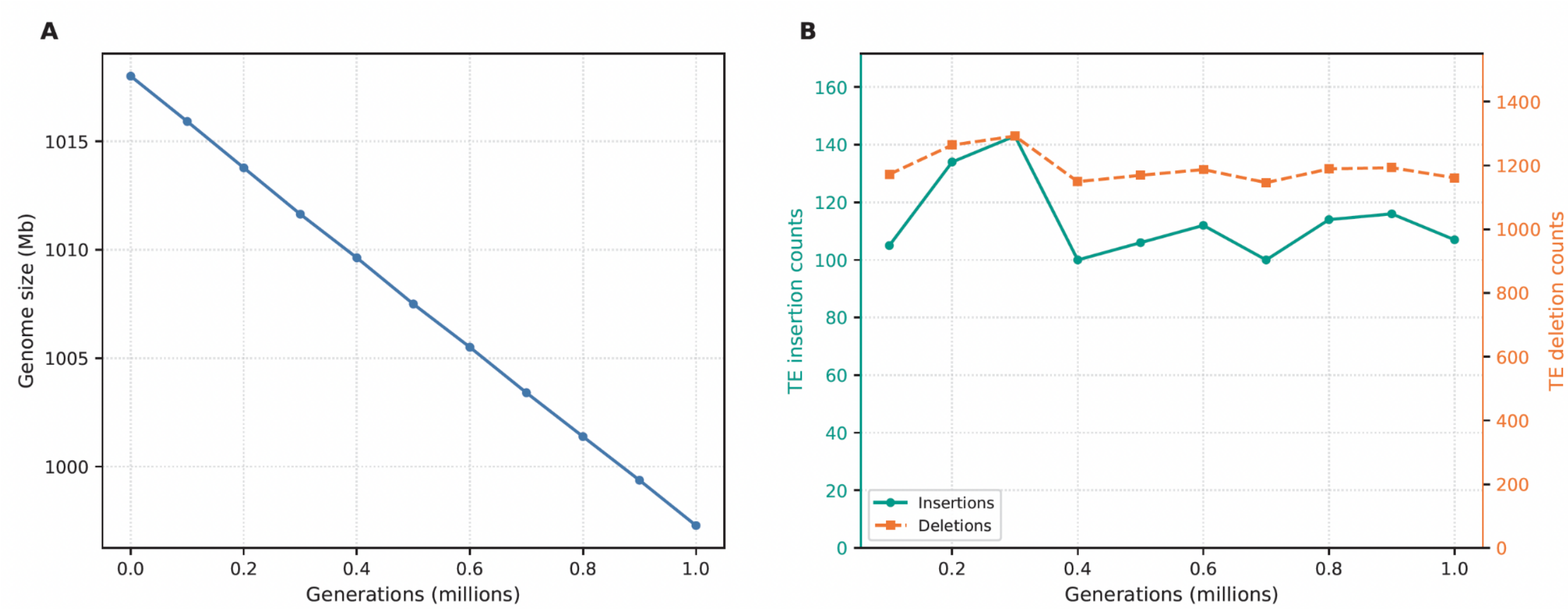

Supplementary Figure 13. Forward simulation using empirically inferred insertion and deletion rates predicts genome-size dynamics and TE turnover in the upcoming one million generations of *A. psidii* (myrtle rust) evolution. (**A**) Genome size declines monotonically from 1,018 Mb to 997 Mb, a net loss of 21 Mb (2%). (**B**) TE insertion (teal, left axis) and TE deletion (orange, right axis) events drive the net genome contraction shown in (**A**), with deletions exceeding insertions by roughly an order of magnitude (11,922 versus 1,137 events in total; a ~10.5× preference for deletion).

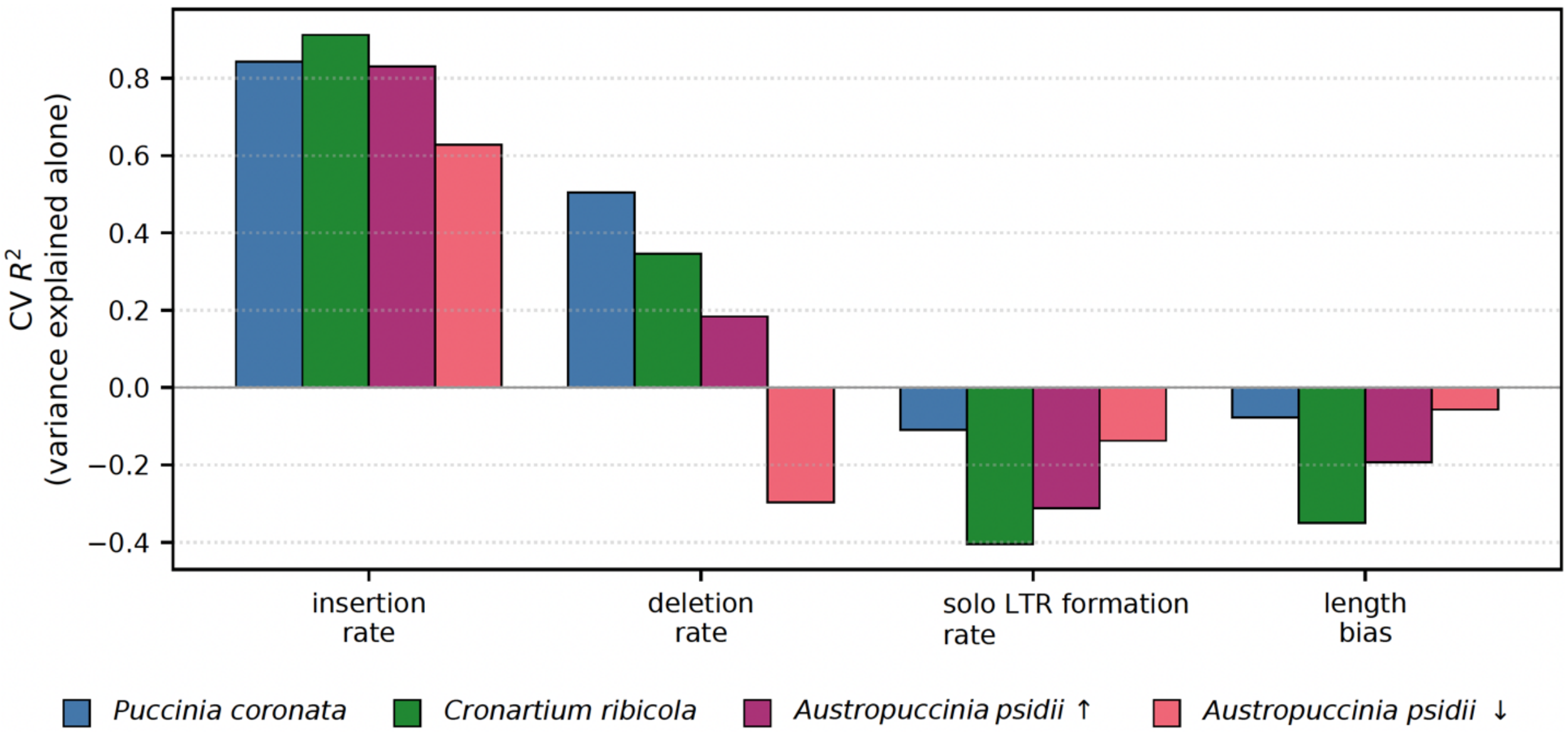

Supplementary Figure 14. Sensitivity analysis of each LTR-RT simulation parameter. The single-parameter cross-validated R^2 is used to measure how much of the simulation fit a single parameter can explain by itself. Insertion rate alone explains most of the fit in all four simulations (R^2 = 0.6-0.9), deletion rate explains a smaller share, and the solo LTR formation rate and the deletion length bias explain essentially nothing on their own.
