# Supplementary material for: PrinTE: a forward simulation framework for studying the role of transposable elements in genome expansion and contraction
Source: Nat Commun. 2026 Nov 2;17:11126. doi: 10.1038/s41467-026-78884-0 (PMC13728204; doi:10.1038/s41467-026-78884-0)
Supplement: Supplementary file 2 [file 41467_2026_78884_MOESM2_ESM.pdf]

Description of Additional Supplementary Files

Title: Supplementary Data 1

Description: Metadata of Pucciniomycotina genomes downloaded from Mycocosm.

Title: Supplementary Data 2

Description: Summary statistics of Pucciniomycotina genomes used in this study.
