# Supplementary material for: PrinTE: a forward simulation framework for studying the role of transposable elements in genome expansion and contraction
Source: Nat Commun. 2026 Nov 2;17:11126. doi: 10.1038/s41467-026-78884-0 (PMC13728204; doi:10.1038/s41467-026-78884-0)
Supplement: Supplementary file 4 [file 41467_2026_78884_MOESM6_ESM.pdf]

nature portfolio

Peer Review File

PrinTE: A Forward Simulation Framework for Studying the Role of Transposable Elements in Genome Expansion and Contraction

Corresponding Author: Professor Shujun Ou

This file contains all reviewer reports in order by version, followed by all author rebuttals in order by version.

Version 0:

Reviewer comments:

Reviewer #1

(Remarks to the Author)
The authors present PrinTE, a forward-time simulator that operates on genome sequences to model TE insertions, deletions (including solo-LTR formation), truncations, and substitutions under fixed and variable-rate regimes. They benchmark its accuracy report good computational performance, and apply the framework to Pucciniomycotina fungi. Using simulations constrained by observed LTR divergence spectra, they infer contrasting TE histories for Cronartium ribicola (rapid turnover with near-stable size), Puccinia coronata f. sp. avenae (slow turnover), and Austropuccinia psidii (long expansion followed by a recent contraction). Across genomes, they find strong positive correlations between genome size and multiple LTR metrics, including a near-linear relation with solo-LTR count.

This manuscript introduces PrinTE, a new forward-time genome evolution simulator aimed at modeling TE dynamics, especially focusing on LTR retrotransposons. The authors provide a comprehensive study of rust fungi genomes and present PrinTE as a versatile tool for modeling genome size variation. While the manuscript is technically sound and provides rich simulation and case-study results, several conceptual clarifications, and broader validations beyond rust genomes are required to support the claims of generalizability and usability across different taxonomic groups.

Major Comments
Scope of Tool and Generalization Beyond Rust Fungi
• In the abstract the authors claim that PrinTE is versatile. If the tool is versatile, I expect it to work for non-rust fungi. If the case study to compelling and thouroughly investigated I think the authors need to demonstrate the efficacy of PrinTE on broader species (Arabidopsis for instance, since it's used in the benchmarking). In addition, have the authors tested it on Ascomycetes with TE-rich genomes, such as Blumeria graminis? And what about fungal genomes with moderate TE content (20-30%)?
• Following the same train of thoughts – specifically inspired by this study case of fungal genome: How is Repeat-Induced Point (RIP) mutations would be handled by PrinTE? Can the authors comment of this? To support the claim of broad applicability, include validation or test cases beyond Pucciniomycotina, ideally from other fungal clades with different genome dynamics and TE regulation mechanisms (e.g., RIP-prone species).
• In general, I think the manuscript's background needs more information: I understand that the goal of this study is to present PrinTE but since the manuscript heavily focuses on rust fungi. It would benefit from explicitly stating this in the introduction and justifying the biological relevance as well as the challenges to infer TE dynamics in fungal genomes.
• The introduction also needs more about the host genome-TE arms race dynamics in terms of control mechanisms other than illegitimate recombination (IR). By the way IR is not even defined in the introduction. If that's an effective mechanism to remove LTRs, what about preventing insertions for instance by TE silencing and/inactivation? I think all these concepts need to be properly introduced for the reader to fully grasp what the study is testing.
• Additionally, the manuscript would benefit from a brief discussion on how PrinTE and Kmer2LTR perform on smaller genomes, such as ~40 Mb, particularly in fungi with RIP signatures. Can these tools detect divergence accurately in highly mutated TE landscapes, or are there limitations?

Specific comments
Highly technical abstract: I find the abstract too technical and it is difficult to get the message of the study, what's the

presentation of PrinTE and what are the outcome of the case study of its application in rust genomes. For instance, the inclusion of Pearson's r values in the abstract. This level of detail is too technical for an abstract.

L61-71: The paragraph conflates whole-genome duplication (WGD) and transposable element (TE)-driven expansion, making it difficult to distinguish their respective roles in plant genome evolution. Please clarify when genome size increases are due to WGD versus TE activity, ideally by separating these concepts more clearly. Also, the cited example of Arabidopsis is misleading, the referenced work addresses genus-level variation, not within-species mechanisms, as A. lyrata has a larger genome than A. thaliana.

L69: Why citing the commentary paper instead of the original research articles?

L133: The manuscript states that TE insertions into intergenic space incur no cost, whereas insertions into genic space do. However, it is unclear how regulatory regions (e.g., promoters) are handled. Since TE insertions upstream of genes can affect expression, please clarify how regulatory elements are defined in the model. Is there a distance threshold from genes that determines "no cost" zones?

L160-171: This section introduces the use of Kmer2LTR and SLiM to evaluate PrinTE's accuracy, but the methodology is currently unclear: How exactly was Kmer2LTR used to validate PrinTE's performance? Was PrinTE used to generate test genomes for Kmer2LTR, or was Kmer2LTR applied independently to real or SLiM-derived data? The manuscript mentions the Ti/Tv ratio in PrinTE but doesn't clearly explain how this relates to Kmer2LTR. Did the authors calibrate Kmer2LTR results using Ti/Tv, or was this parameter tested separately in PrinTE simulations? Regarding SLiM: Please clarify whether SLiM-simulated genomes were used to benchmark Kmer2LTR, to compare with PrinTE, or as control datasets. The workflow should be clearly structured in the text.

L212-219: It is unclear whether the 100 Mb genome simulation described here is the same one used to benchmark the performance and accuracy of PrinTE and Kmer2LTR in the earlier sections. Please clarify whether these simulations were part of the accuracy validation (e.g., Ti/Tv, divergence detection) or run independently for performance testing.

L228-229: Please specify the extent of the reduction in intact TEs. What was the initial TE coverage in the genome (e.g., % of genome or number of elements)? From Fig. S2A, it appears to be ~75% (please confirm). What exactly do "fragmented TEs" refer to in this context? Are these partial deletions, decayed sequences, or the result of nested insertions? Please clarify how they are defined and identified.

L231: Please specify that this second phase (elevated insertion rate) corresponds to the next 5 million generations to make the simulation timeline clear.

L233: How many generations did it take for the genome to return to its initial size? From the figure, it appears this happens around ~9M generations; does the 4M generations qualify as "rapid"? If starting with only intact TEs at the genome size minimum, how many generations would it take to recover?

L235: "k>0", Please define what the k parameter represents (it's not introduced in Fig. S3). What k value was used in this scenario?

L263-281: Consider moving this section on deletion parameters (rate, TE length bias, solo LTR rate) before the simulation results in Fig. 3. This would clarify the meaning and impact of the k parameter earlier for the reader.

L285: Please specify which "molecular features" are being referred to (e.g., purifying selection)? population dynamics? TE competition? Clarification would help contextualize the variable-rate model.

L288: On what empirical data or rationale is the starting genome model of Arabidopsis thaliana based? Please clarify what aspects of the real genome this simulation reflects.

L290: Please specify what simulation conditions were tested (e.g., insertion/deletion parameters, mutation models, etc).

L292: Why does the genome size decrease substantially more than it increases? Please comment on this asymmetry. Also, what is the TE landscape (e.g., ratio of solo/intact/fragmented TEs) when the genome size returns to baseline?
Section "Genome Size Evolution of Rust Fungi" This is a compelling case study, but to support claims of tool versatility, it would be important to test PrinTE on additional species (e.g., Arabidopsis or Ascomycetes). Also, how did the authors address: differences between short- and long-read assemblies, phased vs. unphased genomes?

L367-369: Is this truly counter-intuitive? Fig. S6 suggests that high retention of solo LTRs (e.g., solo = 95%) slows down purging and allows for larger genomes. This would explain the correlation. Additionally, the solo/intact ratio may not accurately reflect purging "intensity", it could also result from a burst-purge cycle. Please consider qualifying this interpretation.

L385: "LTR-RT insertion passed the current time" Please clarify what is meant by this phrase. Does it imply a decline in recent insertions, or that the peak insertion activity lies in the past?

L403-406: This contrast between high-integrity and fragmented TE landscapes is particularly interesting. Despite distinct TE

dynamics modes, both species exhibit genome size stability? Could the authors elaborate on how different strategies may converge on similar genome size maintainance?

L438-439: The simulation assumes constant TE insertion and deletion rates? Is this assumption evolutionary realistic? Please discuss its limitations or relevance over evolutionary timescales.

L458-458: Why was the “burn-in” simulation from 110 Mb only done for A. psidii and not for C. ribicola or P. coronata? Could the authors simulate a hypothetical common ancestral “rust fungus” genome for comparative trajectories across lineages?

L472: How was the estimate of 1.2 million generations derived? Please cite the relevant figure.

L476: Did starting from a distant genome size (110 Mb vs 1000+ Mb) help or hinder interpretability? Please comment on why this approach was necessary for A. psidii but not used for other species.

L501: Can the “recent contraction model” for A. psidii be further validated with data (e.g., TE age distributions, solo/integrity ratios, or comparative synteny)?

Figures:
Fig. 4C:
The p-values are shown as “0”, please report actual values or use a threshold notation (e.g., $p < 1e-10$).
Fig. S9, S10: Simulated vs original TE sequence diversity profiles differ substantially. If a real genome’s TE composition deviates from a normal distribution, does this impact simulation accuracy?

Fig. S11:
Axis text is very small and difficult to read, please improve readability.

(Remarks on code availability)

Reviewer #2

(Remarks to the Author)
Transposon/TE are mobile elements that largely affect the size of genome, especially for plants. Christopher, Ou, and colleagues developed a forward simulation tool PrinTE for TE dynamics. The advantage of this tool is its highly customizable parameters. The authors applied PrinTE to study several aspects of TE dynamics. They confirmed genome expansion is mainly due to LTR transpositions, and genome size is highly correlated with the number of solo-LTRs. The authors also emphasized that PrinTE can also be applied to study how genomes have been shaped. An exmaple is presented for A. psidii, and the results suggested its genome experienced a prolonged expansion followed by a short and ongoing contraction.
Overall, the manuscript is clearly written, and this work is solid and important for studying TE dynamics. Simulation tools are hard to benchmark, but I do feel like a more detailed benchmark is needed. Please see my other comments below.

1. When benchmarking LTR redetection (Fig. 2C), LTR_retriever was used. It requires the simulated LTR transposons have known features, such as TSD and motifs. Does PrinTE surpass the other two methods as it considers TSD in the simulation? If so, how do other methods perform without TSD as threshold? i.e. a benchmark similar to Fig. 2D.
2. line 367-371. Intact LTR reflects ongoing invasion and affects less on genome size. However, solo LTR or solo:intact indicates historic invasion, and I am not surprised (or say counter-intuitive) that its number is positively correlated with genome size, which is the consequence of long-term invasion. Could the authors discuss this?
3. I would recommend set the y-axis minimum to 0 for intuitive visualization if possible. For instance: Fig. 2E.
4. How does PrinTE’s speed/memory requirement compare to other methods?
5. Fig. 4: Assessing the effect of LTRs to genome size. Instead of using unsupervised PCA, I suggest linear regression, which could quantify the contribution of each LTR feature (soloLTR, solo:intact ratio etc.).

(Remarks on code availability)
I didn't test the code, but it is well-documented and contains all the details for installation and running.

Reviewer #3

(Remarks to the Author)
Benson et al. present a bioinformatics toolbox, named PrinTE, to model genome variation based on TE dynamics. The approach basically takes a genome containing a predefined number of genes and TEs, and models its evolution via a pre-defined transposition rate and TE elimination by illegitimate recombination, as well as mutation of TE sequences themselves. The program also allows more advanced parameters such as broad integration preferences and potential horizontal transfer, although the use and insights of these extended parametrizations are not presented. The authors applied PrinTE to Pucciniomycotina genomes and explored a number of parameter combinations to try to match genome size, LTR divergence distributions, and solo:intact ratios.

The tool could become a useful framework for hypothesis generation. However, the present analyses do not, in my view, support strong conclusions about the strength and relative contribution of forces shaping TE dynamics and genome evolution.

-The study relies on grid searches over insertion/deletion and a few additional parameters to reproduce selected summaries; however, there is no parameter identifiability, contribution, or uncertainty analysis, so fitted values read as effective averages over time rather than mechanistic estimates, and multiple parameter sets may explain the same summaries. A more formal inference layer (e.g., ABC, profile likelihood, fit surfaces) is needed.

-For the Pucciniomycotina case, which serves as a validation of the utility of PrinTE, the authors used a long TE expansion followed by a short contraction for A. psidii; however, these phase choices are imposed rather than inferred. So I am not sure what biological knowledge we can learn other than which parameters may fit the pre-defined, arbitrary constraints set. Moreover, the simulation achieves genome size and LTR divergence similar to the observed genome (which is somewhat trivial because the authors searched for such endpoints explicitly and tweaked the parameters in order to match these values). However, the simulations ended up with solo:intact ratios of ≈ 6.8 versus ≈ 23.5 observed. This contrast is left unresolved and likely reflects missing processes in the model (as noted by the authors). I believe that quantifying and attempting to close this mismatch should be part of the main results of the manuscript to go beyond the introduction of a bioinformatics tool for hypothesis generation.

-The paper concludes that a positive correlation between genome size and solo:intact suggests illegitimate recombination alone is insufficient to prevent genome expansion; however, this statement is descriptive and close to trivial unless placed in a quantitative framework that specifies the ranges of insertion, deletion, illegitimate recombination, and mutation rates that cannot hold size constant.

-Related to the previous point, LTR-RT removal is modelled via solo-LTR formation at a user-set rate and full deletions for non-LTR TEs; however, complete removals of neighbouring LTR-RTs by illegitimate recombination beyond solo formation are not explicitly parametrised, which can bias solo:intact expectations and age spectra.

-To my knowledge, PrinTE allows TE truncations and mutations, but does not model the origin and propagation of non-autonomous TEs with altered transposition rules. In fact, non-autonomous TEs typically outnumber autonomous counterparts and contribute disproportionately to TE content. Including an explicit module or at least sensitivity analyses of the emergence and contribution of non-autonomous TEs would be important.

-Population genetic forces such as drift, effective population size, mating system, admixture, polyploidy, and migration are discussed conceptually (implicitly or explicitly) but not implemented. These factors strongly affect TE load and fixation. I understand that including these parameters may be laborious and out of scope; however, claims should be framed as single-genome, panmictic approximations and not as estimates of force strengths. In other words, the modelling is interesting to generate hypotheses, but it is missing numerous crucial variables that can bias results and interpretations.

-Related to the previous point, comparisons to SLiM emphasise sequence-level modelling; however, a hybrid approach (SLiM for population genetics inference + PrinTE for sequence-level consequences) is not explored, and would partially address some of the concerns discussed above.

- It seems that the modelling of DNA transposons does not explicitly include cut-and-paste excision dynamics (excision rates, donor-site footprints/repair, coupling between excision and reinsertion, local hopping vs genome-wide reinsertion). Without an explicit excision module, DNA TE turnover and copy-number dynamics may be misrepresented. It would help to implement (or at least test the impact of ignoring such dynamics) an excision/reinsertion model and clarify the current assumptions used for DNA transposons.

(Remarks on code availability)

Version 1:

Reviewer comments:

Reviewer #1

(Remarks to the Author)
I find that all the risen concerns were overall properly addressed and that the new manuscript and amended figures are suitable for publication. I appreciate the in-depth revisions that have greatly improved the manuscript.

(Remarks on code availability)
I have not tested the code but the github page is clear with enough instructions for installing and running PrinTE.

Reviewer #2

(Remarks to the Author)

I thank the authors' effort to address my comments. I have no further concerns.

(Remarks on code availability)
Maybe i missed it, but adding simple test/demo data would be beneficial for users.

Reviewer #3

(Remarks to the Author)
Thank you for the detailed responses and for the substantial revisions. The implementation of broader parameter searches, fit landscapes, and sensitivity analyses clearly improves PrinTE as an informatic tool. However, I still have several concerns that I do not think have been fully addressed.

One specific issue concerns the modeling of DNA transposons. The revised implementation couples the excision of an intact Type II element to the reinsertion of an element from the same family, and the manuscript states that cut-and-paste transposons relocate while conserving copy number. Although excision is required for the mobilization of many DNA transposons, they do not necessarily conserve copy number. They can increase in copy number through mechanisms linked to DNA replication and repair. Otherwise, any non-zero deletion rate would lead to their progressive loss and eventual extinction, which is not consistent with the extensive accumulation of DNA transposons observed in many genomes. A strict one-to-one excision–reinsertion model is therefore not realistic. The model should allow the balance between excision and successful reinsertion or amplification to vary, or at least clearly acknowledge this limitation. The statement in Lines 172–173 that DNA transposons conserve copy number during mobilization should also be corrected or qualified.

I also remain unclear about how PrinTE handles the formation of new non-autonomous elements. The authors explain that users can control the initial abundance of autonomous and non-autonomous elements through the input TE library and TE-ratios table. However, this does not address my original concern, which was about the evolutionary emergence of new non-autonomous derivatives. Can an autonomous element lose its coding capacity during the simulation, become non-autonomous, and then propagate according to different mobilization rules? From the description of the model, TEs appear to accumulate sequence mutations and structural damage, but not to functionally evolve. If this is the case, it is an important limitation because the emergence and amplification of non-autonomous elements are major components of TE evolution.

The treatment of the solo:intact LTR ratio also remains inconsistent. In their response, the authors argue that this ratio is too affected by annotation uncertainty to serve as a reliable fitting criterion. However, the manuscript still uses the positive correlation between genome size and the solo:intact ratio to draw conclusions about the ability of recombination-mediated deletion to constrain genome expansion. If this metric is not reliable enough for model fitting, it should not be used to support strong biological conclusions. These interpretations should be removed, substantially toned down, or supported by independent analyses.

More fundamentally, I still do not clearly see what biological knowledge can be obtained from PrinTE beyond generating evolutionary narratives that are internally consistent with the model. The revised optimization procedure identifies parameter combinations that reproduce selected properties of an observed genome, such as genome size, TE copy number, cumulative TE length, and LTR-divergence distributions. This can show that a particular scenario is possible under the assumptions of PrinTE. However, it does not show that the selected parameters correspond to the real historical insertion, deletion, or recombination rates, or that the proposed scenario resembles what actually happened.

Very different evolutionary histories may produce similar present-day genome summaries. The manuscript does not show that PrinTE can distinguish between these alternative histories. For example, there is no parameter-recovery experiment in which genomes are generated using known but complex evolutionary scenarios, the true history is hidden, and PrinTE is then asked to recover the correct parameters and changes through time. Such an analysis would be important to show whether PrinTE can distinguish continuous activity from episodic bursts, changes in insertion rate from changes in deletion rate, or an expansion–contraction history from other processes that generate similar TE-age distributions. Showing that a parameter influences the composite score is not the same as showing that the parameter can be accurately recovered.

The inferred rates are also not compared with independent biological measurements, such as experimentally measured transposition rates, population-level TE insertion polymorphisms, pedigree or mutation-accumulation data, or independently dated TE bursts. Similarly, the model is not fitted using one set of genomic features and then tested on additional features that were not used for fitting. As a result, the case studies mainly show that the model can be adjusted to reproduce the same genome properties used to score the simulations. This is calibration, but it is not validation of the evolutionary history proposed by the model.

I therefore think the manuscript still overstates the biological meaning of the fitted parameters. Statements suggesting that PrinTE quantitatively estimates historical TE turnover rates, resolves genome-size evolution, demonstrates how expansion or contraction occurred, or uncovers the evolutionary trajectory of a lineage are not supported by the current analyses. At present, the outputs should be described more cautiously as parameter combinations that are compatible with selected present-day genome summaries under the assumptions of PrinTE.

PrinTE may be an interesting informatic tool for sequence-level simulation, benchmarking, and hypothesis generation. However, its ability to provide biological insight has not yet been clearly demonstrated. Unless the authors provide parameter-recovery tests, comparisons between genuinely different evolutionary scenarios, independent validation, or predictions of genomic features not used during fitting, statements claiming that the model “demonstrates, resolves, reveals,

or reconstructs particular evolutionary processes" should be removed or substantially toned down. In my opinion, the current analyses show that certain narratives can reproduce the observed data within the model, but they do not show that these narratives reflect biological reality.

(Remarks on code availability)
The code seems to be complete and documented, but I haven't tested.

Version 2:

Reviewer comments:

Reviewer #3

(Remarks to the Author)
The authors have addressed all the points raised with additional analyses and substantial revisions to the text to acknowledge the limitations of the study. The most important point, regarding the ability of PrinTE to recover real biological parameters, is now properly discussed in the manuscript. I agree with the authors that fully resolving this issue is beyond the scope of the current manuscript, but the additional analyses and discussion make this limitation and possible avenues to address it much clearer.

(Remarks on code availability)
The code is very complete and properly documented. I haven't test myself the program thought.

Open Access This Peer Review File is licensed under a Creative Commons Attribution 4.0 International License, which permits use, sharing, adaptation, distribution and reproduction in any medium or format, as long as you give appropriate credit to the original author(s) and the source, provide a link to the Creative Commons license, and indicate if changes were made.
In cases where reviewers are anonymous, credit should be given to 'Anonymous Referee' and the source.
The images or other third party material in this Peer Review File are included in the article's Creative Commons license, unless indicated otherwise in a credit line to the material. If material is not included in the article's Creative Commons license and your intended use is not permitted by statutory regulation or exceeds the permitted use, you will need to obtain permission directly from the copyright holder.
To view a copy of this license, visit https://creativecommons.org/licenses/by/4.0/

REVIEWER COMMENTS

Reviewer #1 (Remarks to the Author):

The authors present PrinTE, a forward-time simulator that operates on genome sequences to model TE insertions, deletions (including solo-LTR formation), truncations, and substitutions under fixed and variable-rate regimes. They benchmark its accuracy report good computational performance, and apply the framework to Pucciniomycotina fungi. Using simulations constrained by observed LTR divergence spectra, they infer contrasting TE histories for Cronartium ribicola (rapid turnover with near-stable size), Puccinia coronata f. sp. avenae (slow turnover), and Austropuccinia psidii (long expansion followed by a recent contraction). Across genomes, they find strong positive correlations between genome size and multiple LTR metrics, including a near-linear relation with solo-LTR count.

This manuscript introduces PrinTE, a new forward-time genome evolution simulator aimed at modeling TE dynamics, especially focusing on LTR retrotransposons. The authors provide a comprehensive study of rust fungi genomes and present PrinTE as a versatile tool for modeling genome size variation. While the manuscript is technically sound and provides rich simulation and case-study results, several conceptual clarifications, and broader validations beyond rust genomes are required to support the claims of generalizability and usability across different taxonomic groups.

Major Comments

Scope of Tool and Generalization Beyond Rust Fungi

• In the abstract the authors claim that PrinTE is versatile. If the tool is versatile, I expect it to work for non-rust fungi. If the case study to compelling and thouroughly investigated I think the authors need to demonstrate the efficacy of PrinTE on broader species (Arabidopsis for instance, since it’s used in the benchmarking). In addition, have the authors tested it on Ascomycetes with TE-rich genomes, such as Blumeria graminis? And what about fungal genomes with moderate TE content (20-30%)?

Response: We sincerely thank the reviewer for their insightful and constructive feedback, and we agree that our original manuscript did not adequately demonstrate the versatility of PrinTE in exploring the TE landscapes of a broad range of species. In our revised version, we have enhanced this by incorporating a use case in Arabidopsis thaliana. In total, we have tested PrinTE in genomes with varying TE contents across Kingdoms, including the TE-scarse Arabidopsis genome (15%), the Puccinia coronata

avenae genome with moderate TE content (31%), the Cronartium ribicola genome with higher TE content (42%), and the myrtle rust genome with extremely high TE content (67%). We demonstrated that PrinTE can be used to explore diverse TE evolutionary landscapes. Thus, we did not further apply PrinTE on Blumeria graminis that has a similar TE content to myrtle rust.

• Following the same train of thoughts – specifically inspired by this study case of fungal genome: How is Repeat-Induced Point (RIP) mutations would be handled by PrinTE? Can the authors comment of this? To support the claim of broad applicability, include validation or test cases beyond Pucciniomycotina, ideally from other fungal clades with different genome dynamics and TE regulation mechanisms (e.g., RIP-prone species).

Response: We thank the reviewer for raising the important point regarding Repeat-Induced Point (RIP) mutations, which are a defining feature of genome evolution in several fungal clades. RIP-prone species typically exhibit a strong transition bias (predominantly C:G to T:A), elevated mutational load in repetitive regions, and fragmentation or degeneration of LTR elements, all of which can result in faster deletions and tighter control of LTR dynamics. PrinTE is designed to simulate LTR insertions and deletions for studying their dynamic impacts on genomic evolution. Users can specify a higher mutation rate (-m parameter) and transition-to-transversion ratios (-TsTv parameter) to approximate the mutational signatures observed in RIP-affected genomes, so PrinTE simulation can faithfully represent these dynamics.

For users interested in isolating the RIP effect with the neutral substitution and degeneration of LTR elements, we recommend an alternative workflow where users apply established bioinformatic tools like RIPCAL (RIP Calculator) to perform alignment-based analysis of repeat families and calculate dinucleotide-based RIP indices (TpA/ApT and (CpA+TpG)/(ApC+GpT)) to identify RIP-affected sequences, followed by removal of the impacted LTR elements from the initial input library for PrinTE simulations. This approach enables exploratory analyses of how RIP-like mutation pressures influence TE dynamics without claiming mechanistic modeling of RIP itself. We also acknowledge that our original statement overstated the general applicability across taxonomic clades and have revised our manuscript accordingly.

• In general, I think the manuscript's background needs more information: I understand that the goal of this study is to present PrinTE but since the manuscript heavily focuses on rust fungi. It would benefit from explicitly stating this in the introduction and justifying the biological relevance as well as the challenges to infer TE dynamics in fungal genomes.

Response: We thank the reviewer for this valuable comment. We agree that the original manuscript placed a strong emphasis on fungal species, which may have limited the perceived general applicability of PrinTE. In response, we have added extra information in the introduction, expanded our analysis to include a plant genome (Arabidopsis), and have revised the Results section accordingly. In the updated version, we explicitly describe that rust fungi are used as a primary case study and clarify that PrinTE has been tested in additional species. These additions clarify both the motivation for focusing on rust fungi and the general utility of PrinTE across diverse eukaryotic genomes.

• The introduction also needs more about the host genome-TE arms race dynamics in terms of control mechanisms other than illegitimate recombination (IR). By the way IR is not even defined in the introduction. If that's an effective mechanism to remove LTRs, what about preventing insertions for instance by TE silencing and/inactivation? I think all these concepts need to be properly introduced for the reader to fully grasp what the study is testing.

Response: We agree that the previous version of the Introduction did not sufficiently frame our study within the broader context of the host genome-TE arms race. In the revised manuscript, we now explicitly introduce major host control mechanisms, including TE silencing and inactivation pathways that act to prevent or limit new insertions.

We also now define the recombination approach in the Introduction as a deletion mechanism that reduces TE length and genome size. Recombination is emphasized in our study because it alters the physical structure of TE insertions and thus has a direct and measurable effect on genome size evolution.

PrinTE does not explicitly model TE silencing or epigenetic inactivation mechanisms, as these processes primarily affect TE activity rather than the structural fate of existing insertions. Instead, their genome-level consequences are incorporated implicitly through the effective insertion and deletion rates. We have clarified this modeling rationale in the revised Introduction so that readers can better understand which aspects of the host-TE arms race are explicitly tested in this study and which are captured indirectly.

• Additionally, the manuscript would benefit from a brief discussion on how PrinTE and Kmer2LTR perform on smaller genomes, such as ~40 Mb, particularly in fungi with RIP signatures. Can these tools detect divergence accurately in highly mutated TE landscapes, or are there limitations?

Response: PrinTE and Kmer2LTR do not impose a lower limit on genome size, and both can be applied to small genomes (e.g., ~40 Mb). In our fungal analyses, we

restricted downstream analyses to genomes with >1% LTR content to ensure sufficient signal for comparative purposes; however, this was a study design choice rather than a limitation of PrinTE itself, which does not require a minimum TE abundance.

PrinTE explicitly records the insertion and deletion coordinates of simulated TEs independently of the underlying mutational landscape. Because PrinTE is a simulation and benchmarking framework rather than a TE detection method, its performance is not degraded by high mutation rates or RIP-like signatures. This makes it particularly well suited for evaluating TE detection tools in highly mutated TE landscapes, including those characteristic of fungi with RIP.

*By contrast, Kmer2LTR relies on sequence similarity and therefore has an upper limit of detectable divergence. Our benchmark shows that its detection range has substantially improved from ~25% (minimap2-based) to ~43% (Kmer2LTR) (**Fig. 2A**). We also stated this limitation more explicitly in the manuscript.*

Specific comments
Highly technical abstract: I find the abstract too technical and it is difficult to get the message of the study, what's the presentation of PrinTE and what are the outcome of the case study of its application in rust genomes. For instance, the inclusion of Pearson's r values in the abstract. This level of detail is too technical for an abstract.

Response: We thank the reviewer for this observation and agree that the original abstract was overly technical. We have revised the abstract to improve clarity and accessibility for a broader audience by focusing on the overall purpose and outcomes of the study, and by removing overly detailed statistical information (e.g., Pearson's r values). The revised abstract now more clearly presents PrinTE and summarizes the key outcomes of its application.

L61-71: The paragraph conflates whole-genome duplication (WGD) and transposable element (TE)-driven expansion, making it difficult to distinguish their respective roles in plant genome evolution. Please clarify when genome size increases are due to WGD versus TE activity, ideally by separating these concepts more clearly. Also, the cited example of Arabidopsis is misleading, the referenced work addresses genus-level variation, not within-species mechanisms, as A. lyrata has a larger genome than A. thaliana.

Response: We thank the reviewer for this suggestion. The distinction between whole-genome duplication, TE-driven expansion, and their respective impact on genome size is now stated more clearly in the Introduction. The Arabidopsis example has been revised to improve clarity.

L69: Why citing the commentary paper instead of the original research articles?

Response: Thank you for the suggestion. The citation has been revised to the original article of Hu et al. (2011) (PMID: 21478890) *rather than the commentary paper.*

L133: The manuscript states that TE insertions into intergenic space incur no cost, whereas insertions into genic space do. However, it is unclear how regulatory regions (e.g., promoters) are handled. Since TE insertions upstream of genes can affect expression, please clarify how regulatory elements are defined in the model. Is there a distance threshold from genes that determines "no cost" zones?

Response: You raise an important point. In the previous version of PrinTE, a gene was considered disrupted (i.e., incurring a fitness cost) only when a TE inserted directly into the gene body. We agree that this simplification does not fully reflect biological reality, as insertions into proximal regulatory regions can also alter gene expression and be subject to selection.

To address this, we have updated PrinTE to include a new parameter, '--promoter-boundary', which allows users to define a genomic window around genes that is treated as regulatory sequence. TE insertions occurring within this specified boundary are now assumed to incur the same fitness cost as insertions into the gene body. This effectively introduces a distance-based threshold from genes that distinguishes neutral intergenic regions from regulatory regions under selection.

We thank the reviewer for highlighting this limitation and helping improve the biological realism of the model.

L160-171: This section introduces the use of Kmer2LTR and SLiM to evaluate PrinTE's accuracy, but the methodology is currently unclear: How exactly was Kmer2LTR used to validate PrinTE's performance? Was PrinTE used to generate test genomes for Kmer2LTR, or was Kmer2LTR applied independently to real or SLiM-derived data? The manuscript mentions the Ti/Tv ratio in PrinTE but doesn't clearly explain how this relates to Kmer2LTR. Did the authors calibrate Kmer2LTR results using Ti/Tv, or was this parameter tested separately in PrinTE simulations? Regarding SLiM: Please clarify whether SLiM-simulated genomes were used to benchmark Kmer2LTR, to compare with PrinTE, or as control datasets. The workflow should be clearly structured in the text.

Response: We thank the reviewer for providing the feedback, and we agree that the original manuscript did not clearly describe the results. The benchmark using Kmer2LTR was designed to validate PrinTE's mutation and sequence identity

estimates. We have revised the manuscript to more clearly explain Kmer2LTR's function and how it was used throughout the analysis at L208 -216.

PrinTE simulates TE dynamics by inserting, fragmenting, and mutating transposable elements according to user-specified rules. During simulation, PrinTE explicitly tracks TE locations and structural states (e.g., intact versus fragmented) at each iteration, as well as the global, whole-genome mutational load. However, PrinTE does not record the exact genomic positions of individual nucleotide substitutions.

In contrast, Kmer2LTR operates directly on intact LTR retrotransposons. It aligns the two LTRs of each element and estimates genetic distance by quantifying nucleotide substitutions between the paired LTR sequences. In this study, Kmer2LTR was applied to intact LTR-RTs generated by PrinTE simulations to evaluate whether PrinTE's DNA substitution parameters, specifically the mutation rate (μ) and transition/transversion (Ti/Tv) ratio, produce the expected divergence patterns. Because PrinTE tracks the global mutation rate, we expect the LTR-LTR divergence measured by Kmer2LTR to reflect this underlying mutational distribution. Thus, Kmer2LTR serves as an independent validation of PrinTE's mutation models and of the correct sampling of the mutational distributions used during simulation.

Conversely, the use of simulated data allows us to assess Kmer2LTR's performance because agreement between PrinTE's known global mutation rates and Kmer2LTR's estimated LTR divergence is only possible if both tools are functioning as expected. Any discrepancy would result in incongruent estimates, providing a diagnostic signal of error in either method. Importantly, the Ti/Tv ratio was not used to calibrate Kmer2LTR; rather, it was specified within PrinTE simulations and subsequently evaluated through Kmer2LTR's K2P model.

SLiM simulations were used as an independent comparison framework. Genomes simulated with SLiM were compared directly to PrinTE simulations to contextualize PrinTE's behavior relative to an established population genetic simulator, rather than to benchmark Kmer2LTR itself. We have revised the text to explicitly state these roles and to present the overall workflow more clearly.

L212-219: It is unclear whether the 100 Mb genome simulation described here is the same one used to benchmark the performance and accuracy of PrinTE and Kmer2LTR in the earlier sections. Please clarify whether these simulations were part of the accuracy validation (e.g., Ti/Tv, divergence detection) or run independently for performance testing.

Response: The 100 Mb genome simulations used for performance benchmarking were independent of the simulations used for accuracy validation (e.g., Ti/Tv estimation and

divergence detection). These datasets were generated and analyzed separately for their respective purposes. The manuscript has been revised at L263 - 265 to clarify this distinction explicitly.

L228-229: Please specify the extent of the reduction in intact TEs. What was the initial TE coverage in the genome (e.g., % of genome or number of elements)? From Fig. S2A, it appears to be ~75% (please confirm). What exactly do “fragmented TEs” refer to in this context? Are these partial deletions, decayed sequences, or the result of nested insertions? Please clarify how they are defined and identified.
Response: The initial TE coverage of the genome was 80% (parameter -itp 80). In this study, “fragmented TEs” refer to TE-derived sequences that do not correspond to full-length elements and are not classified as solo-LTRs (which are treated as a separate category). These fragments arise through partial deletions of ancestral TEs and/or nested insertions. We have provided additional clarifications regarding the definition of fragmented TEs in L88 -89.

L231: Please specify that this second phase (elevated insertion rate) corresponds to the next 5 million generations to make the simulation timeline clear.
Response: Yes, we confirm the second phase corresponds to the next 5 million generations. The manuscript has been updated accordingly in L300 - 301.

L233: How many generations did it take for the genome to return to its initial size? From the figure, it appears this happens around ~9M generations; does the 4M generations qualify as “rapid”? If starting with only intact TEs at the genome size minimum, how many generations would it take to recover?
Response: By ~9 million generations, the chromosome has recovered to ~93% of its initial size. By 10 million generations, it exceeds the initial size, reaching ~143% (142,702,707 bp). Thus, recovery to approximately the initial genome size occurs between 9–10 million generations in this simulation.

We agree with the reviewer that the term “rapid” is subjective in this context. We have therefore removed this wording from the manuscript.

Under the fix-rate simulation method, if the simulation was initiated at the genome size minimum with only intact TEs present, the number of generations required to recover genome size would be roughly the same as shown in ***Fig. 3A–F****, because the insertion and deletion processes are not contingent on TE state. Variation may occur if the TE length profiles are different, since deleting a longer TE will have more impact on the genome size than deleting a shorter TE under the same deletion rate. We have revised the text at L302 to clarify these assumptions and their implications more explicitly.*

L235: “k>0”, Please define what the k parameter represents (it’s not introduced in Fig. S3). What k value was used in this scenario?

*Response: k is a user-defined parameter (--k) that controls the rate at which deletion probability increases with sequence length. This parameter biases deletions toward longer TE features by assigning higher deletion weights to sequences closer in length to the 95th percentile of TE length. In this simulation, a value of --k 3 was used for both the expansion and contraction phases, introducing a modest bias toward deleting longer TEs (**Fig. S1D**). The text has been edited at L276- 279 to clarify this point.*

L263-281: Consider moving this section on deletion parameters (rate, TE length bias, solo LTR rate) before the simulation results in Fig. 3. This would clarify the meaning and impact of the k parameter earlier for the reader.

*Response: Thank you for this suggestion. We have moved the section before the simulation results in **Fig. 3**.*

L285: Please specify which “molecular features” are being referred to (e.g., purifying selection)? population dynamics? TE competition? Clarification would help contextualize the variable-rate model.

Response: We agree and have improved the text to provide clarity. Here, “molecular features” refer to TE copy number, the proportion of intact versus fragmented elements, TE age and sequence divergence, and overall genome composition, including proximity to genes and chromatin state penalties.

L288: On what empirical data or rationale is the starting genome model of Arabidopsis thaliana based? Please clarify what aspects of the real genome this simulation reflects.

Response: The starting genome model is based on empirical data from Arabidopsis thaliana. Specifically, we used the observed genome size (~135 Mb) and chromosome number (5). The nucleotide substitution rate was set to 7 × 10^{-9} per site per generation, consistent with published estimates (Weng et al. 2019; PMID: 30514707)*. Gene number and gene sequences were taken from the TAIR10 protein dataset, including 19,621 coding genes. In addition, transposable elements were included to reflect their empirical abundance, comprising approximately 21% of the genome based on TAIR10. Together, these parameters ensure that the simulated genome captures key structural and evolutionary features of the A. thaliana genome. We also clarified these settings in the revised manuscript at L339-345.*

L290: Please specify what simulation conditions were tested (e.g., insertion/deletion parameters, mutation models, etc).

Response: We apologize for the lack of clarity regarding these details. Simulations were conducted under the following conditions. The chromosome number was set to 5 (-cn 5), and the genome size was set to 135 Mb (-sz 135Mb). The X-limit for the exponential decay function was set to 5 (-tmx 5), with a slope of exponential decay for sampling TE mutations during the burn-in of 2 (-tk 2).

The DNA substitution rate was set to 7 × 10^{-9} per site per generation (-m 7e-9). During the burn-in, 21% of the genome was filled by TEs, and 21.2% corresponded to TAIR10 CDS. The remainder of the genome was randomly generated sequences.

The TE birth rate (i.e., the rate of horizontally acquired TEs or newborn TE families) was set to 1 × 10^{-7} (-br 1e-7). TE insertion and deletion rates were set to 1.1 × 10^{-6} and 4 × 10^{-6} per intact TE per generation, respectively.

Chromatin bias for TE insertion and deletion was set to 1.1 and 1.0, respectively, resulting in a slight bias toward TE insertion in euchromatin (-cbi 1.1, -cbd 1.0). Euchromatin was defined as a 500 bp interval upstream and downstream of genes (-cb 500). The text has been revised at L1077-1080 to add clarity to these points.

L292: Why does the genome size decrease substantially more than it increases? Please comment on this asymmetry. Also, what is the TE landscape (e.g., ratio of solo/intact/fragmented TEs) when the genome size returns to baseline?

Response: Thank you for the insightful questions. The asymmetry between genome size increase and decrease arises from the way PrinTE couples TE accumulation with selection against gene disruption. Under the parameter settings used here (described in the previous comment and revised in text), TE insertions accumulate over time and increasingly disrupt genes. Gene-disrupting TEs are penalized in the model, which elevates the expected rate of TE excision events. As a result, once a sufficient burden of disrupted genes accumulates, TE deletions outpace insertions, leading to a net decrease in genome size.

At each simulation step, the number of TE excisions is sampled from a Poisson distribution whose mean (λ) increases with evolutionary time and with the cumulative weighted number of disrupted genes. The Poisson rate parameter (λ), plotted in ***Response Figure 1****, represents the expected number of TE deletions used to parameterize the stochastic sampling of excision events shown in* ***Fig. 3J*** *(solid orange line). Genome contraction therefore reflects an increase in the expected rate of TE*

deletion driven by selection against gene-disrupting insertions, rather than a symmetric balance between insertion and deletion processes.

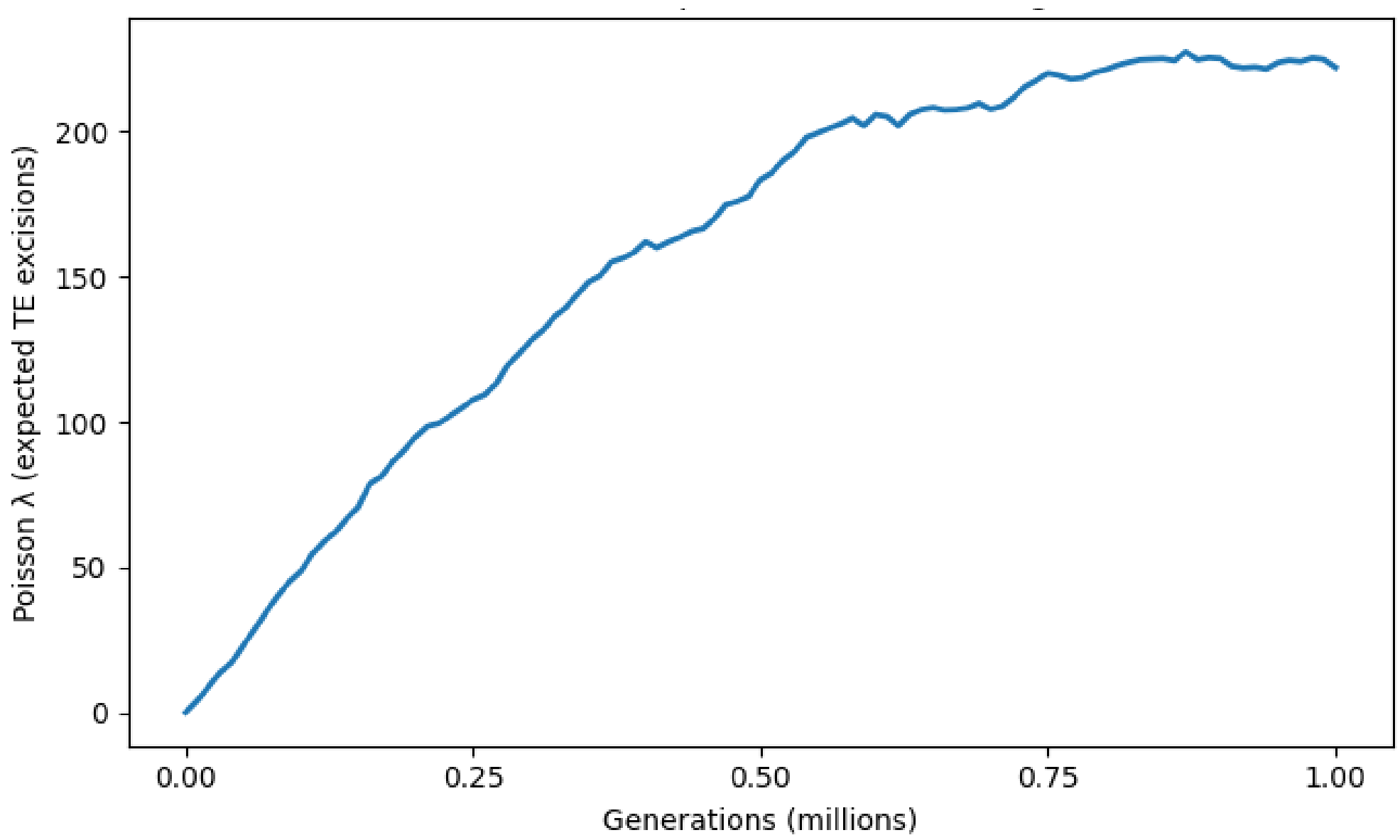

***Response Figure 1**: The Poisson rate parameter used to guide the sampling of deletion counts in PrinTE simulations associated with **Fig. 3 G-J**.*

At the 58,000-generation mark, the genome has roughly returned to baseline, measuring 99.98% of the original genome size. At this time point, the TE landscape consists of 10,360 intact TEs, 1,870 solo LTRs, and 2,167 fragmented TEs.

Section “Genome Size Evolution of Rust Fungi” This is a compelling case study, but to support claims of tool versatility, it would be important to test PrinTE on additional species (e.g., Arabidopsis or Ascomycetes). Also, how did the authors address: differences between short- and long-read assemblies, phased vs. unphased genomes?

Response: PrinTE operates at the genome-assembly level and is designed to model transposable element dynamics on haploid genomes or primary haplotypes, which can capture species-level evolutionary change over long timescales. In the rust fungi analyses, we explicitly removed residual alternative haplotypes prior to TE annotation and simulation to ensure that each genome represented a single haploid assembly.

To minimize potential biases arising from differences in genome assembly strategies, we controlled for assembly quality across species by using assemblies of comparable contiguity and completeness, and by applying a consistent TE annotation and filtering pipeline. Because PrinTE models TE evolution based on the assembled genome

sequence rather than raw reads, differences between short- and long-read sequencing technologies do not directly affect the simulations once a high-quality assembly is obtained. However, if a low-quality genome (i.e., most short-read genomes) is used for PrinTE simulation, because identical young TEs are collapsed in the genome, the TE identity profile is shifted to higher divergence and lacks recent TE activities. As a result, the insertion parameter will be underestimated, and the deletion parameter overestimated, both not representing the species.

We have revised the manuscript at L1127-1132 to clarify that PrinTE is intended for use with haploid genomes or primary haplotypes. In addition, following the reviewer's suggestion, we have added simulations using Arabidopsis thaliana to demonstrate the versatility of PrinTE beyond rust fungi and to show its applicability to phylogenetically distant taxa.

We thank the reviewer for this valuable suggestion, which has helped us improve both the clarity and the scope of the manuscript.

L367-369: Is this truly counter-intuitive? Fig. S6 suggests that high retention of solo LTRs (e.g., solo = 95%) slows down purging and allows for larger genomes. This would explain the correlation. Additionally, the solo/intact ratio may not accurately reflect purging "intensity", it could also result from a burst-purge cycle. Please consider qualifying this interpretation.

Response: We thank the reviewer for the valuable insight. The solo:intact LTR ratio is commonly used as a proxy for LTR-RT purge pressure, with higher ratios interpreted as more intense conversion of intact LTR-RTs into solo-LTRs. In this framework, the intact LTR-RT is substantially longer than a solo-LTR; thus, higher solo:intact ratios are generally expected to be associated with smaller genome sizes.

Within the PrinTE framework, transposable elements are randomly selected for deletion (parameterized by k and related weighting terms). Deletions are partitioned into (i) full-length deletions, (ii) partial deletions that generate TE fragments, and (iii) partial deletions of LTR-RTs that generate solo-LTRs. Consequently, the alternative to forming a solo-LTR in the model is not retention of an intact element, but rather its complete removal or other form of partial deletion. Under this modeling assumption, increasing the rate of solo-LTR formation results in larger genomes. ***Fig. S6*** *illustrates this expected behavior and serves primarily as a benchmarking figure demonstrating that PrinTE parameters produce genome size dynamics consistent with these assumptions.*

The positive correlation we observe between genome size and the solo:intact LTR ratio, however, is a biological pattern derived from TE annotations in rust fungi, not a direct output of the simulation framework. We therefore agree with the reviewer that this

observation should be interpreted cautiously. In particular, the solo:intact ratio may not solely reflect the intensity of purging, but could also arise from alternative evolutionary scenarios, such as the different phases of burst–purge cycles or differential retention of solo-LTRs following periods of high LTR-RT activity.

Accordingly, we now modify our interpretation and emphasize that the solo:intact LTR ratio is an imperfect proxy for purge pressure, especially when inferred from real genomes where annotation quality, TE age structure, and lineage-specific dynamics can influence the observed ratio. Continued improvements in LTR-RT and solo-LTR annotation methods will be essential for refining these inferences, and we suggest that simulation frameworks such as PrinTE may be useful for benchmarking such tools. Our rust fungi simulations highlight the plausibility of alternative interpretations and motivate further investigation into the evolutionary processes underlying this correlation. Based on these discussions, we have rewritten the relevant parts in the abstract and main text (L423-439).

L385: "LTR-RT insertion passed the current time" Please clarify what is meant by this phrase. Does it imply a decline in recent insertions, or that the peak insertion activity lies in the past?

Response: We apologize for the confusion. We mean the highest peak of the LTR-RT divergence landscape is larger than 0 (current), suggesting a decline in recent (young; lowly diverged) insertions. The text has been edited to clarify this point. L447-448

L403-406: This contrast between high-integrity and fragmented TE landscapes is particularly interesting. Despite distinct TE dynamics modes, both species exhibit genome size stability? Could the authors elaborate on how different strategies may converge on similar genome size maintainance?

*Response: We thank the reviewer for these questions. Both Cronartium ribicola and Puccinia coronata f. sp. avenae exhibit genome size stability in a phylogenetic context (**Fig. 4A**), despite markedly different LTR-RT landscape characteristics. In C. ribicola, LTR-RTs display high integrity (i.e., largely intact and potentially active; **Fig. S9D**) and a young age distribution (**Fig. 4D**). Although this genome contains many intact LTR-RTs, its overall genome size is comparable to other Pucciniomycetes (middle orange dot in **Response Figure 2**), indicating that recent TE activity has not resulted in genome expansion. This pattern is consistent with a high-insertion-high-deletion dynamic, in which active TE proliferation is counterbalanced by efficient TE removal or deletion.*

*In contrast, the P. coronata f. sp. avenae genome shows low LTR-RT integrity (i.e., fragmented and likely inactive elements; **Fig. S10D**) and an older LTR-RT age distribution (**Fig. 4D**). Its genome size is likewise similar to that of other*

*Pucciniomycetes (lower orange dot in **Response Figure 2**), suggesting a low-activity regime characterized by both limited TE insertion and limited TE removal.*

Together, these results illustrate how distinct TE strategies (rapid turnover of intact elements versus long-term persistence of fragmented, inactive elements) can converge on similar genome size outcomes. We note that these three fungal genomes were deliberately selected to span a broad range of LTR-RT profiles, representing extremes within the Pucciniomycetes, in order to highlight the diversity of TE "fingerprints" that PrinTE is able to model.

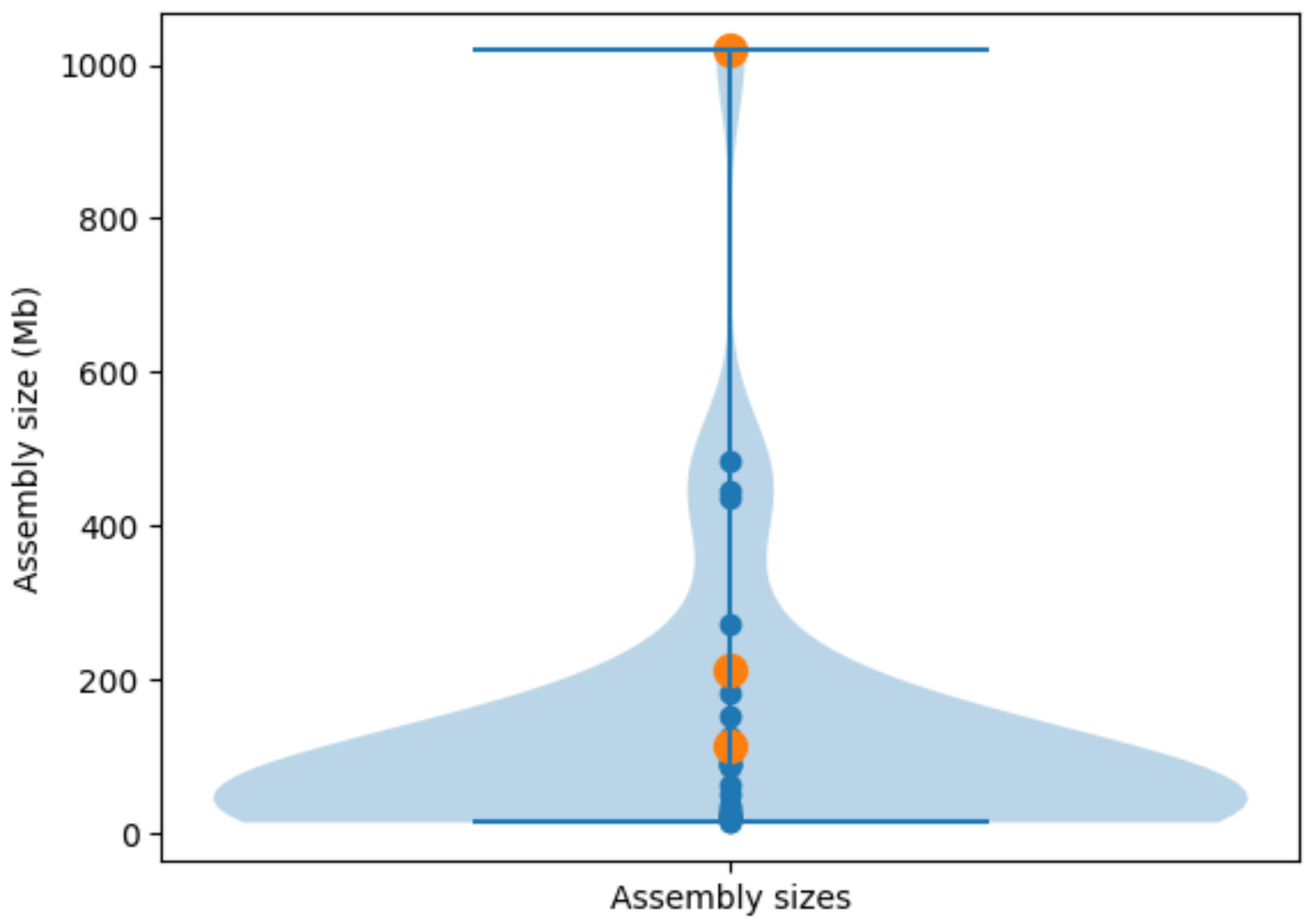

***Response Figure 2**: Violin plot of genome assembly sizes for the Pucciniomycetes genomes used in this study (**Fig 4. A-C**). Orange genomes are those that were selected for forward simulation with PrinTE.*

L438-439: The simulation assumes constant TE insertion and deletion rates? Is this assumption evolutionary realistic? Please discuss its limitations or relevance over evolutionary timescales.

Response: This is an important point and highlights a key simplifying assumption of PrinTE and of forward simulations more generally. Yes, the current simulation framework assumes constant TE insertion and deletion rates within a given run. From an evolutionary perspective, this assumption is clearly an approximation, as TE activity is known to vary over time due to changes in population size, host regulatory mechanisms, environmental stress, and episodic bursts of transposition.

The relevance of this assumption therefore depends on the evolutionary timescale being modeled. PrinTE is designed to efficiently compress many generations into iterative steps, allowing users to explore long-term TE dynamics without prohibitive computational costs. This strength also represents a limitation. When multiple

generations are summarized within a single iteration, temporal variation in insertion and deletion rates within that interval is necessarily averaged. Thus, the insertion and deletion rates modeled in PrinTE are effective insertion and deletion rates.

Importantly, this limitation can be mitigated in several ways. First, the number of generations per iteration is fully customizable, allowing users to simulate short timescales (down to a single generation) when fine-grained dynamics are of interest. Second, PrinTE supports "pick up where left off" execution, enabling users to modify insertion and deletion rates between runs and continue the simulation from an intermediate state. This allows rate heterogeneity to be introduced across evolutionary time, rather than enforcing strictly constant rates.

As a result, while individual simulation segments assume constant rates, users can model changing TE dynamics by stepping through successive evolutionary intervals with different parameter values.

L458-458: Why was the "burn-in" simulation from 110 Mb only done for A. psidii and not for C. ribicola or P. coronata? Could the authors simulate a hypothetical common ancestral "rust fungus" genome for comparative trajectories across lineages?

Response: We thank the reviewer for the great suggestion. We originally aimed to highlight PrinTE's flexibility both as a complementary framework for existing TE simulators (i.e., TEgenomeSimulator) and as a stand-alone TE simulation tool. We understand this can be confusing and thus took your advice to simulate hypothetical ancestral genomes as "burn-in" for the three fungal genome studies. We then used PrinTE to forward evolve these ancestral genomes, targeting to reach TE landscapes that mimic the modern-day reference genomes. To find the best parameter combinations that reproduce the reference TE landscape, we developed a brand-new component in PrinTE that can perform automated parameter sweeping and evaluation, which can reproducibly find the best match parameters. Using this revised approach, we re-simulate the three fungal genomes to estimate TE dynamic parameters that can produce genomes similar to the observed references. Please refer to the brand-new ***Fig. 6*** *and the corresponding text for more details.*

Additionally, we demonstrate the use of a hypothetical common ancestral genome as the "burn-in" in our Arabidopsis simulations. Together, the revised manuscript and PrinTE package provide a reproducible framework that can be readily adapted for similar comparative evolutionary analyses in eukaryotic genomes. We thank the reviewer for highlighting this possibility and for their constructive feedback, which helps clarify and strengthen the presentation of PrinTE's capabilities.

L472: How was the estimate of 1.2 million generations derived? Please cite the relevant figure.

Response: We estimated ~1.2 million generations from the observed peak of the LTR divergence distribution (Figure below). In Basidiomycota (including the Pucciniomycetes), mutation and substitution rates vary among lineages and genomic regions, but published values generally fall within the range reported for other fungi. We therefore applied a nucleotide substitution rate of 2 × 10^{-9} substitutions per site per generation, consistent with current estimates for Basidiomycete fungi (Kasuga et al. 2002 PMID: 12446823*; Ene et al. 2018* PMID: 30150418*).*

Using the peak LTR divergence value of 0.00552366 (Figure below), the corresponding number of generations was calculated as:

*Generations = 0.00552366 / (2 * (2 × 10^{-9})) ≈ 1,380,915*

Thus, the divergence peak corresponds to approximately 1.4 million generations from the present. In the revised manuscript, we simulated the second phase of A. psidii genome evolution for 1.4 million generations.

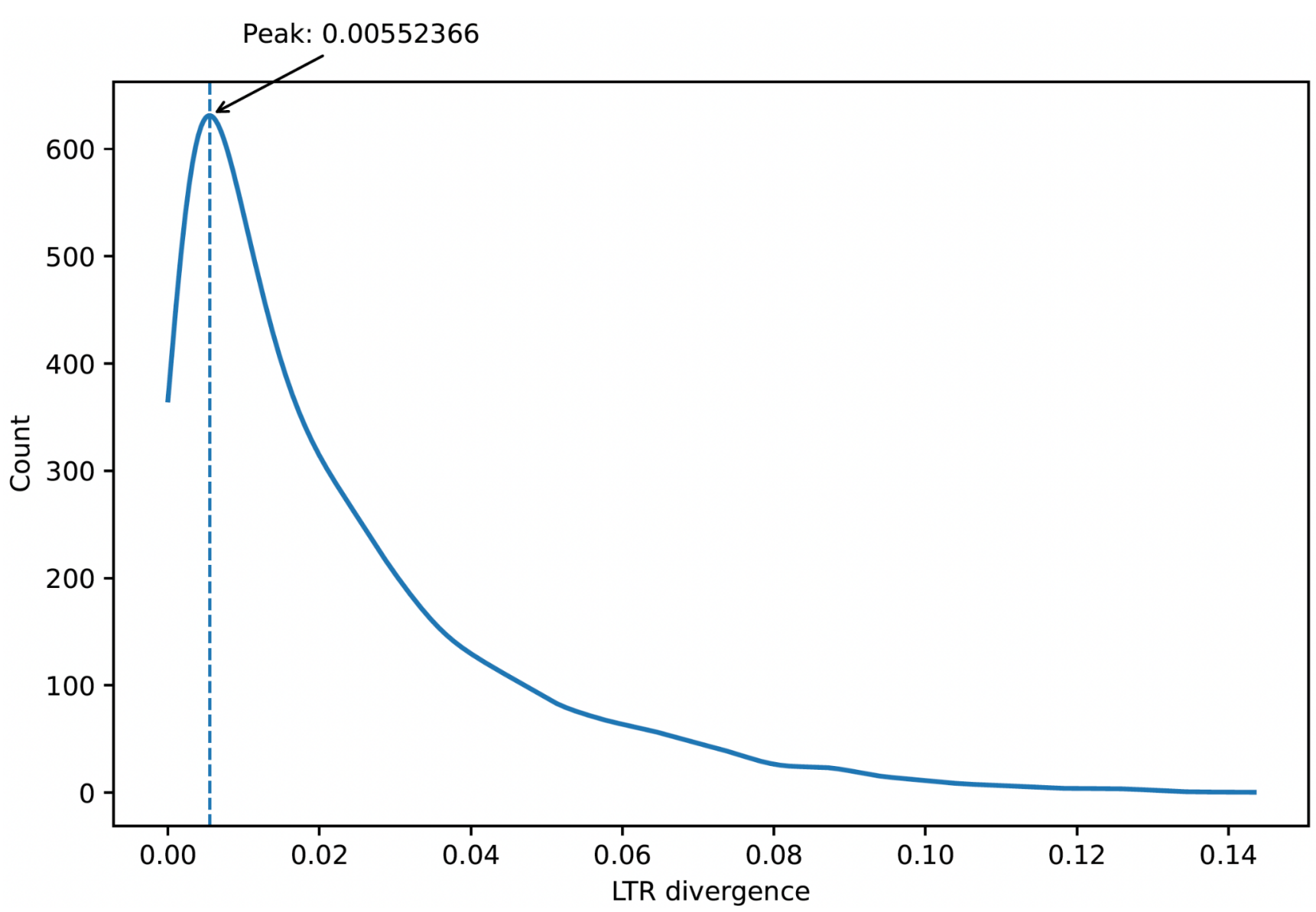

***Response Figure 3**: LTR-RT density distribution for Austropuccinia psidii (myrtle rust) as shown in **Fig 4D**, including the LTR divergence value associated with peak density.*

L476: Did starting from a distant genome size (110 Mb vs 1000+ Mb) help or hinder interpretability? Please comment on why this approach was necessary for A. psidii but not used for other species.

Response:

Response: We appreciate the reviewer's question about the methodological inconsistency in our original submission. We have addressed this concern by implementing a unified ancestral-state reconstruction approach across all three species. In the revision, A. psidii, P. coronata f. sp. avenae, and C. ribicola are now treated identically. Rather than starting from a present-day genome, each species is anchored in a phylogenetically reconstructed most recent common ancestor (MRCA), evolved forward under wide-ranging parameter combinations, and scored against the observed present-day genome. We thank the reviewer for helping to improve PrinTE.

L501: Can the "recent contraction model" for A. psidii be further validated with data (e.g., TE age distributions, solo/integrity ratios, or comparative synteny)?
Response:

*Response: Comparative synteny analyses at this resolution are currently not feasible for A. psidii. The species is highly divergent from other rust genomes since it is a monotypic genus of rust (**Fig. 4A**). Although the assemblies chosen for downstream analyses are of high quality, they are not resolved at the chromosome level. As a result, syntenic comparisons are weak and fragmented. For example, **Response Figure 4** shows synteny between A. psidii and P. coronata (one of its closest available relatives) and depicts only limited, discontinuous syntenic signal between the longest 15 scaffolds of each genome, which is consistent with substantial genome divergence and rearrangement.*

Regarding additional validation, we used TE age distribution analyses as our primary line of evidence supporting the recent contraction model (L958-971). The simulation-estimated best parameters yielded the lowest Composite Distance Metric, suggesting their optimum estimation. Because the estimation method is robust and reproducible, we believe it is more credible than random parameter combinations. We have edited the manuscript text to clarify these points.

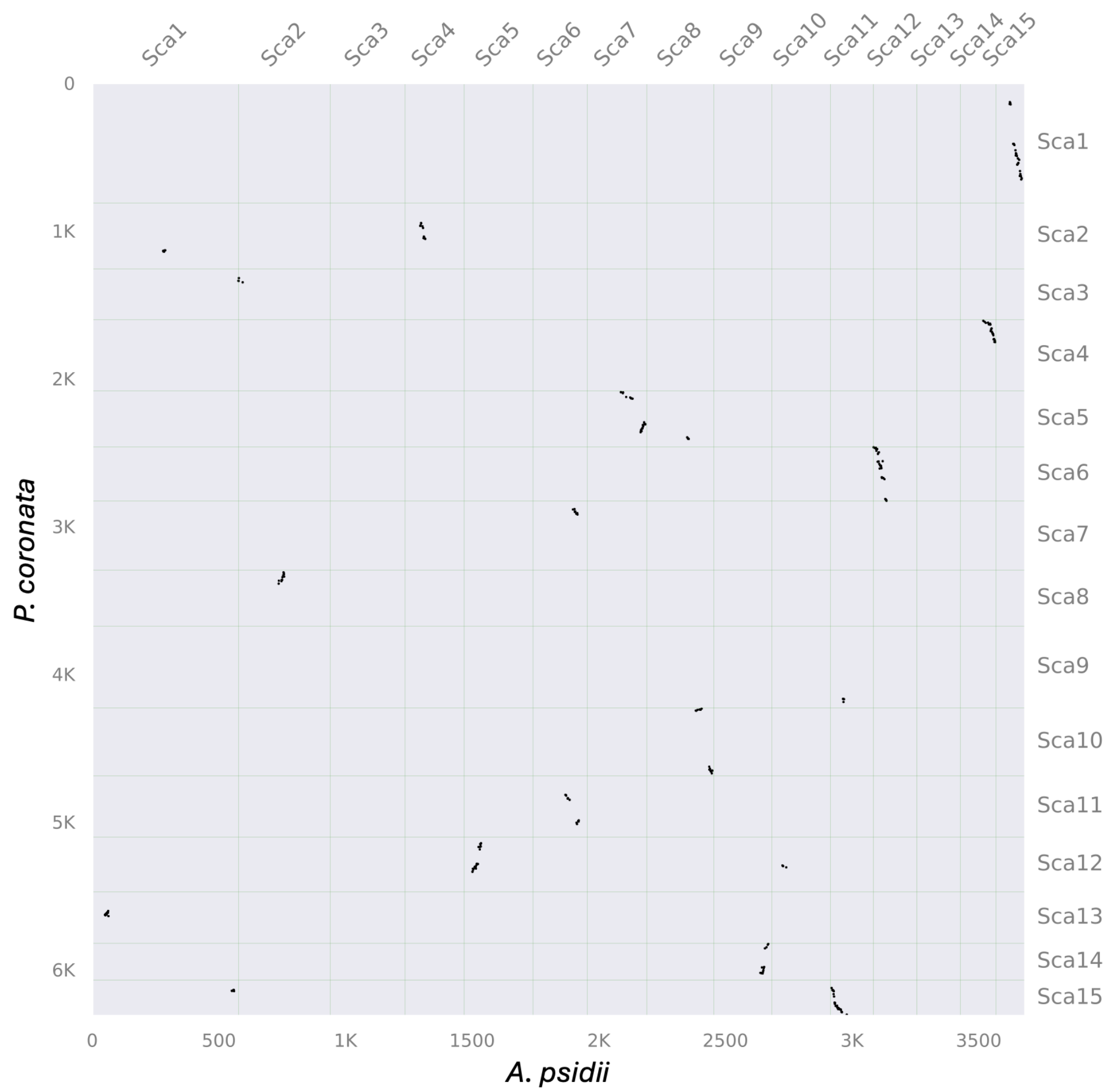

***Response Figure 4**: Syntenic dotplot shows sparse and disjointed macrosynteny between 15 largest scaffolds of Austropuccinia psidii and one of its nearest neighbors, Puccinia coronata f. sp. avenae, reflecting their distant evolutionary relationship.*

Figures:
Fig. 4C:
The p-values are shown as “0”, please report actual values or use a threshold notation (e.g., p < 1e-10).

Response: Thanks for the suggestion. We have replaced p-values with scientific notation.

Fig. S9, S10: Simulated vs original TE sequence diversity profiles differ substantially. If a real genome’s TE composition deviates from a normal distribution, does this impact simulation accuracy?

Response: TEgenomeSimulator approximates the sequence identity distribution of TEs using a Gaussian distribution parameterized from the observed TE sequence identities in the real genome. A Gaussian distribution provides a reasonable approximation of overall TE integrity patterns.

By design, the simulated and empirical distributions are not expected to match perfectly, particularly for genomes whose TE sequence identities deviate substantially from a normal distribution. However, in TEgenomeSimulator, the Gaussian distribution is used only to guide the accumulation of point mutations within TE copies. It does not influence indel generation and therefore does not directly affect genome size or analyses focused on genome size evolution.

Fig. S11:
Axis text is very small and difficult to read, please improve readability.

Response: Thank you for pointing this out. After integrating the parameter sweep module thanks to the reviewer's feedback, this figure is no longer helpful and has been removed from the updated manuscript

Reviewer #2 (Remarks to the Author):

Transposon/TE are mobile elements that largely affect the size of genome, especially for plants. Christopher, Ou, and colleagues developed a forward simulation tool PrinTE for TE dynamics. The advantage of this tool is its highly customizable parameters. The authors applied PrinTE to study several aspects of TE dynamics. They confirmed genome expansion is mainly due to LTR transpositions, and genome size is highly correlated with the number of solo-LTRs. The authors also emphasized that PrinTE can also be applied to study how genomes have been shaped. An exmaple is presented for A. psidii, and the results suggested its genome experienced a prolonged expansion followed by a short and ongoing contraction.
Overall, the manuscript is clearly written, and this work is solid and important for studying TE dynamics. Simulation tools are hard to benchmark, but I do feel like a more detailed benchmark is needed. Please see my other comments below.

1. When benchmarking LTR redetection (Fig. 2C), LTR_retriever was used. It requires the simulated LTR transposons have known features, such as TSD and motifs. Does PrinTE surpass the other two methods as it considers TSD in the simulation? If so, how do other methods perform without TSD as threshold? i.e. a benchmark similar to Fig. 2D.

*Response: We thank the reviewer for the constructive suggestion. PrinTE was specifically designed to model as many biologically meaningful TE hallmarks as possible, including TSDs. The benchmark in **Fig. 2C** therefore highlights the benefit of explicitly simulating these structural features, which are required by downstream tools such as LTR_retriever.*

*Importantly, LTR_retriever does not provide an option to disable TSD-based filtering, making it impossible to directly reproduce a **Fig. 2D**-style benchmark with the same pipeline. To address the reviewer's suggestion, we therefore performed an additional benchmark (**Response Figure 5**) using the raw predictions from LTRharvest and LTR_FINDER, both of which allow TSD detection to be disabled. This enables an evaluation analogous to Fig. 2D that does not rely on TSDs as a threshold.*

When TSD detection is disabled, LTR-RT redetection rates approach ~1.0 for genomes simulated with PrinTE (with or without TSDs) and SimulaTE. This indicates that under permissive detection conditions, the majority of simulated LTR-RT insertions are recoverable regardless of whether TSDs were explicitly modeled.

In contrast, genomes simulated with ReplicaTE show an anomalously high apparent detection rate, indicating substantial overprediction. This is not due to improved recovery, but rather reflects a large number of false-positive LTR calls produced by LTRharvest/LTR_FINDER when applied without stringent post-processing. ReplicaTE constructs artificial genomes by assembling fragments of genes, annotated TEs, and intergenic regions extracted from a real genome. As a result, the simulated sequences can contain repetitive or tandem structures that resemble LTR architectures and are misidentified as new LTR-RTs under relaxed settings.

*These results reinforce the importance of LTR-specific cleanup pipelines such as LTR_retriever and clarify that PrinTE's advantage in **Fig. 2C** primarily arises when realistic TE structural hallmarks (including TSDs) are required by the benchmarking tool, whereas relaxing these constraints leads to near-complete recovery across simulators except in cases where genome construction introduces spurious repetitive patterns (as observed for ReplicaTE).*

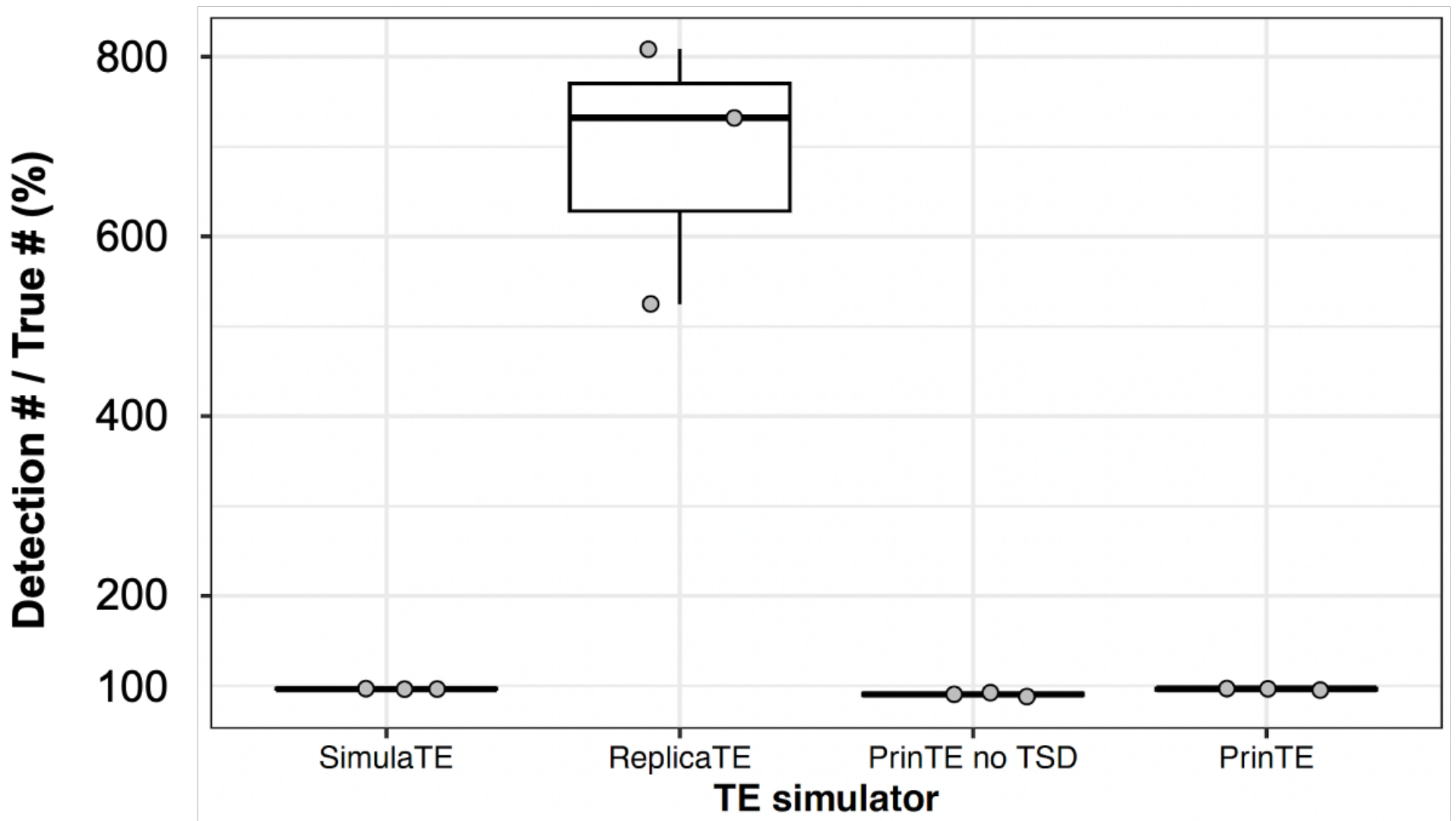

***Response Figure 5**: LTR-RT recall rate using LTR_harvest and LTRfinder with TSD-related parameters deactivated. LTR-RT detection rates higher than 100% indicate false positives. Each dot represents one replication.*

2. line 367-371. Intact LTR reflects ongoing invasion and affects less on genome size. However, solo LTR or solo:intact indicates historic invasion, and I am not surprised (or say counter-intuitive) that its number is positively correlated with genome size, which is the consequence of long-term invasion. Could the authors discuss this?

Response: We thank the reviewer for the insightful input. The solo:intact LTR ratio is widely used as an indicator of the intensity of LTR retrotransposon removal (purging).However, the relationship between solo:intact ratios and genome size evolution is likely more complex. As the reviewer points out, an accumulation of solo-LTRs may also represent the long-term outcome of historical waves of TE invasion, such that genomes experiencing sustained retrotransposon activity over evolutionary time could retain large numbers of solo elements while still expanding overall. Thus, the positive correlation we observe may indeed reflect prolonged TE turnover rather than solely inefficient removal.

We agree that this alternative interpretation is scientifically plausible and our data are consistent with it. We have revised the text in this section to better acknowledge these nuances and to clarify that the association between solo:intact ratios and genome size remains an evolving area requiring further investigation. L423-439.

3. I would recommend set the y-axis minimum to 0 for intuitive visualization if possible. For instance: Fig. 2E.

*Response: We thank the reviewer's careful reading of the manuscript. We have set the y-axis minimum of **Fig. 2E** to 0.*

4. How does PrinTE's speed/memory requirement compare to other methods?

*Response: We thank the reviewer for this good suggestion. We want to clarify that Fig. 2E measures the timing of PrinTE's core forward-simulation functionality, which models TE evolution over time. ReplicaTE and SimulaTE are single-timepoint simulators and do not support forward evolutionary dynamics. For the comparisons shown in **Fig. 2C–D**, PrinTE was run in a restricted single-timepoint mode using the --burnin_only option. We benchmarked the speed/memory of PrinTE against other simulators in the single-timepoint mode. ReplicaTE is quite fast at smaller scales, which could simulate 15.5 Mb of genomic sequence in 14 seconds using 0.44 GB of memory. In comparison, PrinTE simulated a much larger 508 Mb sequence in 2 minutes while using 5.45 GB of memory, demonstrating that it remains efficient when scaling to hundreds of megabases. SimulaTE simulated an even larger genome (1,020 Mb) in 17 minutes using 1.2 GB of memory. PrinTE was run with 200 threads, taking advantage of its native multithreading, whereas ReplicaTE and SimulaTE provide no parallelization option and are limited to a single core. It is important to note that this is not a strictly apples-to-apples comparison, since these simulators differ in scope and functionality.*

Overall, while these benchmarks should be interpreted cautiously, they suggest that PrinTE achieves strong performance at substantially larger simulation scales, making it well-suited for realistic, genome-sized TE simulation tasks.

5. Fig. 4: Assessing the effect of LTRs to genome size. Instead of using unsupervised PCA, I suggest linear regression, which could quantify the contribution of each LTR feature (soloLTR, solo:intact ratio etc.).

Response: We thank the reviewer for this constructive suggestion. In response, we have performed linear regression analyses to complement the original PCA, allowing us to explicitly evaluate the effects of features such as soloLTR and solo:intact ratio. We test the following multivariate regression:

GenomeMB ~ LTR_percent + Solo.count + Intact.count + Solo.Intact.ratio

Coefficients:

	Estimate	Std. Error	t value	Pr(>|t|)
(Intercept)	47.1146697	14.2645360	3.303	0.0042 **
LTR_percent	1.6477453	0.9740302	1.692	0.1090
Solo.count	0.0041453	0.0003151	13.155	2.44e-10 ***
Intact.count	-0.0002848	0.0072252	-0.039	0.9690

Solo.Intact.ratio	*1.6147834*	*2.0016546*	*0.807*	*0.4310*

The regression found the Solo.count variable is the only significant factor (p = 2.44e-10), confirming our previous discussion on the relationship between genome size, solo count, and solo:intact ratio. The new result is now included in the updated manuscript.

Reviewer #2 (Remarks on code availability):

I didn't test the code, but it is well-documented and contains all the details for installation and running.

Response: We thank the reviewer for confirming our tool's documentation and usage. We further improved the documentation and provided more use case examples to reflect the substantial revision of the tool.

Reviewer #3 (Remarks to the Author):

Benson et al. present a bioinformatics toolbox, named PrinTE, to model genome variation based on TE dynamics. The approach basically takes a genome containing a predefined number of genes and TEs, and models its evolution via a pre-defined transposition rate and TE elimination by illegitimate recombination, as well as mutation of TE sequences themselves. The program also allows more advanced parameters such as broad integration preferences and potential horizontal transfer, although the use and insights of these extended parametrizations are not presented. The authors applied PrinTE to Pucciniomycotina genomes and explored a number of parameter combinations to try to match genome size, LTR divergence distributions, and solo:intact ratios.

The tool could become a useful framework for hypothesis generation. However, the present analyses do not, in my view, support strong conclusions about the strength and relative contribution of forces shaping TE dynamics and genome evolution.
Response: We thank the reviewer for this important perspective. In direct response to this concern, we have substantially revised the analytical workflow aiming to provide a more reproducible approach to quantify the hypothetical evolutionary scenarios. Rather than simulating from narrow and subjective parameter values, PrinTE now uses ancestrally reconstructed genome size and TE states to define a more empirically grounded starting point. Simulations are then run forward from this inferred ancestral state to the present, and terminal simulations are evaluated against the corresponding empirical genome. We further implemented broad grid searches spanning orders of

magnitude in parameter space, together with a Composite Distance Metric that allows thousands of simulations to be performed and evaluated automatically. This provides a more quantitative and reproducible way to identify parameter combinations that best recreate the observed genome size and TE composition. These changes represent a substantial revision of the PrinTE workflow and directly address the reviewer's concern by better anchoring simulations to empirical data while avoiding overstating causal force strengths.

-The study relies on grid searches over insertion/deletion and a few additional parameters to reproduce selected summaries; however, there is no parameter identifiability, contribution, or uncertainty analysis, so fitted values read as effective averages over time rather than mechanistic estimates, and multiple parameter sets may explain the same summaries. A more formal inference layer (e.g., ABC, profile likelihood, fit surfaces) is needed.

Response: We thank the reviewer for this important suggestion. We agree that the original version of the manuscript overemphasized single best-fitting parameter combinations and did not sufficiently evaluate alternative parameter sets. In this revision, we provide a more reproducible and robust approach to estimate TE dynamic parameters that help support the TE-genome size evolution hypothesis, and to also evaluate the uncertainty of a single parameter set and the alternatives that explain the same summaries equally well.

*To achieve this goal, we have added a new parameter-evaluation layer to the PrinTE pipeline. Instead of reporting only the best grid-search result, the revised pipeline now compares each simulated genome to the reference genome using several genome-scale summaries: the number of detected LTR retrotransposons, their cumulative genomic length, total genome size, and the distribution of LTR-RT divergence. The pipeline then calculates a weighted composite score to evaluate the distances of these parameters between the simulated genome and the reference genome, allowing us to visualize not only the best-fitting point but also the broader region of parameter space that produces similar genomic summaries. This allows us to generate fit surfaces and assess whether the data support a narrow optimum or a broader set of nearly equivalent solutions (**Fig. 6**).*

In the revised manuscript, we now present these results as fit landscapes rather than as single fitted values. We have therefore revised the language throughout the text to describe PrinTE outputs as effective parameter sets or compatible parameter ranges, not direct estimates of historical rates due to the lack of ancient genomes.

We also added a contribution analysis by retaining the individual components of the composite score. This allows us to ask which biological features drive the fit. For

example, whether a parameter set is favored mainly because it reproduces genome size, because it captures the LTR-divergence distribution, or because it matches TE copy number and cumulative TE content. Below is an example output of this analysis. In this example, the insertion_rate and deletion_rate contribute more to the simulation than solo_rate and length_bias. Insertion rate alone explains 91% of the variance in the composite score, while deletion rate explains 35%.

```
=== Sensitivity summary – RandomForest, cross-validated (full dataset
– unfiltered) ===
  Excluded 578 imputed/failed sim(s); using 222 real simulations.
    Importances are permutation-based (mean drop in R² when a feature
    is shuffled) and describe the *explored* region, which the
adaptive
    search concentrated near the optimum — not the global space.
  Joint R² (5-fold CV):  0.8241  (+/- 0.1902)
  Joint R² (OOB):        0.9093
  (n=222, p=4)
  Per-parameter sensitivity (ranked by permutation importance):
    parameter                  perm. importance    solo CV R²
    log10(insertion_rate)   0.6475 +/- 0.0394        0.9121
    log10(deletion_rate)    0.3988 +/- 0.0284        0.3462
    solo_rate               0.0034 +/- 0.0015       -0.4045
    length_bias             0.0024 +/- 0.0004       -0.3505
```

We have clarified that PrinTE is currently intended as a hypothesis-generating simulation framework, not a full statistical inference engine. In the revised version, we now describe the new fit-surface analysis as an intermediate inference layer that addresses the reviewer's central concern by evaluating whether fitted parameter values are unique (L946-948), whether multiple parameter combinations are similarly compatible with the data, and which genomic summaries contribute most strongly to the observed fit.

-For the Pucciniomycotina case, which serves as a validation of the utility of PrinTE, the authors used a long TE expansion followed by a short contraction for A. psidii; however, these phase choices are imposed rather than inferred. So I am not sure what biological knowledge we can learn other than which parameters may fit the pre-defined, arbitrary constraints set. Moreover, the simulation achieves genome size and LTR divergence similar to the observed genome (which is somewhat trivial because the authors

searched for such endpoints explicitly and tweaked the parameters in order to match these values). However, the simulations ended up with solo:intact ratios of ≈ 6.8 versus ≈ 23.5 observed. This contrast is left unresolved and likely reflects missing processes in the model (as noted by the authors). I believe that quantifying and attempting to close this mismatch should be part of the main results of the manuscript to go beyond the introduction of a bioinformatics tool for hypothesis generation.

Response: We appreciate the reviewer's insightful comment. We agree that the earlier presentation made the expansion-contraction scenario appear more imposed than it actually was, and we have revised the manuscript to clarify how these phases were chosen and what biological conclusions can, and can't, be drawn from them.

The transition between the expansion and contraction phases was not selected arbitrarily. It was guided by the empirical LTR divergence distribution in A. psidii. Specifically, the prominent K2P divergence peak was used as an approximate temporal marker separating an older period dominated by TE accumulation from a more recent period in which net genome size increase appears to have slowed or reversed. We now state this more clearly in the Methods and Results. We want to clarify that the two-phase model is not meant to be an arbitrary scenario chosen to force a desired outcome. It's a simplified biological hypothesis derived from the observed TE age structure.

We also agree that matching genome size and LTR divergence alone should not be presented as a full validation of the model, because these were among the quantities used to evaluate the simulations. We have therefore revised the language throughout this section. We no longer describe the A. psidii analysis as a recovery of the true evolutionary history. Instead, we present it as a case study showing how PrinTE can be used to test whether a proposed TE-dynamic scenario is capable of producing a genome resembling the observed one across multiple genomic summaries.

*Regarding the solo:intact discrepancy, in our hands, current solo-LTR calls are overly sensitive, which is likely impacted by detection thresholds, sequence divergence, boundary definition, and local assembly/context effects (**Response Figure 6**). As a result, differences in the solo:intact ratio may reflect biological processes, but they may also reflect substantial error in solo-LTR recovery.*

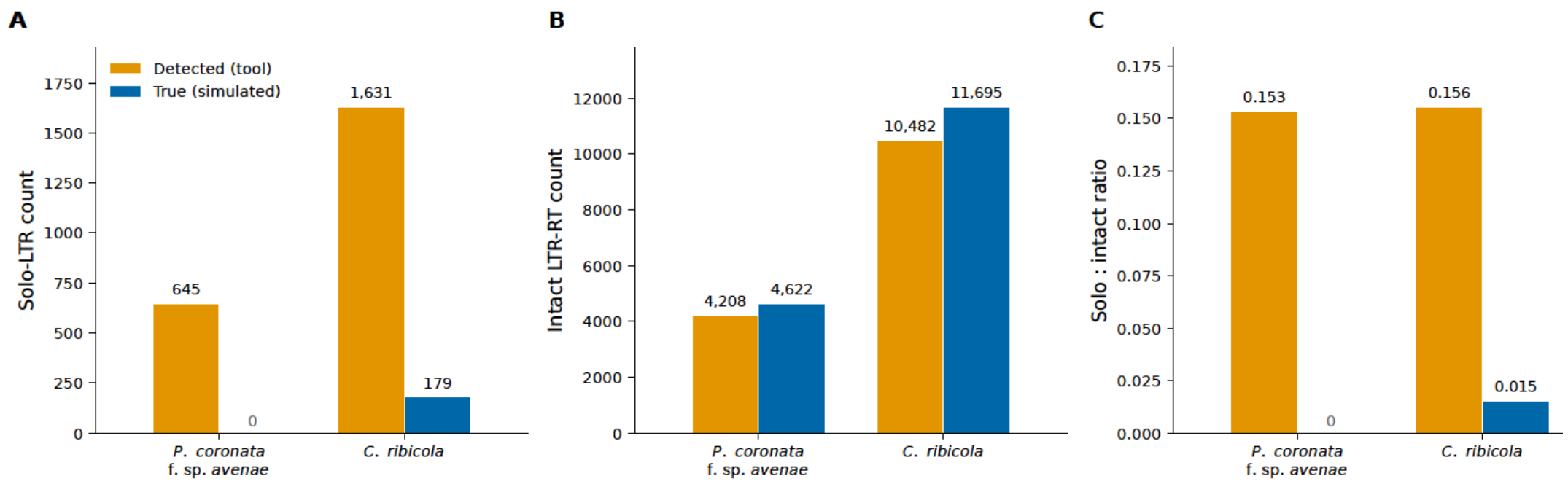

***Response Figure 6:** Solo-LTR and Intact LTR-RT detection in simulated Puccinia coronata f. sp. avenae and Cronartium ribicola genomes highlight the limitation of using empirical solo-LTR annotations to drive hypotheses. **(A)** Solo-LTR and **(B)** Intact LTR-RT counts detected vs simulated truth. **(C)** S:I ratio detected vs simulated truth.*

Other than the detection uncertainty of solo LTRs, our results indicate that the solo:intact LTR ratio should be interpreted cautiously as a proxy for purge pressure, as it may reflect multiple underlying processes beyond the simple removal of LTR-RT sequence. For example, elevated ratios may arise from differential retention of solo LTRs or lineage-specific differences in TE turnover. Thus, improved methods in annotation of solo LTRs and intact elements, together with explicit modeling of TE birth–death dynamics, will be important for disentangling these alternative explanations.

In the revised analysis, we have down-weighted the solo:intact ratio as a fitting criterion. This change was made because solo-LTR detection remains substantially less reliable than detection of intact LTR retrotransposons in both real and simulated genomes. We therefore consider the solo:intact ratio, at the present stage, too noisy to serve as a robust quantitative target for model fitting.

Instead, the revised pipeline evaluates simulations using summaries that are more consistently measurable across real and simulated genomes. These summary statistics include the number of detected LTR retrotransposons, their cumulative genomic length, total genome size, and the distribution of LTR divergence. These metrics better capture the TE landscape for PrinTE modeling, resulting in a more confident quantification of model parameters in the TE-genome evolution hypothesis.

-The paper concludes that a positive correlation between genome size and solo:intact suggests illegitimate recombination alone is insufficient to prevent genome expansion; however, this statement is descriptive and close to trivial unless placed in a quantitative framework that specifies the ranges of insertion, deletion, illegitimate recombination, and mutation rates that cannot hold size constant.
Response: We thank the reviewer for the insightful comment. We agree that the original simulation is somewhat arbitrary and the statement was too descriptive. We now

develop the genome size evolution hypothesis based on the phylogenetic reconstruction of ancestral genome size. The updated PrinTE contains a quantitative framework that can perform efficient parameter sweeping and evaluation of model fitting. Under this framework, the positive correlation between genome size and solo:intact ratio suggests that, within the observed parameter space, recombination-mediated deletion increases with TE activity but is not sufficient on its own to counteract the net genome expansion. We have updated our simulation results in ***Figure 6*** *and the corresponding text and discussion in the revised manuscript.*

-Related to the previous point, LTR-RT removal is modelled via solo-LTR formation at a user-set rate and full deletions for non-LTR TEs; however, complete removals of neighbouring LTR-RTs by illegitimate recombination beyond solo formation are not explicitly parametrised, which can bias solo:intact expectations and age spectra.
Response: We agree that recombination may remove neighboring LTR-RTs or adjacent genomic sequence, and that this could affect solo:intact ratios and inferred age spectra. In the current version of PrinTE, we did not explicitly parameterize multi-element deletion events because broadly applicable estimates of their frequency and size distribution are currently limited. Because of this uncertainty, and the biological and technical complexities of the solo:intact ratio metric, we no longer emphasize simulating genomes with the solo:intact ratio close to the reference. We have clarified this assumption in the manuscript and noted it as a possible direction for future development.

-To my knowledge, PrinTE allows TE truncations and mutations, but does not model the origin and propagation of non-autonomous TEs with altered transposition rules. In fact, non-autonomous TEs typically outnumber autonomous counterparts and contribute disproportionately to TE content. Including an explicit module or at least sensitivity analyses of the emergence and contribution of non-autonomous TEs would be important.
Response: We thank the reviewer for bringing up this important mechanism of TE propagation. PrinTE uses a TE library as input to guide TE insertion, deletion, and evolution during simulation. We can change the abundance of autonomous vs non-autonomous modes of propagation by adjusting the TE library composition. Additionally, PrinTE takes a TE ratios table as input, which is used to guide the abundance of insertion of TE classes and superfamilies of interest. We can also customize autonomous and non-autonomous insertion frequencies using this TE ratios table to simulate evolutionary scenarios that interest the user.

-Population genetic forces such as drift, effective population size, mating system, admixture, polyploidy, and migration are discussed conceptually (implicitly or explicitly)

but not implemented. These factors strongly affect TE load and fixation. I understand that including these parameters may be laborious and out of scope; however, claims should be framed as single-genome, panmictic approximations and not as estimates of force strengths. In other words, the modelling is interesting to generate hypotheses, but it is missing numerous crucial variables that can bias results and interpretations.
Response: We agree that PrinTE does not directly model population genetic forces such as drift, effective population size, mating system, admixture, polyploidy, or migration, and we have revised the text to make this limitation explicit. We now frame PrinTE as a single-genome, panmictic approximation designed to explore how TE parameters may shape genome composition, rather than as a model estimating the strengths of population-level forces. We have also expanded the discussion to clarify that users can interpret and adjust TE parameters in light of these non-modeled biological processes, but that such forces are not directly implemented in the current framework (L711-721).

-Related to the previous point, comparisons to SLiM emphasise sequence-level modelling; however, a hybrid approach (SLiM for population genetics inference + PrinTE for sequence-level consequences) is not explored, and would partially address some of the concerns discussed above.
Response: We thank the reviewer for suggesting a potential solution to population-level simulations. We agree that a hybrid SLiM-PrinTE framework could be valuable for linking population genetic inference with sequence-level consequences. However, the present study focuses on PrinTE as a single-genome approximation, and implementing or documenting a full population-level workflow is beyond its current scope. We have revised the manuscript to note this potential future direction and to clarify that users may combine PrinTE with population genetic tools such as SLiM, although PrinTE does not currently provide built-in support or guidance for such integration.

- It seems that the modelling of DNA transposons does not explicitly include cut-and-paste excision dynamics (excision rates, donor-site footprints/repair, coupling between excision and reinsertion, local hopping vs genome-wide reinsertion). Without an explicit excision module, DNA TE turnover and copy-number dynamics may be misrepresented. It would help to implement (or at least test the impact of ignoring such dynamics) an excision/reinsertion model and clarify the current assumptions used for DNA transposons.
Response: We thank the reviewer for raising this point and agree that explicit cut-and-paste dynamics are important to model for DNA transposons. The current version of PrinTE now includes a conservative cut-and-paste module that explicitly couples excision to reinsertion. Superfamilies flagged as Type II in the TE-ratios file are excluded from the replicative insertion process (which represents Type I, copy-and-

paste, retrotransposons). Excision of an intact Type II element is then interpreted as a transposition event and triggers a same-family reinsertion in the following generation, drawn from the current evolving library. Excision of a fragmented Type II element is treated instead as illegitimate recombination on a dead element and removes it outright with no compensating reinsertion.

We have revised the Results to comment on this edit explicitly. The current model does not parameterise per-family or per-element excision rates separately. The deletion rate applies uniformly to eligible elements, weighted by length and chromatin context as for Type I families. It currently does not simulate donor-site footprints from NHEJ repair. The excised element is removed cleanly aside from the target-site duplication retained at the donor margin. Reinsertion uses the same genome-wide machinery as ordinary insertions, so we do not capture local-hopping cis-preference. We view these updates as significant, but currently out of scope, so we will flag them for potential future improvements (L168-170).

Reviewer #1 (Remarks to the Author):

I find that all the risen concerns were overall properly addressed and that the new manuscript and amended figures are suitable for publication. I appreciate the in-depth revisions that have greatly improved the manuscript.

We thank the reviewer for careful reading across both rounds of review, and for recommendations that improved the revised manuscript.

Reviewer #1 (Remarks on code availability):

I have not tested the code but the github page is clear with enough instructions for installing and running PrinTE.

Thank you. The repository documentation has been updated again in this round to describe the revised cut-and-paste model and the new "--cutpaste_reinsertion" option.

Reviewer #2 (Remarks to the Author):

I thank the authors' effort to address my comments. I have no further concerns.

We thank the reviewer for their constructive comments throughout the review process.

Reviewer #2 (Remarks on code availability):

Maybe i missed it, but adding simple test/demo data would be beneficial for users.

PrinTE requires no user-supplied data to run, which we should have made clearer. The "Quick start" section of the README opens with a self-contained command, "bash PrinTE/PrinTE.sh --burnin_only -sz 100Mb -cn 1 -P 4 -itp 10", which builds a 100 Mb demonstration genome from scratch, followed by a second command that forward-evolves it. Everything those examples need is bundled with the repository. These allow a new user to reproduce a complete simulation immediately after installation, with no downloads and no input files of their own.

Reviewer #3 (Remarks to the Author):

Thank you for the detailed responses and for the substantial revisions. The implementation of broader parameter searches, fit landscapes, and sensitivity analyses clearly improves PrinTE as an informatic tool. However, I still have several concerns that I do not think have been fully addressed.

We appreciate the reviewer's input. Their comments have improved both the model and the conclusions, and we are addressing each point below.

One specific issue concerns the modeling of DNA transposons. The revised implementation couples the excision of an intact Type II element to the reinsertion of an element from the same family, and the manuscript states that cut-and-paste transposons relocate while conserving copy number. Although excision is required for the mobilization of many DNA transposons, they do not necessarily conserve copy number. They can increase in copy number through mechanisms linked to DNA replication and repair. Otherwise, any non-zero deletion rate would lead to their progressive loss and eventual extinction, which is not consistent with the extensive accumulation of DNA transposons observed in many genomes. A strict one-to-one excision–reinsertion model is therefore not realistic. The model should allow the balance between excision and successful reinsertion or amplification to vary, or at least clearly acknowledge this limitation. The statement in Lines 172–173 that DNA transposons conserve copy number during mobilization should also be corrected or qualified.

Thank you for the suggestion. We agree and have changed the model to better match the biology. PrinTE now exposes the balance the reviewer describes as a parameter, "--cutpaste_reinsertion". It is defined as the expected number of successful re-insertions per excision of an intact Class II element. A value of 1.0 reproduces the previous behavior and conserves copy number. Values above 1.0 model the replication- and repair-coupled gains the reviewer notes, which allow DNA transposons to accumulate under a non-zero deletion rate, although values beyond 2.0 fall out of a typical repair-based gain and may represent other mechanisms that increase copy number. Values below 1.0 model unsuccessful re-insertion and net copy-number loss. The expected number of re-insertions is applied per family at each simulation step. This is implemented in the released code and documented in the README. The corresponding implementation details have been added to the manuscript.

I also remain unclear about how PrinTE handles the formation of new non-autonomous elements. The authors explain that users can control the initial abundance of autonomous and non-autonomous elements through the input TE library and TE-ratios table. However, this does not address my original concern, which was about the evolutionary emergence of new non-autonomous derivatives. Can an autonomous element lose its coding capacity during the simulation, become non-autonomous, and then propagate according to different mobilization rules? From the description of the model, TEs appear to accumulate sequence mutations and structural damage, but not to functionally evolve. If this is the case, it is an important limitation because the emergence and amplification of non-autonomous elements are major components of TE evolution.

The reviewer's reading of the model is correct, and we have now stated this limitation. An autonomous element can lose its coding capacity during a simulation. Elements accumulate substitutions and are truncated, nested, and fragmented, and the resulting variants can form new lineages that proliferate, persist at low abundance, or die out. What PrinTE doesn't do is let mutations change how an element mobilizes. Mobilization rules are fixed by family assignment, so a decayed autonomous element does not begin propagating under non-autonomous rules. The balance of autonomous and non-autonomous elements is set by the user.

We have added this to the limitations in the Discussion, together with our reasoning for not yet attempting to model the transition. We also note that the effect the reviewer describes can be approximated with PrinTE's continuation functionality. A simulation can be paused, its TE library and

superfamily ratios revised to introduce or expand non-autonomous derivatives, and the run continued from the same genome. In this way, the transposition dynamic is imposed rather than emerging within a run.

The treatment of the solo:intact LTR ratio also remains inconsistent. In their response, the authors argue that this ratio is too affected by annotation uncertainty to serve as a reliable fitting criterion. However, the manuscript still uses the positive correlation between genome size and the solo:intact ratio to draw conclusions about the ability of recombination-mediated deletion to constrain genome expansion. If this metric is not reliable enough for model fitting, it should not be used to support strong biological conclusions. These interpretations should be removed, substantially toned down, or supported by independent analyses.

Thank you for this point. The revised text now reports the correlation as a descriptive summary of LTR-RT composition rather than as a measure of purging pressure. We no longer interpret it as evidence about the efficacy of recombination-mediated removal and state that the ratio depends on how completely solo LTRs and intact elements are annotated.

We also note in the Methods and the Fig. 7O caption that solo:intact ratios were computed for description and comparison, not for fitting.

Additionally, we took the reviewer's suggestion by supporting the claim with independent analysis. The statement that recombination-based removal does not prevent genome expansion is now supported by the controlled simulations because the rate of solo LTR formation is a directly manipulated parameter in PrinTE. At a fixed deletion rate, increasing the rate at which LTR-RTs are converted to solo LTRs produces larger, not smaller, genomes because the solo LTR remains in the genome after recombination (Fig. 2H, Figs. S3, S4). The Abstract and Results now attribute the claim to the simulations accordingly.

More fundamentally, I still do not clearly see what biological knowledge can be obtained from PrinTE beyond generating evolutionary narratives that are internally consistent with the model. The revised optimization procedure identifies parameter combinations that reproduce selected properties of an observed genome, such as genome size, TE copy number, cumulative TE length, and LTR-divergence distributions. This can show that a particular scenario is possible under the assumptions of PrinTE. However, it does not show that the selected parameters correspond to the real historical insertion, deletion, or recombination rates, or that the proposed scenario resembles what actually happened.

Thank you for the insightful comment. We agree that the best-fit parameters only represent a possible scenario and have revised the manuscript accordingly. We added a new paragraph in the Discussion stating that the best parameters PrinTE returns are compatible with an observed genome under PrinTE's model and clearly stating hat different histories can leave similar present-day signatures.

Despite the uncertainty, the parameter fitting is anchored by summary statistics estimated independently of the simulation: (1) an ancestral state obtained by ancestral state reconstruction on a time-calibrated phylogeny, and (2) summary statistics from a present-day genome. Sweeping the four-dimensional parameter space between those anchors refines the TE turnover hypothesis into a bounded, reproducible, and falsifiable quantification, and it identifies which parameters the data cannot constrain. In our data, the wide bounds on the deletion rate are part of the result. The analyses described below are intended to test this.

Very different evolutionary histories may produce similar present-day genome summaries. The manuscript does not show that PrinTE can distinguish between these alternative histories. For example, there is no parameter-recovery experiment in which genomes are generated using known but complex evolutionary scenarios, the true history is hidden, and PrinTE is then asked to recover the correct parameters and changes through time. Such an analysis would be important to show whether PrinTE can distinguish continuous activity from episodic bursts, changes in insertion rate from changes in deletion rate, or an expansion–contraction history from other processes that generate similar TE-age distributions. Showing that a parameter influences the composite score is not the same as showing that the parameter can be accurately recovered.

Thank you for the great idea. We agree that this is an important analysis. We have performed the experiment as requested. We used PrinTE to forward-evolve a 100 Mb genome for three million generations in 500,000-generation steps under a fixed insertion rate, deletion rate, solo LTR formation rate, and deletion length bias. We then treated the resulting genome as an empirical reference, re-annotated its intact LTR-RTs de novo as we would for a real genome, and ran the Grid Search Method over the same four-dimensional parameter space used for the rust fungal genomes.

Searching four orders of magnitude, the insertion and deletion rates that govern genome size were recovered accurately. From the best-fitting of 650 parameter combinations, we estimated an insertion rate of 2.83×10^{-11} per bp per generation against the true value of 3.0×10^{-11} (a 5.6% error; best-5% range [2.2×10^{-11}, 3.5×10^{-11}]), and a deletion rate of 1.03×10^{-11} against the true value of 1.0×10^{-11} (a 3.2% error; best-5% range [5.7×10^{-12}, 1.9×10^{-11}]). Both ranges contain the true values.

The solo LTR formation rate (true value 95%) and the deletion length bias (true value k = 10) were returned as 15% and 7, and the best 5% of simulations spanned essentially the entire searched range for both. In the sensitivity analysis, PrinTE predicts this outcome because insertion and deletion rates together account for almost all of the explainable variation in the composite score (joint cross-validated $R^2 = 0.94$), while the solo LTR formation rate and the deletion length bias account for none on their own.

The results are reported in the manuscript as Fig. 6, with the original Fig. 6 becoming Fig. 7. We agree with the reviewer that the composite score is not the same as recoverability, and have kept those two claims separate in the revised text.

Distinguishing an episodic burst from sustained activity at a lower average rate is a harder problem that our composite metric is not designed to solve, and we have been careful not to claim it. The manuscript notes this as a limitation explicitly: "The simplification of TE activity fluctuations in PrinTE provides a controlled framework for modeling the effective average rates over the simulated interval" and "It is important to note that the estimated parameters are effective insertion and deletion rates averaged from a long period of evolutionary time, which may not capture short episodic periods of TE bloat and purge". Where the manuscript does propose a change in rate through time, in the two-phase A. psidii analysis, the timing of that change is taken from the empirical LTR-RT divergence distribution (Fig. 4D), which is estimated from the real genome without reference to any simulation, rather than inferred by the parameter search.

The inferred rates are also not compared with independent biological measurements, such as experimentally measured transposition rates, population-level TE insertion polymorphisms,

pedigree or mutation-accumulation data, or independently dated TE bursts. Similarly, the model is not fitted using one set of genomic features and then tested on additional features that were not used for fitting. As a result, the case studies mainly show that the model can be adjusted to reproduce the same genome properties used to score the simulations. This is calibration, but it is not validation of the evolutionary history proposed by the model.

We are grateful for this suggestion. The Composite Distance Metric has four components: (1) LTR-RT copy number, (2) cumulative LTR-RT length, (3) the LTR-RT divergence distribution, and (4) genome size. With the weights used in these analyses ("--alphas 0 0 10 1", PrinTE's default), the first two receive zero weight, so LTR-RT copy number and cumulative LTR-RT length are measured but play no part in scoring, and can serve as held-out additional features for testing. The parameter-recovery test (New Figure 6) reports how well the best-fit genome reproduces those two held-out features in addition to the parameters themselves, to within 4.8% and 3.2%, respectively.

PrinTE is a simulator, and can validate its own machinery but not the biology it represents. We strongly agree with the reviewer and took the great advice not to present the PrinTE simulation as evidence about how TEs behave in nature. PrinTE produces a hypothesis for a real genome and the manuscript is written accordingly. The manuscript does not describe any result as validating an evolutionary process, and the fitted parameters are consistently described as compatible with or as a quantitative hypothesis about the observed genome. The comparisons with independent evidence described above are likewise offered as consistency checks on the inferred rates rather than as confirmation of them. When the direct measurements the reviewer lists become available for these lineages (or any lineages), they will be able to compare them to the rates we report. To further clarify the interpretation of the simulation, we have extended the Discussion section regarding the inferred rates.

I therefore think the manuscript still overstates the biological meaning of the fitted parameters. Statements suggesting that PrinTE quantitatively estimates historical TE turnover rates, resolves genome-size evolution, demonstrates how expansion or contraction occurred, or uncovers the evolutionary trajectory of a lineage are not supported by the current analyses. At present, the outputs should be described more cautiously as parameter combinations that are compatible with selected present-day genome summaries under the assumptions of PrinTE.

PrinTE may be an interesting informatic tool for sequence-level simulation, benchmarking, and hypothesis generation. However, its ability to provide biological insight has not yet been clearly demonstrated. Unless the authors provide parameter-recovery tests, comparisons between genuinely different evolutionary scenarios, independent validation, or predictions of genomic features not used during fitting, statements claiming that the model "demonstrates, resolves, reveals, or reconstructs particular evolutionary processes" should be removed or substantially toned down. In my opinion, the current analyses show that certain narratives can reproduce the observed data within the model, but they do not show that these narratives reflect biological reality.

We appreciate all the suggestions and comments from the reviewer; some are difficult to address due to the availability of data, but all are highly valuable. We have modified the text according to the comments.

Those edits are highlighted in the manuscript. Throughout the revision, we now present PrinTE's output as a quantitative hypothesis about genome size evolution rather than as a reconstruction of what happened. The search returns the parameter combinations compatible with an observed genome under PrinTE's model, bounded at one end by an independently reconstructed ancestral state and at the other by present-day summary statistics, and it reports which of those parameters the data can and cannot constrain. Framed this way, the contribution is that a verbal narrative of TE turnover becomes a set of numbers with stated bounds that independent data can test.

We thank Reviewer #3 again. The request to demonstrate recoverability has made this a considerably better study, and the revised cut-and-paste model is a genuine improvement to the software that will benefit its users. We hope the revised manuscript is now suitable for publication in Nature Communications.
